# Abrupt permafrost thaw drives exceptional carbon release across the Tibetan Plateau

**DOI:** 10.1038/s41467-026-74850-y

**Published:** 2026-06-27

**Authors:** Yunjie Jia, Chunling Zhang, Mei Mu, Xinlong Du, Jinyue Song, Yuguo Wei, Yongqi Ge, Kun Li, Hebin Liu, Pengsi Lei, Xiaoqing Peng, Zhuoxuan Xia, Lingcao Huang, Rongkun Liu, Sonam Wangchuk, Yuanhe Yang, Lin Liu, Cuicui Mu

**Affiliations:** 1 Key Laboratory of Western China’s Environmental Systems (Ministry of Education), College of Earth and Environmental Sciences, Observation and research station on Eco-Environment of Frozen Ground in the Qilian Mountains, Lanzhou University, Lanzhou, China; 2 State Key Laboratory of Herbage Improvement and Grassland Agro-ecosystems, College of Ecology, Lanzhou University, Lanzhou, China; 3 State Key Laboratory of Frozen Soil Engineering, Northwest Institute of Eco-Environment and Resources, Chinese Academy of Sciences, Lanzhou, China; 4 Department of Earth and Environmental Sciences, Faculty of Science, The Chinese University of Hong Kong, Hong Kong, China; 5 Institute of Space and Earth Information Science, The Chinese University of Hong Kong, Shatin, Hong Kong SAR, China; 6 International Centre for Integrated Mountain Development, Kathmandu, Nepal; 7 State Key Laboratory of Forage Breeding-by-Design and Utilization; Key Laboratory of Vegetation and Environmental Change, Institute of Botany, Chinese Academy of Sciences, Beijing, China

**Keywords:** Cryospheric science, Environmental impact

## Abstract

Climate warming is accelerating abrupt permafrost thaw, driving substantial carbon emissions. Retrogressive thaw slumps (RTSs) represent the most severe instance of abrupt permafrost thaw, yet their carbon emissions remain poorly quantified due to limited observations. Here, by synthesizing 4728 RTS incidents and 1862 in-situ CO_2_ and CH_4_ measurements from RTS-affected zones across the Tibetan Plateau, we estimate that the area of RTS susceptibility will expand by 17–19% by 2100 relative to 2022, driven primarily by precipitation changes. Compared to control areas, the ecosystem respiration rate in collapsed areas decreases by 14.4%, while CH_4_ release rate increases by 20.0%. The combined CO_2_ and CH_4_ release associated with RTS expansion increased 1.1-fold between 2016 and 2022. Under the intermediate Shared Socioeconomic Pathways scenario, carbon emissions from RTS-susceptible areas are projected to surge 2.7-fold by 2100. These findings highlight that abrupt thaw strengthens permafrost carbon-climate feedback in high-altitude regions, underscoring the urgent need for targeted permafrost protection strategies to achieve carbon neutrality goals.

## Abstract (web summary)

This study synthesizes 4,728 retrogressive thaw slump (RTS) incidents and 1,862 field carbon flux measurements on the Tibetan Plateau to quantify the dynamics of RTS susceptibility and project RTS-induced carbon release will rise 2.7-fold by 2100.

## Introduction

Permafrost region, covering approximately 22% of the Northern Hemisphere land area^1^, which contains 1460–1600 Pg of soil organic carbon^2,3^, is undergoing extensive degradation due to global warming^4,5^. As the most severe instances of abrupt permafrost thaw, retrogressive thaw slumps (RTSs) are accelerating expansion due to the melting of ground ice^6–10^. RTSs represent one of the typical hillslope thermokarst landscapes, characterized by the melting of ground ice and the subsequent subsidence or collapse of thawed material^11,12^. They can destabilize hillslope systems and expose several meters of sediments deposited during permafrost aggradation^12^, which exert substantial impacts on carbon release processes^13–16^. Particularly, RTSs can release large amounts of CO_2_ and CH_4_ into the atmosphere by significantly altering microtopographic conditions and soil hydrological processes^16–18^. This carbon release further accelerates global warming, potentially forming a positive climate feedback loop^5,19,20^. Notably, across the Circum-Arctic region, abrupt thaw-driven carbon release is projected to account for approximately 40% of that from gradual permafrost thaw. This substantial contribution is primarily derived from newly formed thermokarst landscapes, which cover less than 5% of the northern permafrost areas^21^. Moreover, the impacts of RTSs on carbon release remain poorly represented in Earth System Models^22^, and have not yet been integrated into national carbon neutrality targets. However, owing to sparse observational evidence, no study has quantified carbon release from high-altitude permafrost regions, hampering our ability to assess the regional and global permafrost carbon-climate feedback^23^.

The Tibetan Plateau contains the largest high-altitude permafrost area globally^1^, accounting for approximately 75% of the total alpine permafrost area^24^. It is experiencing rapid warming at a rate about twice that of the global average^25^. During 2016–2022, the observed RTSs on the Tibetan Plateau has developed rapidly, with the number increasing from 1590 to 3800 and the affected area surging 3.8-fold^26^. The expansion of RTSs depends on various environmental factors such as air temperature, precipitation, ground ice, active layer thickness, ground temperature, and soil texture^27,28^. However, quantifying the drivers of RTSs and their spatial variations remain unclear, which leads to the uncertainty in simulating their evolution processes^29^, thus further affecting the accurate assessment of carbon release. Although several preceding experimental studies have focused on measuring CO_2_ and CH_4_ release rate, and soil organic carbon loss by following RTSs on the Tibetan Plateau^14,17,30,31^, they are usually at specific site or small regional scale due to the harsh environment and high heterogeneity in high-altitudes^32,33^. Thus, the systematic estimation of RTS-induced CO_2_ and CH_4_ release across the entire Tibetan Plateau has not been explicitly explored.

**Fig. 1:**
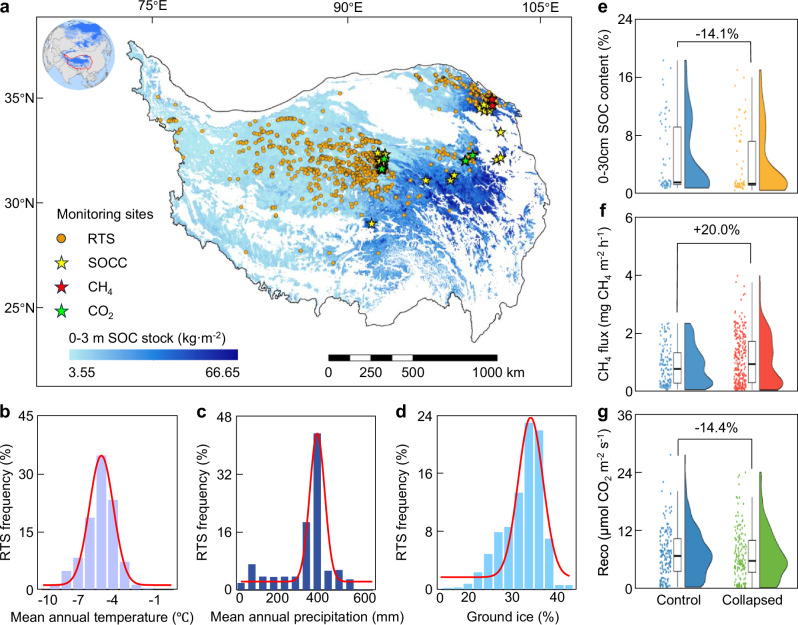
Observed distribution of retrogressive thaw slumps (RTSs) and their impacts on carbon release across the Tibetan Plateau. **a** Geographical distribution of in-situ RTSs, carbon release rates (CH_4_ and CO_2_) and soil organic carbon content (SOCC), with the background of soil organic carbon stocks at 0–3 m depth within the permafrost area of the Tibetan Plateau^33^. The study extent is derived from Zou et al., 2017^61^. Orange dots indicate the RTS locations; red, green, and yellow stars represent monitoring sites for CH_4_, CO_2_ and SOCC, respectively. **b-d** Distribution frequencies of observed RTSs with respect to mean annual air temperature **b**, mean annual precipitation **c**, and ground ice content at depths of 2–10 m **d**. The curves show the fitted frequency distribution. **e-g** Comparisons of growing season mean SOCC in the upper 30 cm **e**, CH_4_ release rate **f**, and ecosystem respiration (Reco) **g** between control and collapsed areas of RTSs. Notably, CH_4_ flux data are focused on alpine swamp meadows. The violin plots illustrate the magnitude and distribution density of the measured variables for control and collapsed areas. The boxes represent the median and interquartile ranges, and the shaded areas represent the frequency distribution of the data.

To address this knowledge gap, we compile a comprehensive dataset including 4728 RTS incidents, 1862 in-situ measurements of CO_2_ and CH_4_ emission rates, and 291 records of soil organic carbon content (SOCC) loss from 61 RTS sites across the Tibetan Plateau (Fig. 1, Supplementary Table 1). These monitoring sites are widely distributed across the three dominant vegetation types in the permafrost region, including alpine swamp meadow, alpine meadow, and alpine steppe. First, we show the distribution of field-observed RTSs and quantify their impacts on carbon release. Using machine learning models incorporating ten environmental variables associated with RTS dynamics, we then examine the drivers of RTSs development and show the spatiotemporal patterns of RTS susceptibility across multiple risk levels. Subsequently, we estimate carbon emissions from RTSs between 2016 and 2022, and calculate carbon release based on spatially explicit RTS susceptibility. Finally, Shared Socioeconomic Pathways (SSPs) scenarios are employed to explore the projected changes in carbon release associated with future RTS susceptibility by 2100. We highlight the critical role of abrupt permafrost thaw in the carbon balance of alpine ecosystems. This study provides valuable insights for systematically evaluating carbon release caused by abrupt thaw at high altitudes and supports the accurate assessment of terrestrial carbon-climate feedback.

## Results and discussion

### Impacts of observed RTSs on carbon release

By synthesizing 4728 in-situ RTS incidents on the Tibetan Plateau (Fig. 1a), our analyses indicate that more than 50% of RTSs occur in regions with mean annual air temperature ranging from –6 °C to –2 °C and mean annual precipitation of 300–400 mm (Fig. 1b–c). Approximately 90% of RTSs are distributed in areas with ground ice content higher than 20% (Fig. 1d). Roughly 91% of RTSs are smaller than 0.06 km^2^, and 63% of RTSs occur on slopes of 3° to 9° (Supplementary Fig. 1). Furthermore, based on monitored 61 RTS sites (Supplementary Table 1), we find that RTS development reduces soil organic carbon contents (SOCC) in the upper 30 cm layer by 14.1% (Fig. 1e). Notably, pronounced vertical differences in surface SOCC loss occur across the three vegetation types. In alpine swamp meadows and alpine steppes, SOCC loss decreases progressively with depth: loss of 13.4% and 14.6% in 0–10 cm layer, 13.7% and 14.4% in 10–20 cm, and 10.1% and 6.4% in 20–30 cm, respectively. In contrast, alpine meadows exhibit no significant vertical variation, with the SOCC losses of 18.3–19.0% (Supplementary Fig. 2). Alpine steppes exhibit relatively low carbon loss, despite experiencing dramatic hydrothermal changes during RTS development (a 65.3% increase in soil temperature and a 238.9% decrease in moisture; Supplementary Fig.3). By contrast, alpine swamp meadows contain much higher soil carbon stocks and thus exhibit greater carbon loss despite even with milder hydrothermal alterations. These contrasting patterns highlight that vegetation type strongly mediates soil carbon loss induced by RTSs.

We further quantified the impacts of RTSs on carbon release rates across the Tibetan Plateau permafrost region using 1862 in-situ measurements of CO_2_ and CH_4_ release rates. For CH_4_ emissions in alpine swamp meadow, the release rate during the growing season (May–September) reaches 163.2 ± 24.5 g C m⁻² yr⁻¹, which is 2.4 times higher than that in the non-growing season (Supplementary Fig. 4). Relative to control areas, the CH_4_ release rate in RTS-affected areas increases by 20.0% (Fig. 1f). For ecosystem respiration (Reco), measured across three vegetation types (alpine swamp meadow, alpine meadow, and alpine steppe), collapsed areas exhibite a 14.4% reduction compared with control areas, as plant respiration is largely absent following vegetation loss in RTS-affected regions (Fig. 1g). Across vegetation types, the mean ecosystem respiration values during growing season are 320.6 ± 105.1 g C m⁻² yr⁻¹ in alpine meadow and 57.3 ± 13.7 g C m⁻² yr⁻¹ in alpine steppe (Supplementary Fig. 4). In alpine swamp meadow, ecosystem respiration during the growing season is 1416.1 ± 117.1 g C m⁻² yr⁻¹, approximately 4.6 times higher than that in the non-growing season (Supplementary Fig. 4). Nevertheless, both CO_2_ and CH_4_ release rates in the collapsed areas are comparable to those in the control areas during the non-growing season (Supplementary Fig. 5). By synthesizing extensive observations, our study provides a systematic quantification of RTS-induced changes in soil organic carbon content and carbon release across the Tibetan Plateau.

The release of CO_2_ and CH_4_ from RTSs is mainly driven by changes in soil hydrothermal conditions^16,34^. Soil temperatures within RTS collapsed areas are consistently higher than in control areas^35–37^ (Supplementary Fig. 3a). This thermal anomaly, together with enhanced microbial diversity in collapsed areas, increases the temperature sensitivity of CO_2_ release^31,38,39^, and thus stimulates heterotrophic respiration^38,40^. Concurrently, reduced vegetation cover lowers gross primary production (GPP)^34,41^, causing collapsed areas to act as stronger carbon sources. Furthermore, the naturally high soil moisture typical of alpine swamp meadows (Supplementary Fig. 3b), combined with continuous upslope water supply in mountainous RTSs^39^, leads to only a minor reduction in soil moisture ( ~ 1.9%) in collapsed areas^41^ (Supplementary Fig. 3b). This preserves favorable anaerobic conditions that facilitate CH_4_ emission. Consequently, under the combined influence of these hydrothermal dynamics, carbon release during the non-growing season shows no significant difference between control and collapsed areas. Collectively, this study offers robust observational constraints for reliably quantifying carbon release associated with abrupt thaw in high-altitude permafrost regions.

### Drivers of RTS susceptibility

**Fig. 2:**
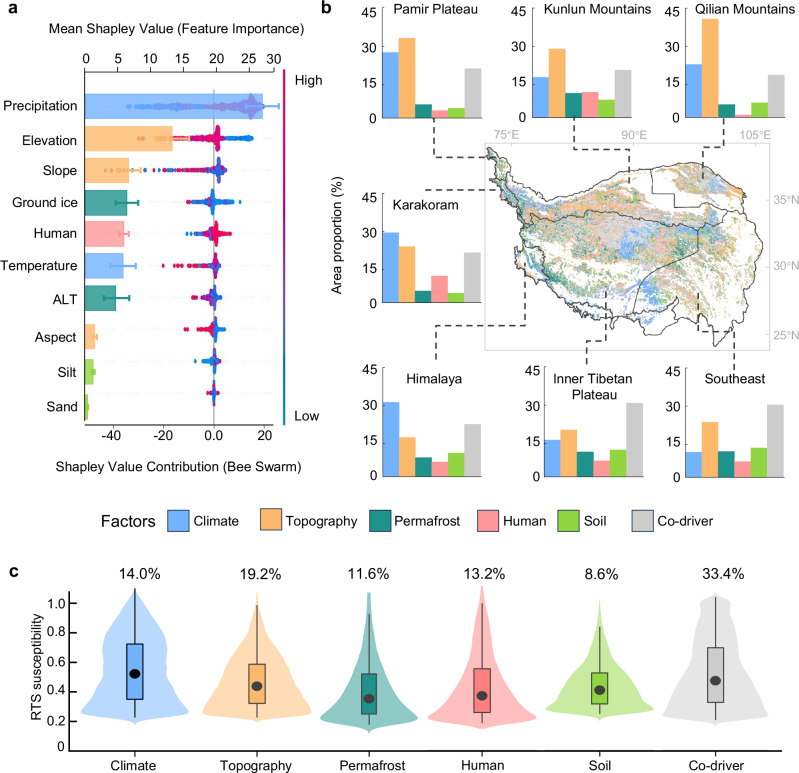
Dominant drivers of retrogressive thaw slump (RTS) susceptibility. **a** Relative importance of environmental variables (climate, topography, permafrost conditions, human activity and soil properties) controlling RTS susceptibility derived from the SHapley Additive exPlanations (SHAP) analysis. The horizontal bars represent the mean absolute SHAP values of each feature, corresponding to the upper x-axis and indicating the overall importance of each feature. Error bars indicate the standard deviation of the mean absolute SHAP values. The dots represent individual samples in the SHAP bee Swarm plot, with their positions along the lower x-axis showing the SHAP value contribution to the model output. Positive SHAP values indicate that the feature increases the predicted RTS susceptibility, whereas negative values indicate that the feature decreases the predicted RTS susceptibility. The color bar indicates the actual feature values of individual samples, with blue representing low values and red representing high values. Active layer thickness (ALT) denotes the thickness of the seasonally thawed layer above permafrost. **b** Spatial distribution of dominant driver categories. The co-driver category denotes regions where RTSs are jointly modulated by all five drivers. Inset bar charts show the area proportion governed by each dominant driver across different mountainous regions. **c** Violin plots depicting the probability density of RTS susceptibility per km^2^ across distinct dominant driver categories. Internal boxes indicate the median and interquartile ranges, with percentages representing the areal fraction corresponding to each driver.

Furthermore, to quantify the impact of RTSs on carbon release, we evaluated the environmental controls governing the spatial distribution of RTS susceptibility, including climate, topography, permafrost conditions, human activity and soil properties (Methods; Supplementary Fig. 6). Our results indicate that climate and topography exert dominant controls, with mean annual precipitation being the most important predictor, explaining 25.2% of the total variance in RTS susceptibility (Fig. 2a). Rising air temperatures accelerate permafrost degradation and ground-ice thaw, thereby weakening slope stability and preconditioning ice-rich slopes for failure^42,43^. In addition, increased precipitation saturates soils under favorable topographic settings, elevates pore-water pressure and reduces shear strength, ultimately triggering RTS initiation under gravitational forcing^9^. Particularly, extreme rainfall is a major driver of permafrost change and can accelerate active-layer thaw^44^. Analogous precipitation-mediated controls on RTS development have also been documented in Arctic permafrost regions^9,12^.

Nonlinear relationships were identified between RTS susceptibility and each individual environmental variable (Supplementary Fig. 7). Specifically, RTS susceptibility reaches a maximum in regions with a mean annual precipitation of 300–400 mm, consistent with the observed spatial distribution of RTSs across the plateau^45^. Notably, the dominant drivers of RTS susceptibility vary substantially across different mountainous regions of the Tibetan Plateau (Fig. 2b). For instance, climatic conditions exert the primary control over RTS susceptibility in relatively arid zones such as the Karakoram and Himalaya. In contrast, topographic factors dominate in the Qilian Mountains, Kunlun Mountains and Pamir Plateau, presumably because they modulate terrain conditions conducive to the formation and preservation of ground ice^8^. In Inner Tibetan Plateau, the main region of RTS occurrence, susceptibility is jointly modulated by multiple environmental factors. Collectively, RTS susceptibility across the Tibetan Plateau is strongly co-regulated, with interactive effects of multiple drivers accounting for 33.4% of the total area (Fig. 2c). Unlike previous studies, our analysis reveals the spatially contributions of various drivers across the Tibetan Plateau. Characterizing the spatial heterogeneity of these controlling factors is essential for robust assessment of RTS susceptibility in high-altitude permafrost environments.

### Spatiotemporal variations of RTS susceptibility

**Fig. 3:**
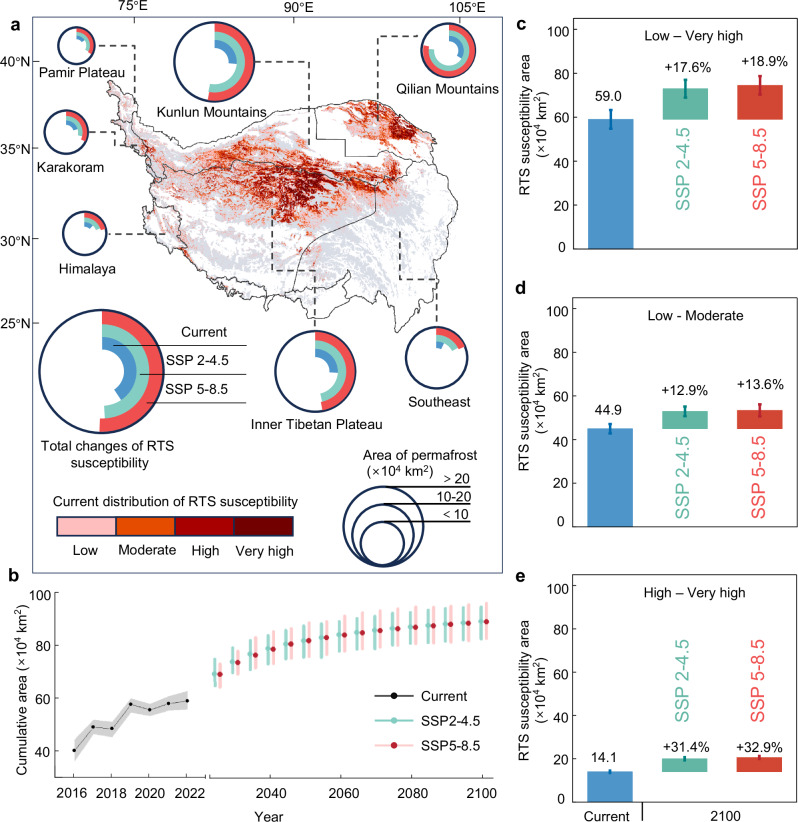
Variations in retrogressive thaw slump (RTS) susceptibility. **a** Current spatial distribution of RTS susceptibility, classified into four levels: low, moderate, high, and very high. The donut chart illustrates the areal proportion of RTS-affected regions across different mountain regions; circle size corresponds to the permafrost area within each mountain region, and blue shading denotes the current extent of RTS susceptibility. Green and red arcs represent projected RTS susceptibility areas by 2100 under SSP2-4.5 and SSP5-8.5, respectively. Grey shading represents permafrost distribution, derived from Obu et al. (2019)^1^. **b** Temporal changes in simulated RTS susceptibility area from 2016 to 2100. The left panel shows area changes derived from observed RTSs during 2016–2022. The right panel shows cumulative area changes within RTS-affected zones under future climate scenarios, with future susceptibility displayed at 5-year intervals for SSP2-4.5 and SSP5-8.5. Points indicate mean values, and thick vertical bars represent uncertainty ranges quantified by the standard deviation. **c–e** Current and projected RTS susceptibility areas by 2100 for low to very high **c**, low to moderate **d**, and high to very high **e** levels. Error bars represent prediction uncertainty expressed as the standard deviation. Percentages above the bars indicate mean changes in RTS susceptibility area under different climate scenarios.

To quantify the impact of RTSs on carbon release, we estimated the area of RTS susceptibility and classified their occurrence probability into low to very high levels using the equal interval method. The results show that the area of RTS susceptibility is 590,396 ± 27,743 km² (Fig. 3a and Supplementary Fig. 8), accounting for roughly 40% of the permafrost area on the Tibetan Plateau. Among these areas, regions with high to very high susceptibility levels occupy roughly 9% of the permafrost region (Fig. 3a). High uncertainty of RTS susceptibility is observed in regions with sparse monitoring sites, such as the Pamir Mountains, Karakoram Range, and Southeast (Supplementary Fig. 9). During 2016–2022, the area of RTS susceptibility expands at a rate of 3.1×10^4 ^km^2 ^y^-1^ (Fig. 3b), which corroborates previous findings based on remote sensing observations^26^. Compared with 2022, the total area of RTS susceptibility is projected to increase by 17–19% under the SSP2-4.5 and SSP5-8.5 scenarios by 2100 (Fig. 3b). Overall, the classified statistics of RTS susceptibility are broadly consistent across the two scenarios; however, this similarity does not imply the absence of scenario-dependent differences. Instead, it primarily reflects the information compression induced by the equal-interval discretization of the continuous susceptibility index, coupled with the nonlinear responses of RTSs to environmental drivers (Supplementary Fig. 7). Although this study clarifies the spatial variations of the five drivers, physical process models of RTSs are urgently needed for accurate predication under climate change. Nevertheless, we identified a notable variation in RTS susceptibility across different occurrence probability levels. For instance, the area of RTS susceptibility across low to very high levels is projected to increase by 17–19% by 2100 (Fig. 3c). In contrast, the area with low to moderate susceptibility levels will increase by 12–14% (Fig. 3d), whereas the area with high to very high susceptibility levels will expand by 31–33% (Fig. 3e). Despite limitations in RTS simulation, the primary objective of this study is to quantify the expansion of RTSs and delve into providing the temporally and spatially explicit variations in RTS susceptibility. These findings are crucial for estimating carbon release induced by abrupt thaw on the Tibetan Plateau.

### Estimated carbon release from RTSs

**Fig. 4:**
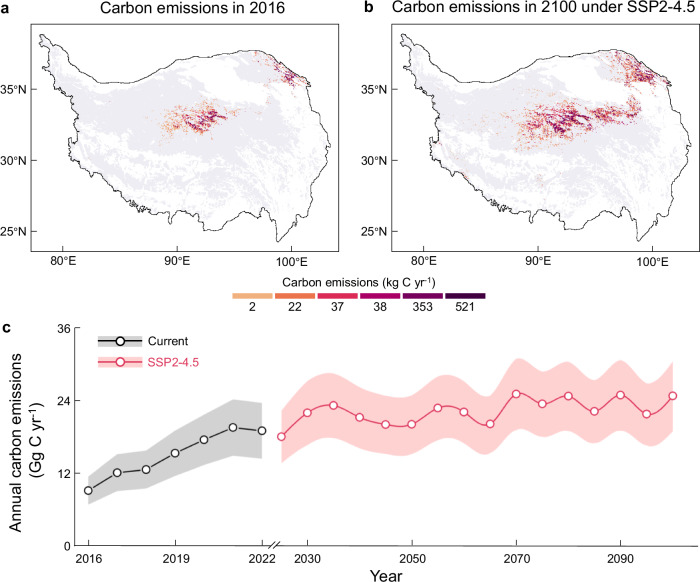
Carbon emissions from retrogressive thaw slump (RTS) susceptibility areas during 2016-2022. **a, b** Spatial distribution of carbon emissions from RTS susceptibility in 2016 **a** and 2100 **b** under the SSP2-4.5 scenario. **c** Projected annual total carbon emissions from RTS susceptibility under the SSP2-4.5 scenario. The shaded areas denote the uncertainty calculated via error propagation.

To quantify the current and future impacts of RTS expansion on carbon release across the permafrost region of the Tibetan Plateau, we estimated the annual total carbon emissions induced by RTSs by synthesizing CO_2_ and CH_4_ release rates under three vegetation types, combined with the spatial distribution of RTS susceptibility at high and very high levels (Methods). Our results show that the mean carbon emission rate from RTSs in 2016 was 92.1 ± 0.5 kg C yr⁻¹, corresponding to a total carbon emission of 9.2 ± 2.3 Gg C yr⁻¹ (Fig. 4a, c). Among the three vegetation types, alpine meadow contributes the highest proportion (approximately 90.7%), followed by alpine steppe (5.1%) and alpine swamp meadow (4.2%) (Supplementary Table 2). Owing to the expansion of areas with RTS susceptibility, total carbon emissions reach 19.0 ± 4.6 Gg C yr⁻¹ by 2022, representing a 1.1-fold increase compared with 2016 (Fig. 4). Notably, the temporal variation of carbon emissions derived from RTS susceptibility shows high consistency with those estimated from actual RTSs during 2016–2022 (Supplementary Fig. 10), which validates the reliability of the susceptibility-based approach for estimating RTS-induced carbon release.

**Fig. 5:**
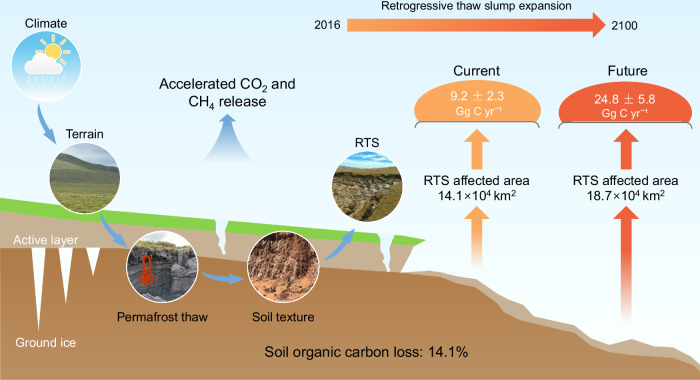
Schematic illustrating the development of retrogressive thaw slumps (RTSs) and their impacts on carbon release across the Tibetan Plateau. The RTS-affected areas are defined as regions classified as high and very high susceptibility zones. Future carbon release corresponds to estimates under the SSP2-4.5 by 2100.

By 2050, total carbon emissions under the SSP2-4.5 and SSP5-8.5 scenarios will reach 20.1 ± 4.8 Gg C yr⁻¹ and 21.3 ± 5.1 Gg C yr⁻¹, respectively, representing a 1.2-fold and 1.3-fold increase compared to the 2016 baseline (Fig. 4c, Supplementary Fig. 11). By 2100, RTS-induced carbon release under SSP2-4.5 will reach 24.8 ± 5.8 Gg C yr⁻¹, which is 2.7-fold higher than the 2016 (Fig. 4c). This study represents the assessment of potential carbon release associated with the future expansion of RTSs across the Tibetan Plateau. Although annual carbon release from RTS-susceptible areas accounts for a small fraction of the net ecosystem carbon sink due to the limited spatial extent of RTSs^46^, the cumulative impacts on vegetation, carbon dynamics, hydrological processes, and microbial communities warrant heightened attention, given the severe environmental perturbations accompanying RTS development. Furthermore, the harsh alpine environment of the Tibetan Plateau results in slow vegetation recovery following disturbance^47^. Consequently, the expansion of RTSs under climate warming is likely to threaten the ecosystem carbon balance in this high-altitude region. Since 2015, frequent and abrupt cryospheric disturbances have been observed across the Tibetan Plateau^48^, indicating that the cryosphere acts as a tipping element under the 1.5 °C global warming threshold^49^. Given that permafrost degradation is expected to accelerate substantially under future climate scenarios^50^, there is an urgent need to systematically quantify and predict carbon release driven by abrupt permafrost thaw (Fig. 5).

Future changes in the RTS susceptibility and associated carbon release remain highly uncertain, due to pronounced spatial heterogeneity across the mountain permafrost zone of the Tibetan Plateau. This heterogeneity stems from regional variations in precipitation regimes, topographic setting and ground-ice conditions. Such spatial complexity in future RTS dynamics further introduces substantial uncertainties into projections of regional carbon emissions. Despite these uncertainties, this study offers distinct advantages. We compiled a comprehensive dataset comprising 1,862 in-situ measurements of CO_2_ and CH_4_ emission rates and 291 observations of SOCC loss from a variety of RTS sites, enabling more robust regional carbon budget estimates across the plateau. Importantly, the dataset incorporates filed observations during the non-growing season, which are critical for constraining estimates of annual carbon release. Furthermore, validation against that from actual observed RTS distributions confirms the reliability of our simulations for carbon release driven by RTS susceptibility. This study provides a solid foundation for future comprehensive assessments of carbon release caused by permafrost degradation under climate change.

### Implications

This study systematically quantified carbon release from abrupt permafrost thaw on the Tibetan Plateau by synthesizing extensive field observations, mapping the spatiotemporal distribution of RTSs, and estimating carbon release from these slumps. Moreover, we evaluated the carbon release caused by RTS expansion using a susceptibility framework that reflects the combined influences of climatic and environmental variables, as well as permafrost thaw dynamics. Recent advances in Earth System Models highlight the critical role of abrupt permafrost thaw in the terrestrial carbon cycle, particularly the impacts of RTSs, which remain poorly constrained in current assessments^23,51^. These efforts not only integrate available observations from high-altitude regions but also provide a comprehensive evaluation of carbon emissions from abrupt thaw, therefore contributing to refine regional and global climate change projections.

Despite synthesizing 4728 RTS incidents and 1862 in-situ observations of carbon release rates, this study remains subject to multiple sources of uncertainty. First, the predicted spatial susceptibility of RTSs is potentially influenced by uncertainties in the RTS inventory, environmental predictors, model parameterization, and assumptions under future climate scenario. Second, the developmental stage of RTSs was not incorporated into our estimation of carbon loss. As RTSs evolve, surface soil organic carbon loss tends to increase during early developmental stages and subsequently decreases^18^. In addition, RTS development can cause substantial displacement and accumulation of deep soils^13^, influencing soil porosity and microbial communities^52^ and thereby modulating carbon release. Consequently, there is an increasing need for future work to develop integrated carbon process models that account for the multi-factorial dynamics of abrupt thaw. Given the spatial heterogeneity in ground ice distribution^53^, the development of some RTSs may gradually stabilize, potentially enabling slow vegetation regrowth in affected areas^36^, which could further influence regional carbon source-sink dynamics^54,55^. Furthermore, the three-dimensional complexity of abrupt thaw necessitates a holistic framework to quantify carbon release across the entire process of RTS development. For instance, the volume of the collapsed body changes dynamically throughout RTS evolution, directly influencing real-time carbon release fluxes^56^. Improving process-based simulations of RTS-induced carbon release on the Tibetan Plateau can provide valuable insights for other mountain permafrost regions to systematically estimate permafrost carbon-climate feedback.

Beyond the estimation of carbon release, projected expansion of RTS susceptibility provides an important foundation for coping with climate change. It was shown that the Tibetan Plateau acted as an appreciable greenhouse gas sink that almost compensated for its contemporary anthropogenic release, making it nearly climate-neutral^57^. To achieve long-term carbon neutrality target, it is critical to implement mitigation initiatives to reduce carbon release from abrupt permafrost thaw. For example, according to our estimated hotspots of RTS development, there is a growing need to implement ecological restoration projects that focus on cultivating dominant species and enhancing vegetation diversity to combat soil erosion caused by RTSs^58^. The findings will deepen our understanding of the coupling dynamics between RTS physical process and biogeochemical cycles, and clarify the key areas for urgent ecological restoration. By adopting proactive and nature-based measures in the future, governments and stakeholders would better safeguard the permafrost environment and enhance carbon sequestration to meet neutrality goals.

## Methods

This study first presented the actual distribution of 4728 RTSs and their impacts on soil carbon loss and greenhouse gas emissions, based on in-situ observations on the Tibetan Plateau. Subsequently, we processed the RTS inventory dataset and data for 10 environmental variables. Further, a suite of single and ensemble machine learning models was employed to estimate the pattens and drivers of RTS susceptibility, and to generate projections of RTS susceptibility under current and future climate scenarios. Finally, carbon release associated with RTS development was estimated (Supplementary Fig. 6). Specifically, this method encompasses five components: data collection and processing, model construction and validation, pattens and driving factors of RTSs, RTS susceptibility mapping and future projections, and estimation of carbon release.

### Retrogressive thaw slump (RTS) inventory

RTSs are slope failures caused by the thawing of ice-rich permafrost, typically consisting of a sub-vertical ice-rich headwall and gentle slump floor composed of meltwater and soil^26^. It is a common landform associated with permafrost degradation^28,59,60^. In this study, we compiled annual RTS inventories across the Tibetan Plateau from published literatures over the period from 2016 to 2022^8,26^, covering a total of 4728 RTS incidents based on field observations and remote sensing data. The dataset for the Qilian Mountains includes 1,064 RTS incidents from 2008 to 2022, primarily identified through manual visual interpretation using Google Earth Pro and Omap, with additional support from high resolution ( < 1 m) imagery from Esri Wayback Imagery^8^. The Tibetan Plateau dataset comprises 3,805 RTS incidents from 2016 to 2022^26^ and the data production processes follow three steps: 1) preprocessing PlanetScope scene; 2) automatically detecting RTSs using the You Only Look Once version 4 (YOLOv4) and manually delineating boundaries; and 3) assessing accuracy by comparing with the field data obtained since 2018 at actual RTS sites (including aerial photographs captured by UAVs, on-site photographs, and field observations). During synthesizing the two datasets, we removed duplicates and organized the annual RTS data during 2016–2022^26^.

### Field observations and carbon release data

To investigate the impacts of RTSs on carbon release, we conducted in-situ field monitoring at 43 RTS sites across the Tibetan Plateau. These sites cover three dominant alpine vegetation types: alpine swamp meadow, alpine meadow, and alpine steppe. The monthly mean data of carbon release was shown in Supplementary Dataset S1. We monitored soil organic carbon content (SOCC), ecosystem respiration (Reco), and CH_4_ release rate. To ensure standardized sampling design and data comparability, each RTS was partitioned into three functional distinct areas: (1) a control zone (an undisturbed permafrost area situated at least 5 meters away from the RTS boundary), (2) a drape zone (a vegetated area adjacent to the slump margin or distributed within the debris tongue), and (3) an exposed zone (vegetation-free bare soil areas). For each zone, a minimum of three replicate sampling points were systematically established (Supplementary Fig.12). A total of 145 soil samples were collected during June–September of 2021 and 2023 from three depth intervals (0–10 cm, 10–20 cm, 20–30 cm) at each site in this study.

Ecosystem respiration (Reco) was measured during April–October from 2021 to 2023 on 17 RTSs using a Li‑8100 automated soil CO_2_ flux system (Li‑Cor Inc., Lincoln, NE, USA) equipped with a shaded chamber. Each measurement lasted 3 minutes, providing direct estimates of CO_2_ release rate along with concurrent soil temperature and moisture at 10 cm depth. Simultaneously, CH_4_ release rates were monitored on four RTSs in alpine swamp meadows using a static closed-chamber technique. Gas samples (25 mL each) were collected from the chamber headspace at 0, 10, 20, and 30 minutes after closure and analyzed for CH_4_ concentration by gas chromatography. Overall, we collected 1,095 Reco measurements and 382 CH_4_ release measurements for RTSs on the plateau.

Additionally, we performed a literature review using the Web of Science and Google Scholar with the search string: (“ecosystem” or “carbon” or “methane”) and (“thaw slump” or “abrupt thaw” or “hillslope thermokarst”). By combining our field data, this study compiled a total of 291 SOCC measurements, 1,448 Reco measurements, and 414 CH_4_ release data from RTSs across the Tibetan Plateau. All field monitoring data covered the period from 2010 to 2023 (Supplementary Table 1).

### Environmental variable data

Prior to machine learning modeling, spatial filtering was applied to the initial RTS inventory to mitigate spatial autocorrelation and sampling bias. ENMTools was employed to remove redundant records by retaining solely one RTS occurrence per grid cell. This process reduced overfitting and improved model generalizability by more precisely capturing the relationship between RTS occurrence and environmental conditions. The annual counts of RTS incidents used in the model for 2016-2022, both before and after spatial filtering are presented in Supplementary Table 3. To simulate the distribution of RTS susceptibility, we collected 14 variables from global geospatial datasets (Supplementary Table 4). They included climate variables (mean annual precipitation (mm), mean annual ground temperature (°C) and mean annual air temperature (°C)), permafrost variables (ground ice (%) and active layer thickness (cm)), topographic variables (elevation (m), slope (°), aspect (°), Terrain Ruggedness Index, and relief degree of land surface), human footprint variable (Human Influence Index), and three soil variables from the Harmonized World Soil Database (HWSD, v1.2) such as sand fraction (% wt.), silt fraction (% wt.), and clay fraction (% wt.). Using stepwise regression, we addressed multicollinearity by removing predictor variables with high intercorrelation and retained the most representative variables for RTS susceptibility. Finally, we selected 10 environmental variables, including topography (elevation, slope, aspect), climate (mean annual precipitation, mean annual air temperature), permafrost conditions (active layer thickness, ground ice content), soil properties (silt, sand content), and human activity (Human Influence Index). Pearson correlation coefficients indicated no strong correlation among these variables (Supplementary Fig. 13). Detailed information on the environmental variables and their sources is provided in Supplementary Table 4. All geospatial data were resampled to a uniform spatial 1 × 1 km^2^ resolution and clipped to the study area extent^61^.

### Machine learning models and uncertainty analysis

Machine learning algorithms have advanced rapidly in the dynamic modeling of permafrost landforms^59–62^. They have been employed to assess the susceptibility of various hazards^62,63^, including the RTS dynamics at both local and regional scales^64–68^. In this study, we first evaluated nine individual models for RTS susceptibility mapping, including Random Forest (RF), Generalized Linear Model (GLM), Generalized Additive Model (GAM), Generalized Boosted Regression Model (GBM), Classification and Regression Tree Analysis (CTA), Artificial Neural Network (ANN), Flexible Discriminant Analysis (FDA), Multivariate Adaptive Regression Splines (MARS), and Maximum Entropy Model (MaxEnt). Model performance was evaluated using three metrics: the area under the receiver operating characteristic curve (AUC), Cohen’s kappa index (Kappa), and the True Skill Statistics (TSS). Based on the evaluation results, the four best-performing individual models (RF, GLM, CTA, and GBM) were selected to construct an ensemble model. We then compared the predictive performance of the nine individual models with that of the ensemble model. Among all models, RF exhibited the best overall performance (AUC = 0.963; KAPPA = 0.822; TSS = 0.823; Supplementary Table 5). Therefore, RF was selected as the optimal model for subsequent RTS susceptibility simulations. Each forest ensemble comprised 50 decision trees, implemented with the default *mtry* parameter and a terminal node size of 5. Model calibration was conducted over five evaluation runs, with 75% of samples assigned to model training and the remaining 25% reserved for validation. The final RTS susceptibility map was generated by averaging predictions across all RF realizations.

RTS susceptibility was spatially projected using the BIOMOD_Projection function in the *biomod2* package, which applies the trained RF model to the environmental raster layers to predict the probability of RTS occurrence for each grid cell across the plateau. The resulting raster depicted RTS susceptibility as a relative probability index ranging from 0 to 1. Following an established classification scheme^67^, RTS susceptibility was divided into five levels: very low (0–0.2), low (0.2–0.4), moderate (0.4–0.6), high (0.6–0.8), and very high (0.8–1.0). Grid cells with a probability index <0.2 were defined as control areas, whereas those with an index ≥ 0.2 were classified as RTS-influenced areas.

Predictive uncertainty was quantified based on the distribution of predictions from individual decision trees. Specifically, predictions from all trees within each fitted RF model were extracted to produce tree-level susceptibility rasters across the plateau. As the workflow integrated repeated pseudo-absence generation (10 replicates), repeated data partitioning (5 runs), and 50 trees per RF model, each grid cell received up to 2500 individual tree-level predictions. For each grid cell, uncertainty was quantified as the standard deviation (SD) of all tree-level predictions; higher SD values indicated greater divergence among predictions, corresponding to higher local uncertainty in RTS susceptibility mapping. We further calculated the coefficient of variation (CV; SD normalized by the mean prediction) to characterize relative predictive variability. The SD represents the absolute predictive uncertainty for each grid cell, while the CV reflects the relative stability of model predictions, with higher CV values indicating greater relative uncertainty in RTS susceptibility mapping (Supplementary Fig. 9).

### Drivers of RTS susceptibility

To quantify the effects of environmental variables on RTS susceptibility, we conducted a two-step analytical framework, specifically, combining the SHapley Additive exPlanations (SHAP) algorithm and sensitivity analysis to disentangle their respective contributions and spatial heterogeneity. The SHAP algorithm utilizes Shapley values to quantitatively assess the marginal contribution of each feature to model outputs^69^, and was employed herein to holistically quantify the overall contribution of each environmental variable to RTS susceptibility. The sensitivity analysis was designed to identify the dominant driving factors of RTS susceptibility at the pixel scale (1 × 1 km²), thereby capturing fine-grained spatial variations in the importance of influencing factors.

Specifically, we applied the SHAP algorithm using the Python package “shap” to quantify the relative influence of various environmental variables on RTS susceptibility across the entire study area. Further, based on our final random forest (RF) model, we performed a sensitivity analysis to identify primary drivers of RTS susceptibility in the permafrost regions^70^. This sensitivity analysis proceeded: first, we predicted RTS susceptibility for each 1 × 1 km² pixel using the full set of environmental variables; second, we quantified the relative sensitivity of RTS susceptibility to each variable by comparing the performance of the full RF model with that of “reduced models”, each excluding one environmental variable at a time. For each pixel, the dominant driver was defined as the variable with the highest sensitivity value (≥0.2). If no single variable met this threshold, the pixel was classified as being influenced by “co-drivers”.

### Changes of RTS susceptibility

We trained the RF model using the RTS observations from 2016 to 2022. To ensure accurate temporal and spatial alignment, observations in each year was matched with its corresponding environmental variables, which can enhance the association between RTSs and predictors, thereby improving model’s performance. Using the trained model, we substituted the environmental variables with year-specific values to predict RTS susceptibility on a raster-by-raster basis for each year. Climate data during January 1901 to December 2023 were derived from publicly available dataset, which were spatially downscaled from CRU TS v4.02 and WorldClim datasets^71,72^. To predict the changes in RTS susceptibility, we applied the trained model while keeping all other environmental variables unchanged and updating only the climate variables and active layer thickness for the corresponding period. Future climate inputs, including mean monthly air temperature and precipitation, were derived from an ensemble of three General Circulation Models: EC-Earth3, GFDL-ESM4, and MRI-ESM2-0 from the Coupled Model Intercomparison Project Phase Six (CMIP6)^73,74^. Active layer thickness data were obtained from published study^75^, and the SSP2-4.5 and SSP 5-8.5 scenarios were selected to ensure consistency with the climate projections. SSP2-4.5 represents a scenario with intermediate societal vulnerability coupled with an intermediate radiative forcing level, whereas SSP5-8.5 features high societal vulnerability and a high radiative forcing level. By adopting these two representative scenarios, this study encompasses a wide range of potential future climate policy outcomes.

To analyze regional differences in the driving factors and susceptibility distribution of RTSs, the study area was divided into seven regions based on the Global Terrestrial Network for Glaciers (GTN-G) (Supplementary Table 4). Minor sub-regions were merged to better reflect the capture spatial heterogeneity. Specifically, the original Hengduan Shan and Southeast Tibet regions were merged into a single “Southeast” region. West Kunlun and East Kunlun regions were merged into a single “Kunlun Mountains” region. West Himalaya and Central Himalaya regions were merged into a single “Himalaya” region.

### Estimation of carbon release from RTSs

We quantified the total regional carbon release from three vegetation types by multiplying their mean carbon release rates (based on monitoring data) by the area of RTS susceptibility^76^, where areas with high and very high susceptibility levels were defined as the RTS-affected region. An area density ratio was used to represent the actual RTS-affected area within each vegetation type. Potential overestimation was corrected using the mean RTS area density for each vegetation type over the 2016–2022 period (Eq. 1; Supplementary Table 6). Error propagation was used to estimate the uncertainty in carbon emissions, combining the standard errors of carbon release rates and area density estimates. The reliability of this susceptibility-based approach was validated by comparing its estimated annual carbon emissions with those derived from actual RTSs during the same period (Supplementary Fig. 10). The validated framework was subsequently applied to project future RTS-driven carbon emissions under the SSP2-4.5 scenario.

1 $${{{\rm{Area density}}}}=\frac{{{{{\rm{area}}}}}_{{{{\rm{RTS}}}}}}{{{{{\rm{area}}}}}_{{{{\rm{sus}}}}}}$$

where Area density represents the ratio of susceptibility raster to the actual RTS area (m^2^/km^2^) for each level (low, moderate, high, and very high), area_RTS_ and area_sus_ refer total area of RTSs (m^2^) and susceptibility under each level (km^2^), respectively.

### Reporting summary

Further information on research design is available in the Nature Portfolio Reporting Summary linked to this article.

## Supplementary information

Supplementary Information

Description of Additional Supplementary Files

Supplementary Dataset 1

Reporting Summary

Transparent Peer Review file

## Data Availability

Source Data are provided with this paper. All data supporting the findings are available in the Figshare data repository (https://figshare.com/s/35398fbf2b21bacbe542) and Supplementary Information.

## Code availability

For data analysis and organization, we used R version 4.2.2 (available at https://www.R-project.org/), resampling and cropping, etc. were done using python version 3.11 (available at https://www.python.org/), model construction, importance ranking and visualization were done using R statistical software. Statistical charts were created using Origin 2024 (available at https://www.originlab.com/). GIS processing and presentation was done using ArcMap 10.8. (available at https://www.esri.com/en-us/arcgis/products/arcgis-desktop/overview). The supporting code is provided at Figshare (https://figshare.com/s/a09b8262eeed2b34c237).
