# Supplementary material for: Abrupt permafrost thaw drives exceptional carbon release across the Tibetan Plateau
Source: Nat Commun. 2026 Jun 27;17:8050. doi: 10.1038/s41467-026-74850-y (PMC13454460; doi:10.1038/s41467-026-74850-y)
Supplement: Supplementary file 1 [file 41467_2026_74850_MOESM1_ESM.pdf]

Supplementary Information for:

Abrupt permafrost thaw drives exceptional carbon release across the Tibetan Plateau

Supplementary Figures 1 to 13

Supplementary Tables 1 to 6

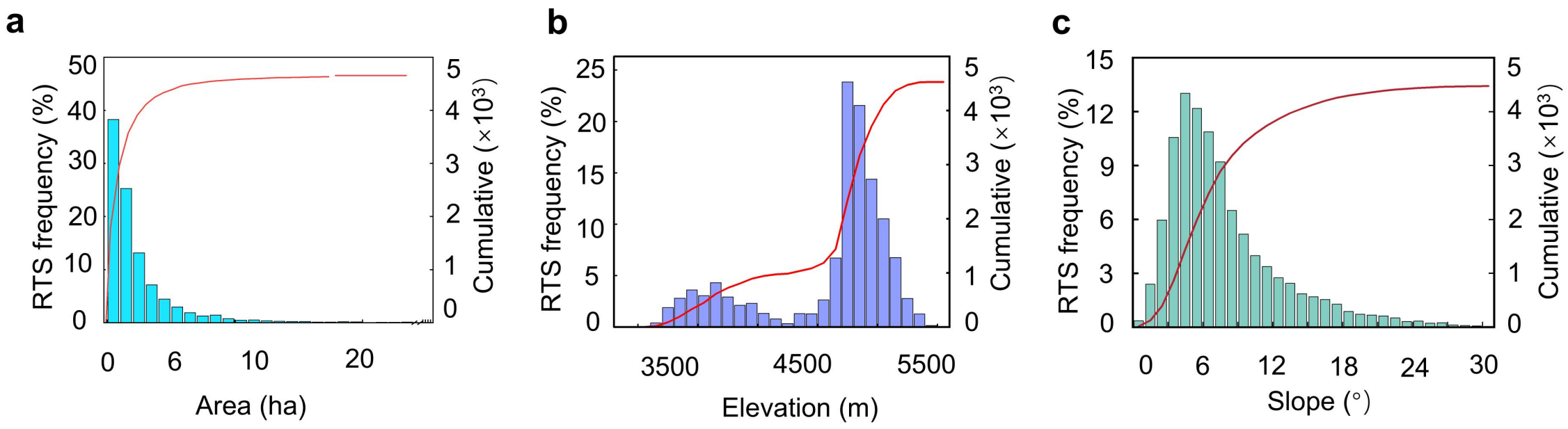

Supplementary Figure 1. Distribution of field-observed retrogressive thaw slumps (RTSs) across the Tibetan Plateau. (a) Area frequency distribution of RTSs. **(b)** Elevation frequency distribution of RTSs. **(c)** Slope frequency distribution of RTSs.

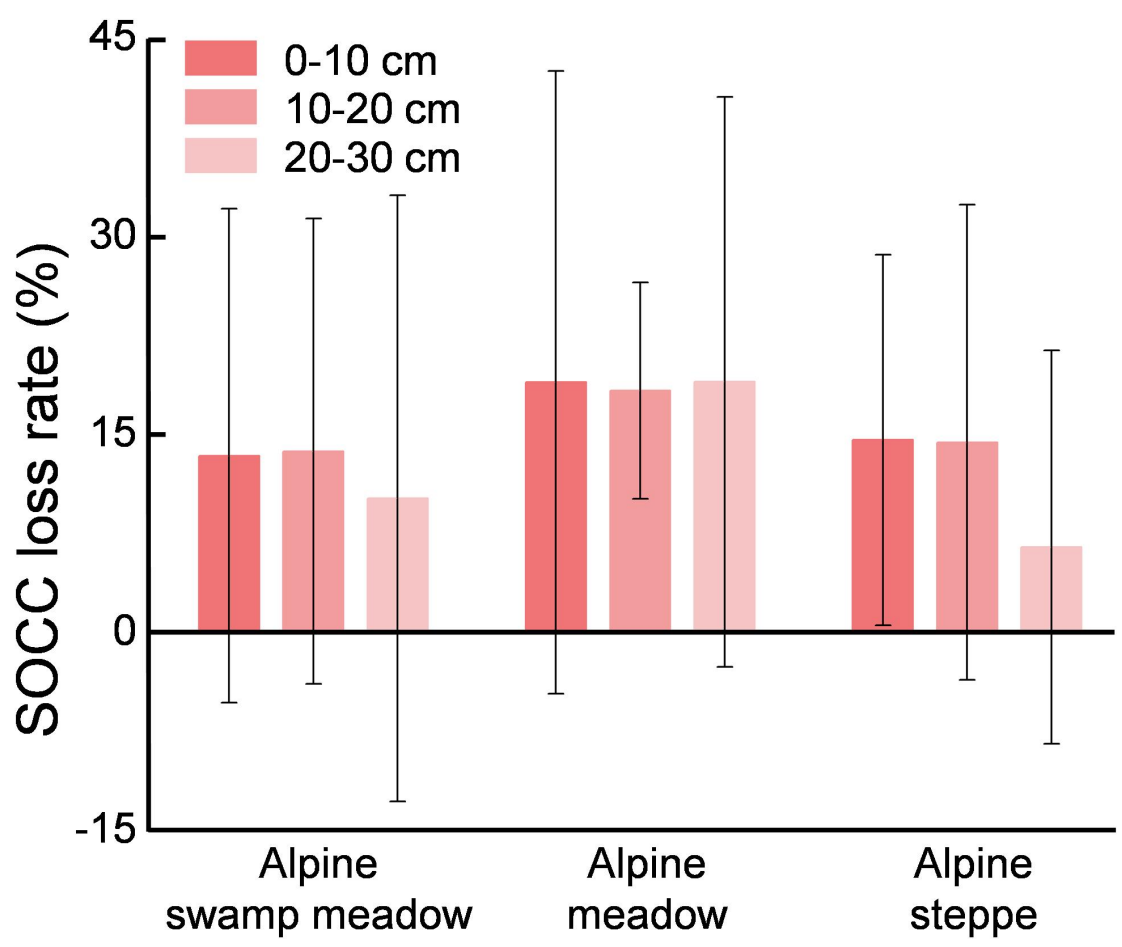

Supplementary Figure 2. Vertical variations in soil organic carbon content (SOCC) loss caused by RTSs across three vegetation types. Error bars represent standard deviation.

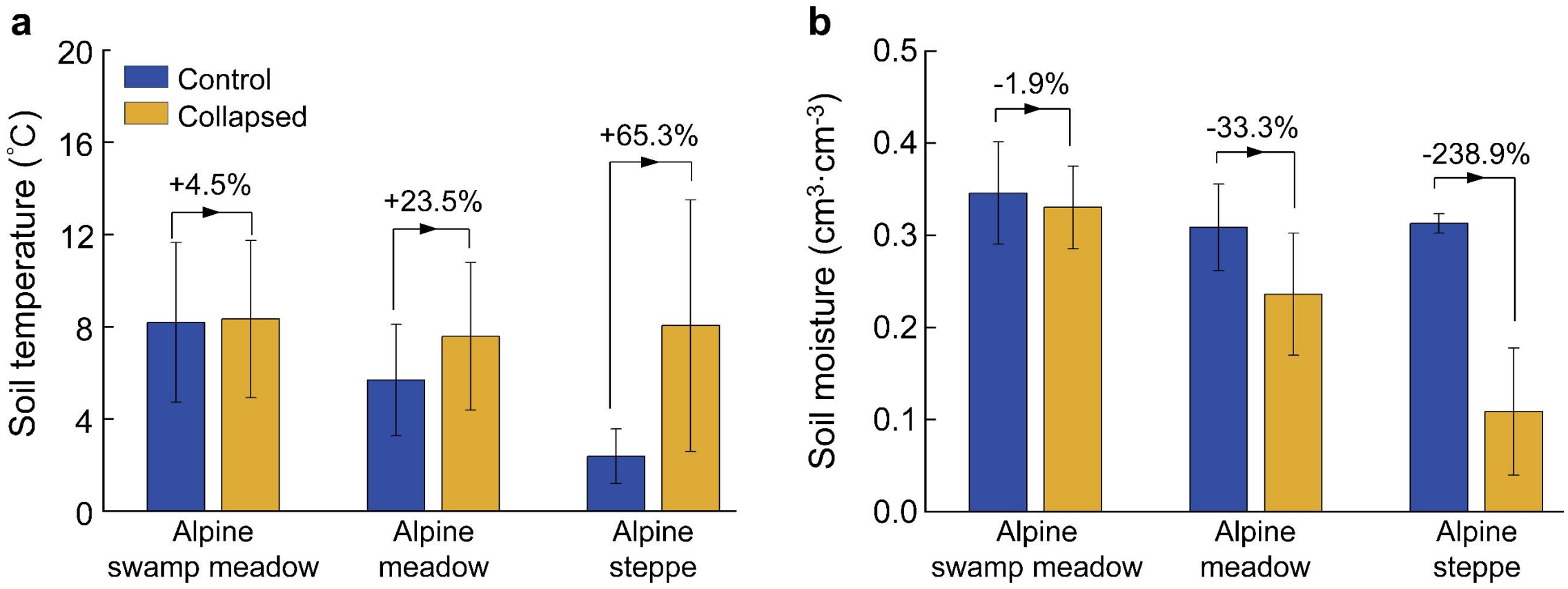

Supplementary Figure 3. Changes in soil temperature (a) and moisture (b) at the 0–10 cm depth in the control and collapsed areas of RTSs under different vegetation types during the growing seasons. Error bars represent standard deviation.

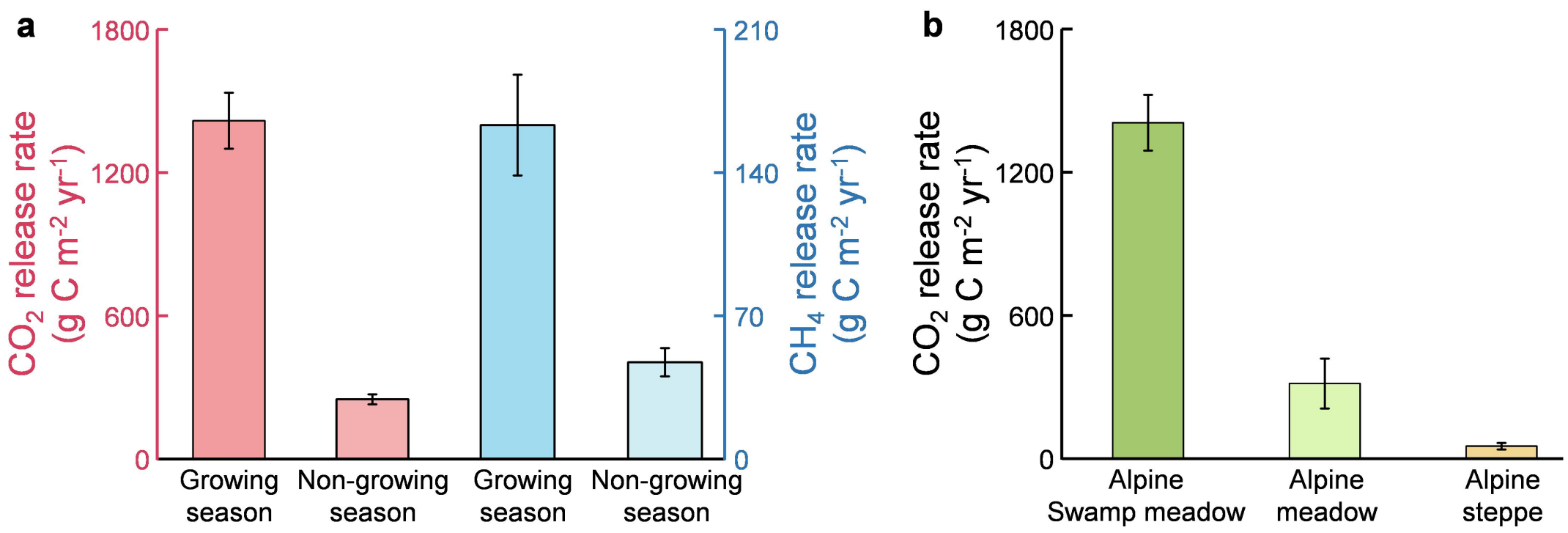

Supplementary Figure 4. Variations in carbon release rates induced by RTSs across seasons and three vegetation types. (a) Seasonal mean carbon release rates in alpine swamp meadow. **(b)** Mean ecosystem respiration (Reco) across the three vegetation types during the growing season. Error bars represent standard errors.

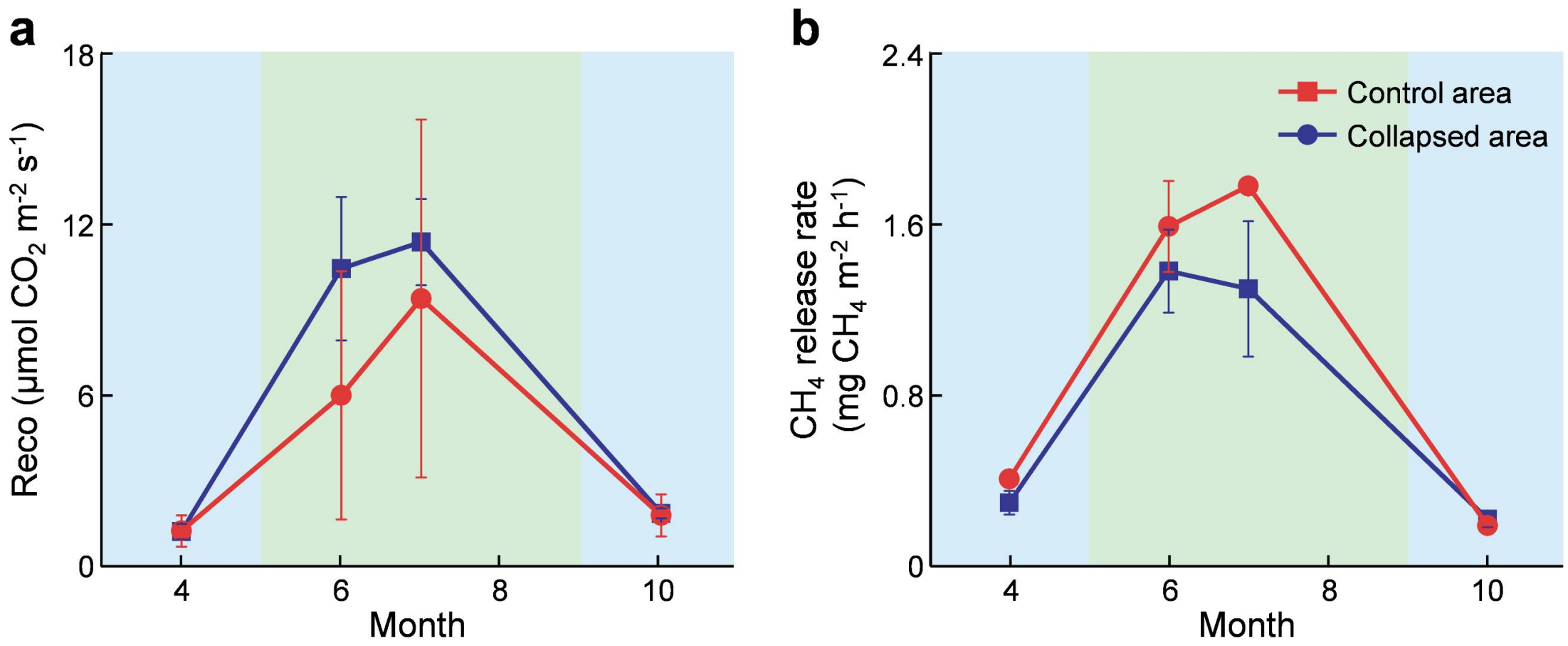

Supplementary Figure 5. Seasonal variations in carbon release from RTSs in alpine swamp meadow. (a) Seasonal pattern of ecosystem respiration (Reco). **(b)** Seasonal pattern of CH_4 release rate. Error bars represent standard deviation.

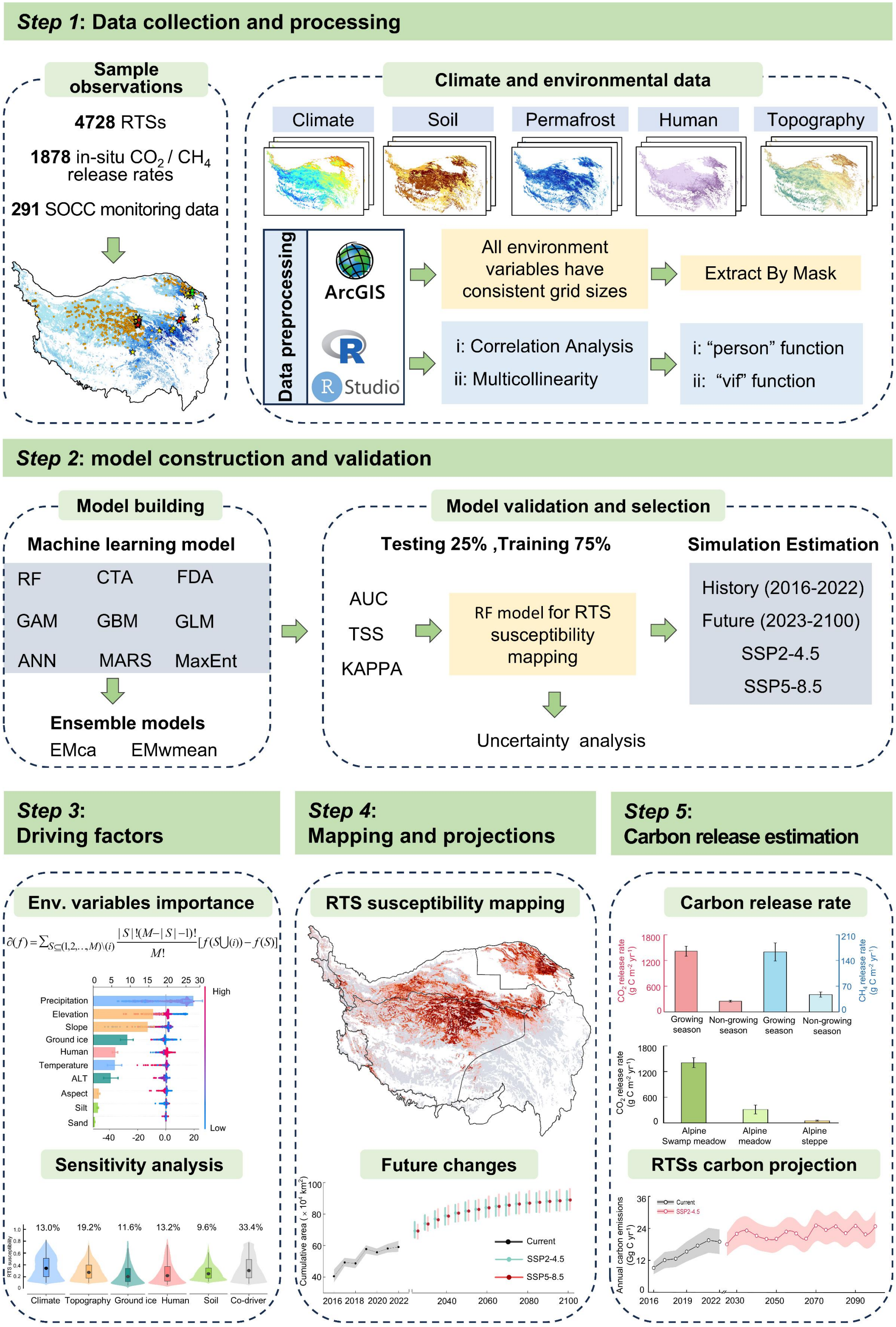

Supplementary Figure 6. The study flowchart to illustrate the changes of RTS susceptibility and carbon release on the Tibetan Plateau. It consists of five specific processes.

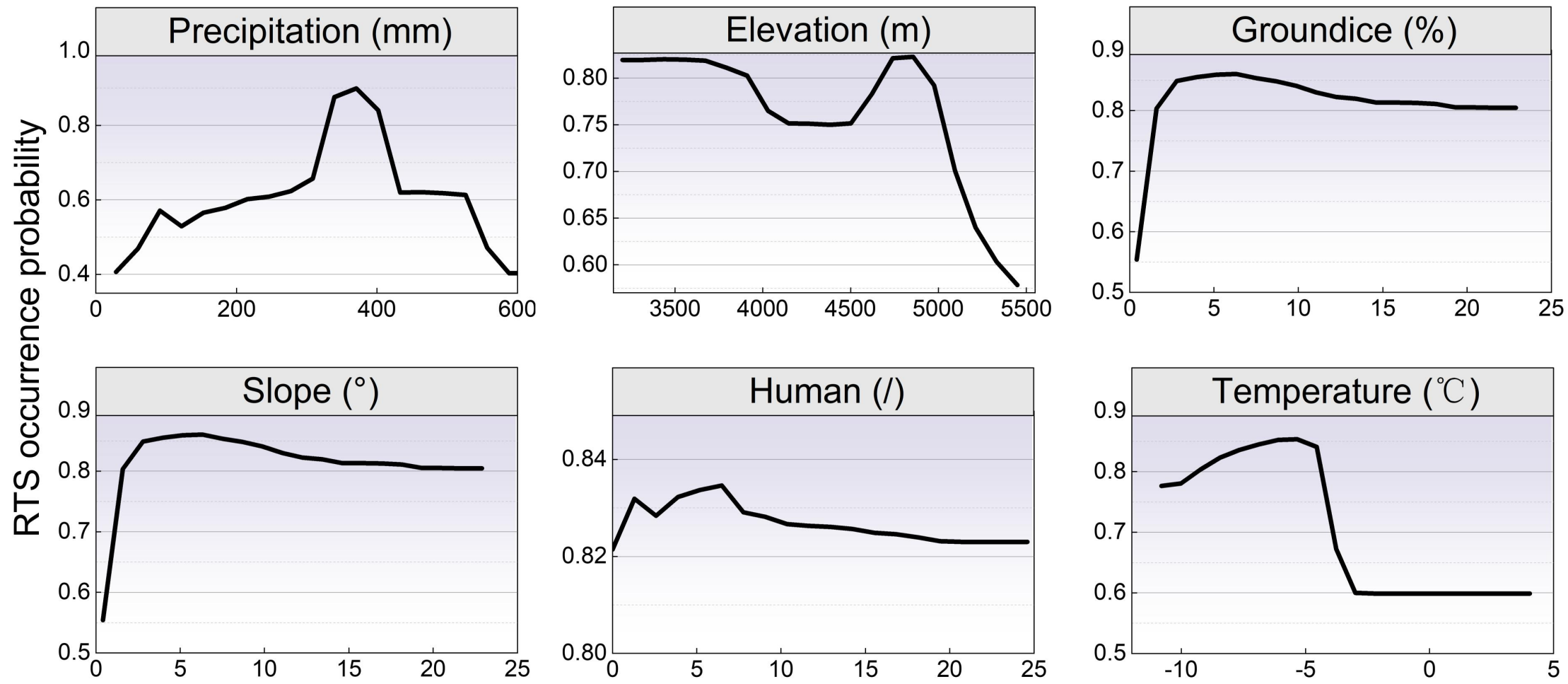

Supplementary Figure 7. Response curves of influencing factors on RTSs distribution including mean annual precipitation, elevation, ground ice content, slope, human footprint index, and mean annual air temperature.

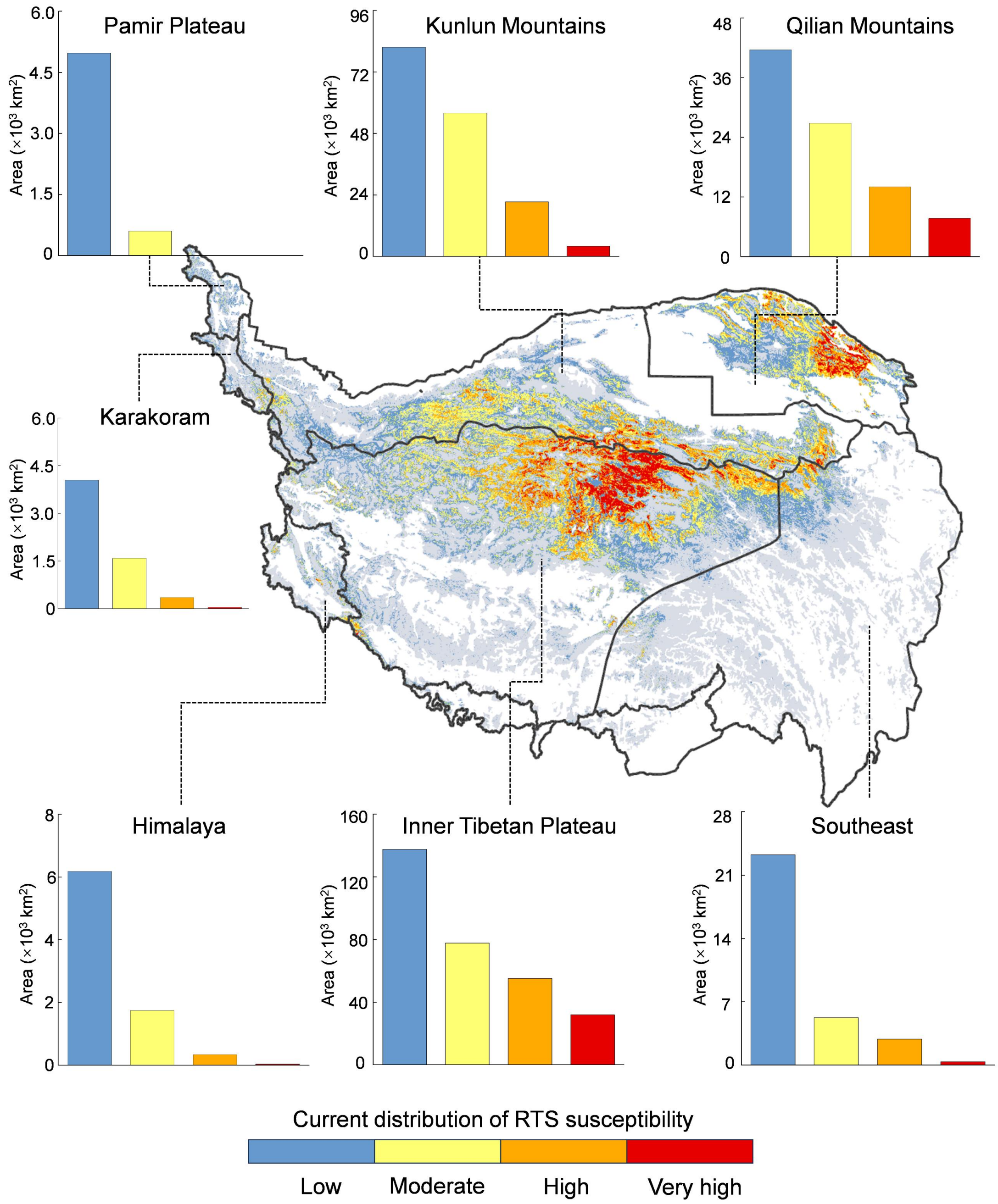

Supplementary Figure 8. Distribution of RTSs susceptibility across seven mountain regions in the Tibetan Plateau. Grey shading represents permafrost distribution, derived from Obu et al. (2019)[1]. Bar charts show the total area of each susceptibility level within each mountainous region.

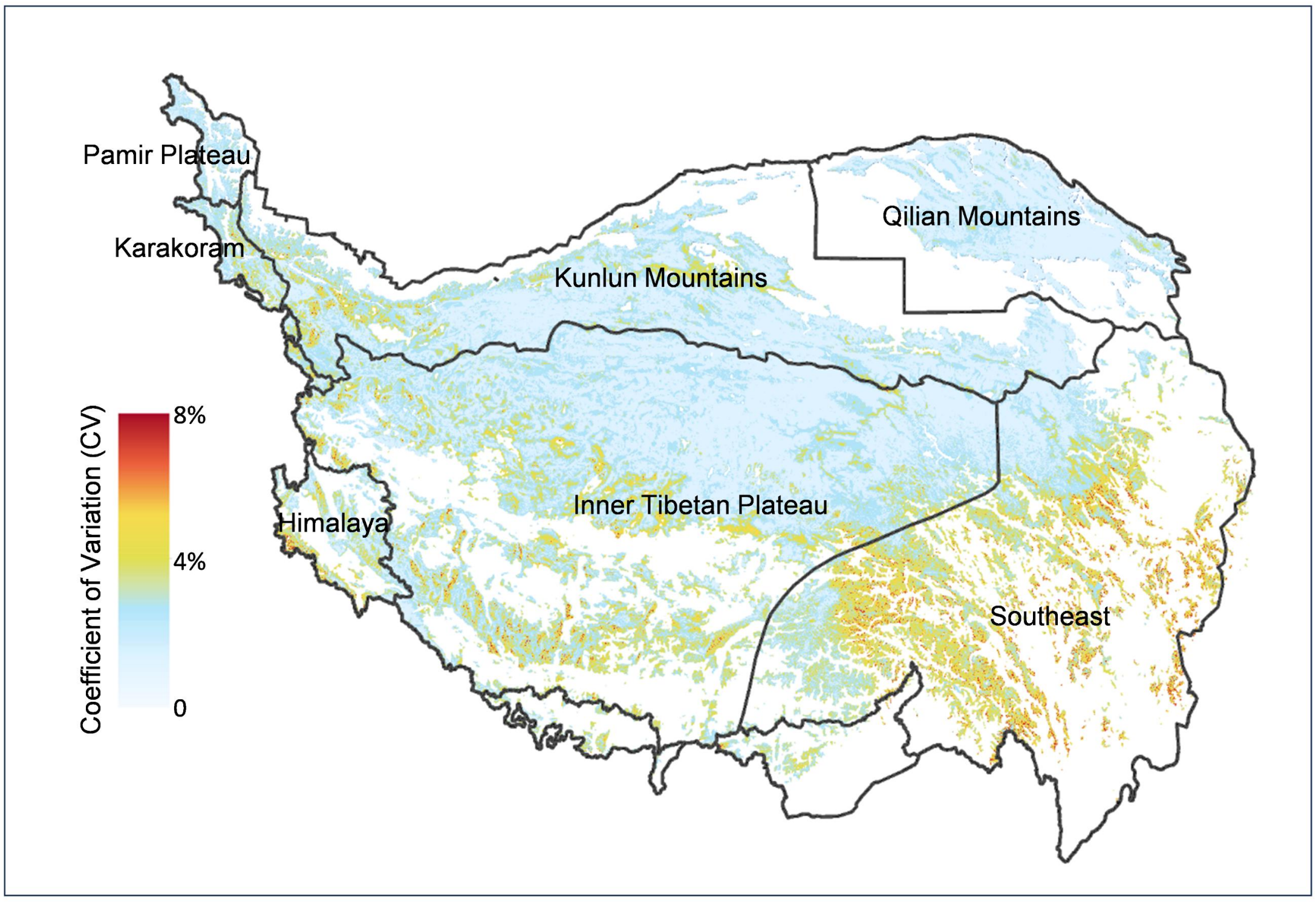

Supplementary Figure 9. Spatial uncertainty of RTSs susceptibility with coefficient of variation.

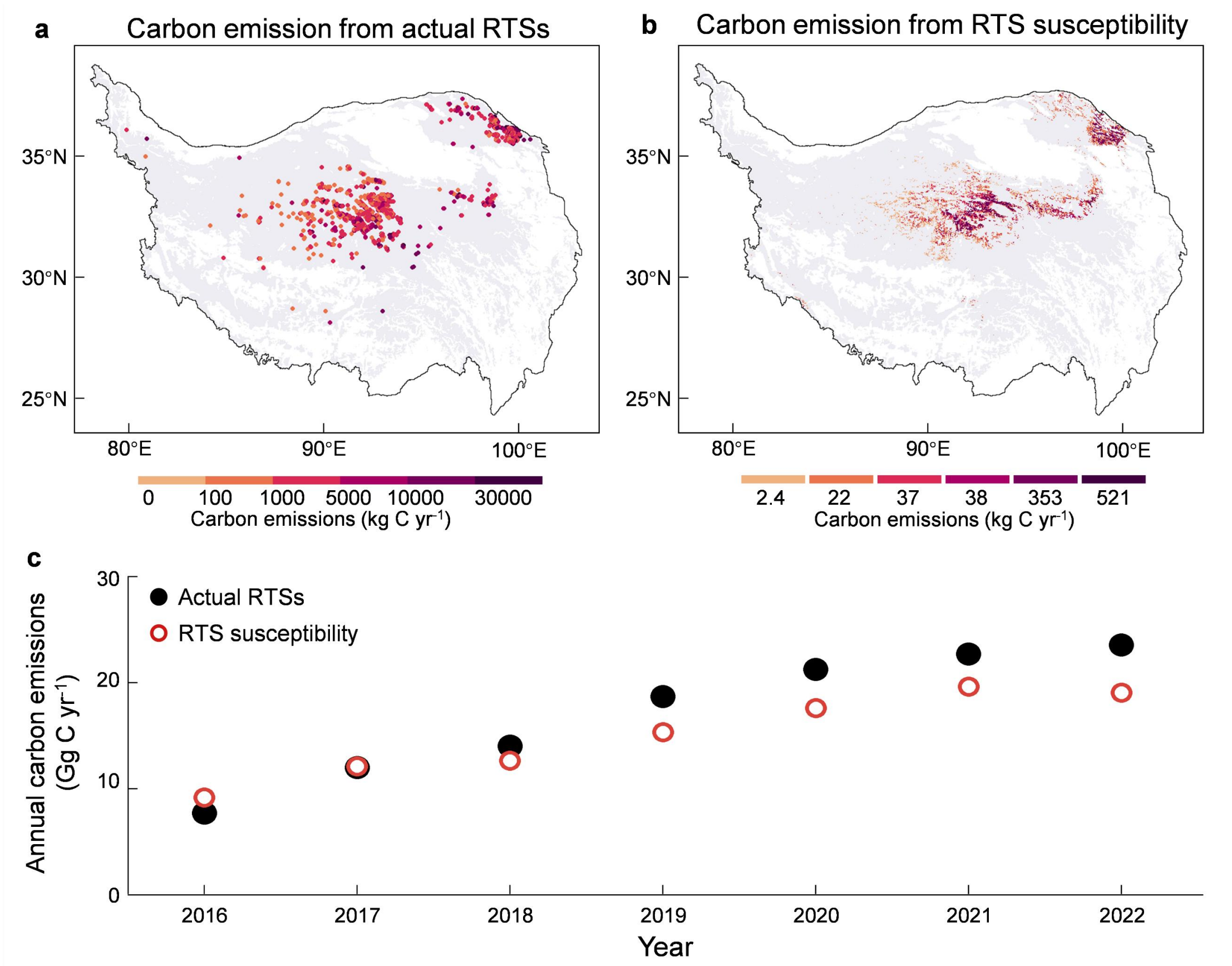

Supplementary Figure 10. Validation of carbon emissions from actual RTSs and RTS susceptibility. (**a**) Spatial distribution of carbon emissions from actual RTSs in 2022. (**b**) Spatial distribution of carbon emissions from RTS susceptibility in 2022. (**c**) Validation of annual carbon emissions from 2016 to 2022.

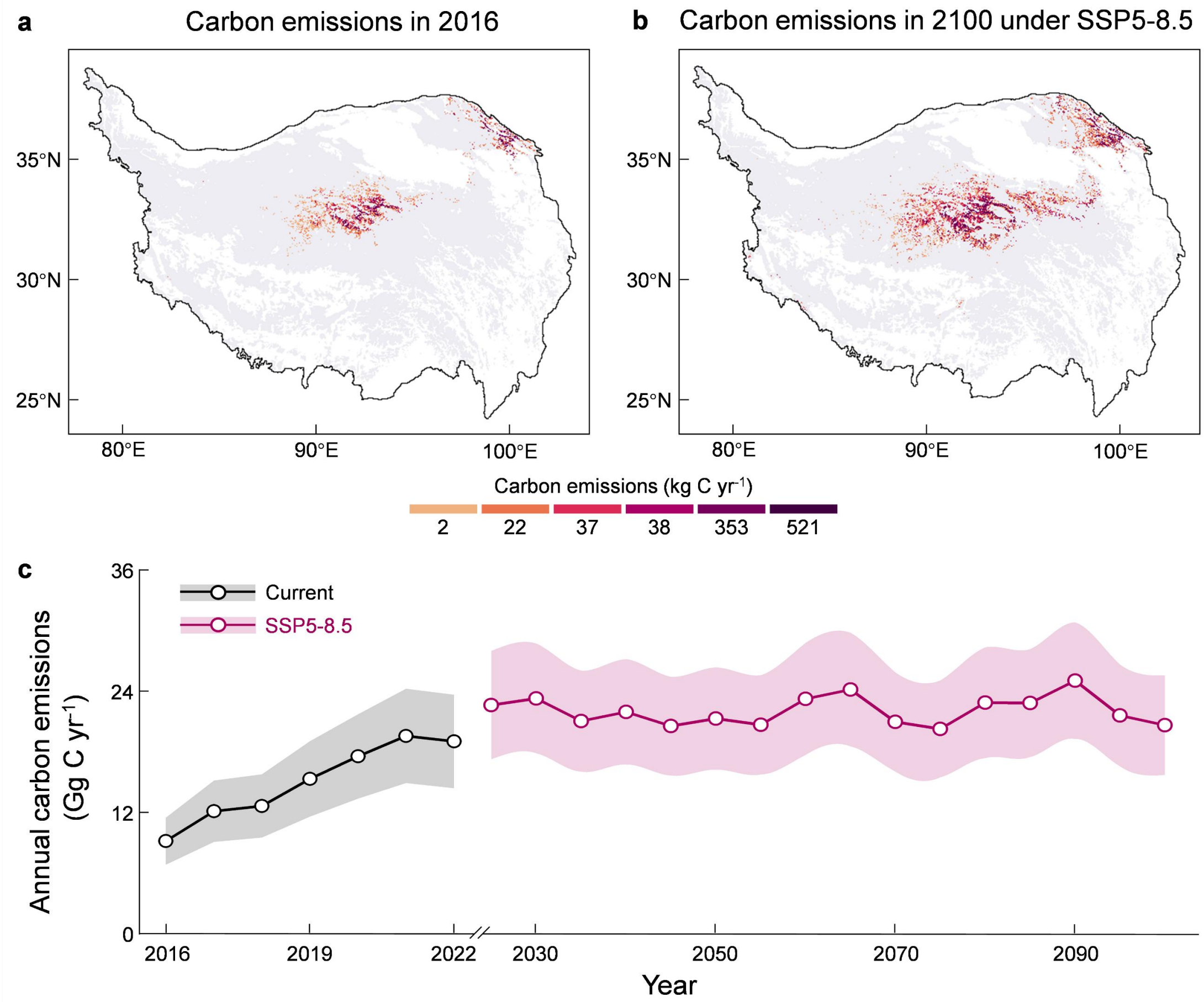

Supplementary Figure 11. Carbon emissions from RTS susceptibility areas during 2016-2100. (a-b) Spatial distribution of carbon emissions from RTS susceptibility in 2016 **(a)** and 2100 **(b)** under the SSP5-8.5 scenario. **(c)** Projected annual total carbon emissions from RTS susceptibility under the SSP5-8.5 scenario. The shaded areas denote the uncertainty calculated via error propagation.

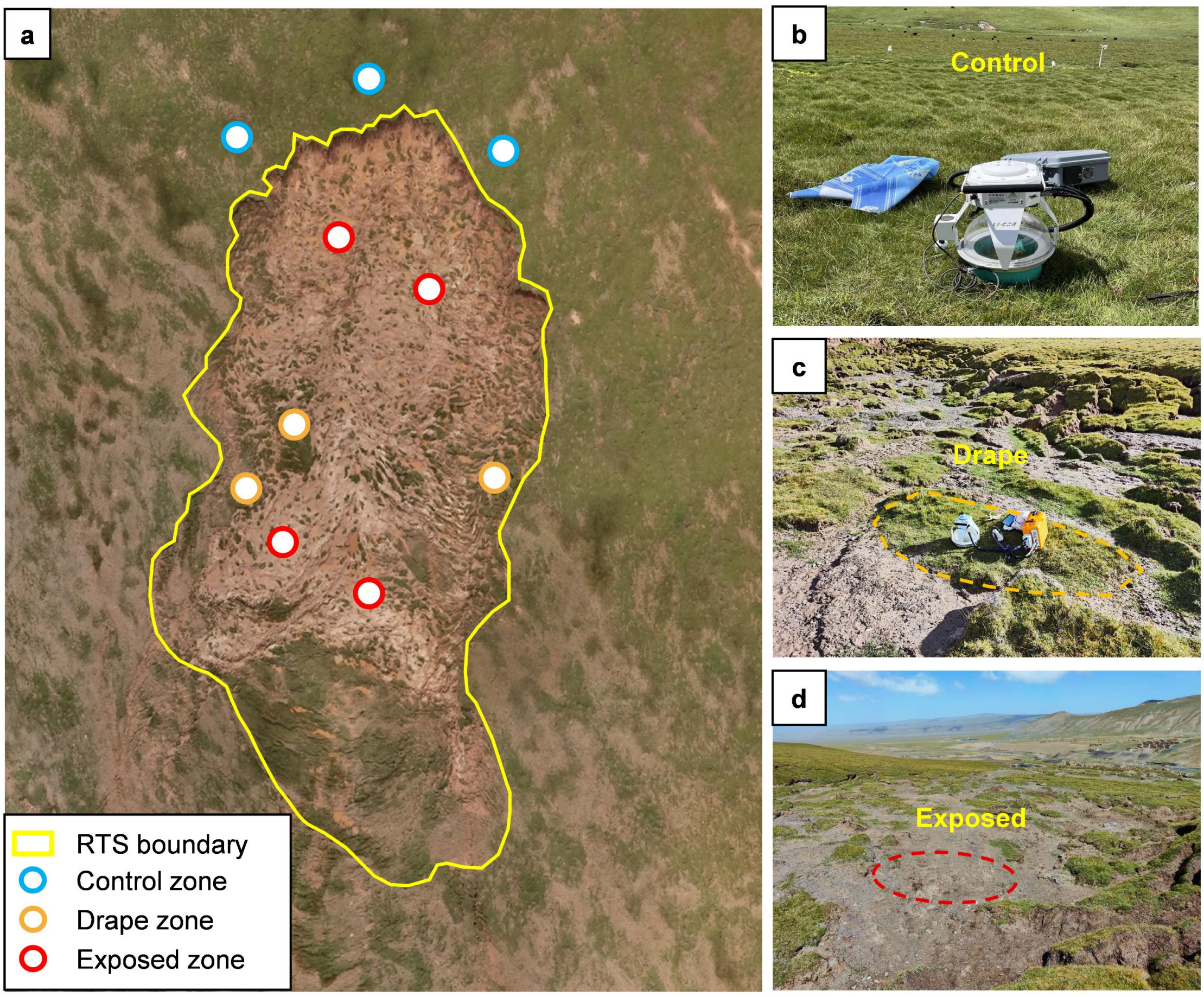

Supplementary Figure 12. In-situ carbon monitoring sites within a representative retrogressive thaw slump RTS. (a) Distribution of carbon monitoring sites within the RTS. The base map is an unmanned aerial vehicle (UAV) orthomosaic image provided by author Liu Lin in August 2023. The RTS is schematically divided into three functional zones: control zone, drape zone, and exposed zone, illustrating the typical layout of sampling points for gas flux and soil property measurements. **(b-d)** Field photos taken at the RTS (38.00°N, 100.91°E) in August 2023. **(b)** Control zone; **(c)** Drape zone; **(d)** Exposed zone (without vegetation cover).

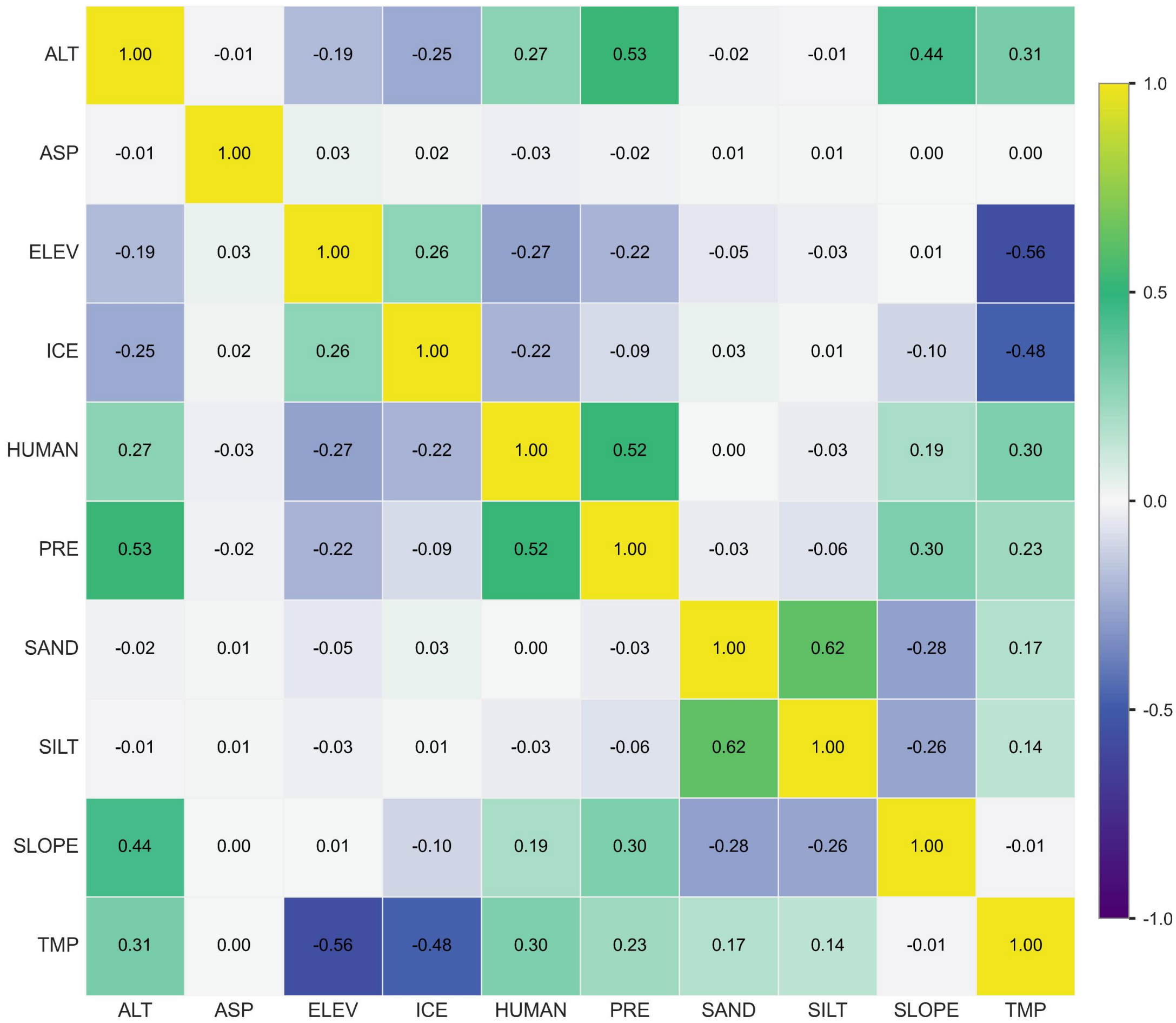

Supplementary Figure 13. Correlation heatmap of environmental factors for RTSs susceptibility modeling.

Supplementary Table 1. Carbon release measurements compiled from RTS studies on the Tibetan Plateau.

References	Number of RTS sites	Carbon measurement type	Monitoring year	Control measurements	Collapsed measurements
Xu et al., 2018[2]	1	SOC	2015	3	3
Liu et al., 2022[3]	6	SOC	2014, 2020	40	55
Mu et al., 2020[4]	6	SOC	2016	6	12
Wang et al., 2023[5]	1	SOC		3	6
Hu et al., 2023[6]	1	SOC	2019	3	6
Zhou et al., 2023[7]	1	SOC	2021	18	30
Ding et al., 2023[8]	5	SOC	2020-2021	5	15
Yang et al., 2018[9]	1	CH_4	2016	2	6
Wang et al., 2024[10]	1	CO_2	2019, 2020	27	27
Yang et al., 2021[11]	1	CO_2	2017–2019	22	66
Wang et al., 2022[12]	1	CO_2	2018, 2019	29	87
Mu et al., 2017[13]	1	CO_2	2014-2016	12	24
Mu et al., 2017[13]	1	CH_4	2016	3	6
Mu et al., 2023[14]	7	SOC	2018	7	12
Mu et al., 2023[14]	7	CO_2	2018, 2020, 2021	20	39
Mu et al., 2023[14]	7	CH_4	2018, 2019, 2021	5	10
This study	37	SOC	2021, 2023	31	36
This study	17	CO_2	2021–2023	425	670
This study	4	CH_4	2021–2023	133	249

***Soil organic carbon (SOC). Control and collapsed measurements indicate number of field observations.**

Supplementary Table 2. Annual carbon emissions from RTS susceptibility in 2016 on the Tibetan Plateau.

Vegetation types	Carbon emissions (Gg C yr^{-1})	Percentage of total (%)
Alpine swamp meadow	0.4 ± 0.04	4.2
Alpine meadow	8.3 ± 2.3	90.7
Alpine steppe	0.5 ± 0.1	5.1

Supplementary Table 3. The annual counts of RTSs incidents used in machine learning model during 2016-2022 before and after spatial filtering.

Year	Counts of RTS incidents before spatial filtering	Counts of RTS incidents after spatial filtering
2016	2,165	1,482
2017	3,424	2,129
2018	3,657	2,262
2019	4,495	2,721
2020	4,553	2,781
2021	4,706	2,864
2022	4,728	2,870

Supplementary Table 4. Information of climate and environmental data sources.

Environmental data	Factor	Resolution	Source	Source Website
Topography	Elevation, Slope, Aspect	1km	Amatulli et al., 2018[15]	https://www.nature.com/articles/sdata201840
Soil type	Silt, Sand	1km	Harmonized world soil database (version 1.2)	Harmonized world soil database v1.2 | FAO SOILS PORTAL | Food and Agriculture Organization of the United Nations
Permafrost	Ground ice	1km	Zou et al., 2024[16]	https://doi.org/10.11888/Cryos.tpdc.300748
	Active layer thickness	1km	Jin et al., 2024[17]	https://doi.org/10.1016/j.gloenvcha.2023.102791
Qinghai Tibetan Plateau boundary		1km	Zou et al., 2017[18]	https://doi.org/10.11888/Geocry.tpdc.270468
Human activity	Human footprint Index	1km	Last of the Wild, v2	https://sedac.ciesin.columbia.edu/
Climatic data	Mean annual precipitation<br>Mean annual air temperature	1km	Peng et al., 2024[19-23]	https://data.tpdc.ac.cn/zh-hans/data/7f0fce77-2cba-4bdc-ab02-b2d7c69e8e4b
Randolph Glacier Inventory 7.0 data			Global Terrestrial Network for Glaciers (GTN-G)	https://www.gtn-g.ch/data_catalogue_glacreg/.
Carbon storage data		1km	Wang et al., 2021[24]	https://doi.org/10.5281/zenodo.4293454

Supplementary Table 5. Accuracy evaluation of machine learning models.

Model	AUC			KAPPA			TSS		
	**MEAN**	**SD**	**CV**	**MEAN**	**SD**	**CV**	**MEAN**	**SD**	**CV**
RF	0.963	0.052	0.061	0.822	0.134	0.015	0.823	0.285	0.034
EM	0.960	0.051	0.053	0.804	0.151	0.018	0.804	0.143	0.018
ANN	0.883	0.023	0.222	0.644	0.394	0.060	0.645	0.392	0.061
CTA	0.905	0.081	0.091	0.719	0.193	0.027	0.718	0.205	0.027
FDA	0.903	0.062	0.071	0.650	0.145	0.021	0.652	0.145	0.022
GAM	0.923	0.071	0.070	0.689	0.133	0.019	0.690	0.134	0.018
GBM	0.924	0.075	0.080	0.688	0.202	0.028	0.689	0.192	0.027
GLM	0.895	0.081	0.090	0.646	0.131	0.021	0.645	0.144	0.022
MARS	0.910	0.072	0.070	0.656	0.113	0.017	0.658	0.126	0.018
MAXENT	0.890	0.112	0.123	0.618	0.184	0.029	0.620	0.181	0.029

***Random Forest (RF), ensemble model (EM), Artificial Neural Network (ANN), Classification and Regression Tree Analysis (CTA), Flexible Discriminant Analysis (FDA), Generalized Additive Model (GAM), Generalized Boosted Regression Model (GBM), Generalized Linear Model (GLM), Multivariate Adaptive Regression Splines (MARS), and Maximum Entropy Model (MaxEnt)**

Supplementary Table 6. Multi-year mean area density of RTS susceptibility at high and very high levels during 2016-2022. Values are presented as mean ± standard error (SE).

**Vegetation type**	**High (m^2/km^2)**	**Very high (m^2/km^2)**
Alpine swamp meadow	10.8 ± 4.4	294.8 ± 71.9
Alpine meadow	55.8 ± 9.4	882.6 ± 171.6
Alpine steppe	33.5 ± 5.2	511.4 ± 99.7

Supplementary References

1. Obu, J. et al. Northern Hemisphere permafrost map based on TTOP modelling for 2000–2016 at 1 km^2 scale. *Earth Sci. Rev*. 193, 299-316 (2019).
2. Xu, H. et al. Soil enzyme response to permafrost collapse in the Northern Qinghai-Tibetan Plateau. *Ecol. Indic.* 85, 585-593 (2018).
3. Liu, F. et al. Divergent changes in particulate and mineral-associated organic carbon upon permafrost thaw. *Nat. Commun.* 13, 5073 (2022).
4. Mu, C. et al. Permafrost degradation enhances the risk of mercury release on Qinghai-Tibetan Plateau. *Sci. Total Environ.* 708, 135127 (2020).
5. Wang, L. et al. Effects of thaw slump on soil bacterial communities on the Qinghai-Tibet Plateau. *Catena* 232, 107342 (2023).
6. Hu, X., & Yang, Z. Changes in Soil Microbial Community during Permafrost Collapse Process on the Northeast Qinghai-Tibetan Plateau, China. *J. Soil Sci. Plant Nutr.* 23, 4157-4167 (2023).
7. Zhou, W. et al. Dramatic Carbon Loss in a Permafrost Thaw Slump in the Tibetan Plateau is Dominated by the Loss of Microbial Necromass Carbon. *Environ. Sci. Technol.* 57, 6910-6921 (2023).
8. Ding, M., Li, X. & Jin, Z. Digital Mapping of Soil Organic Carbon Using UAV Images and Soil Properties in a Thermo-Erosion Gully on the Tibetan Plateau. *Remote Sens.* 15, 1628 (2023).
9. Yang, G. et al. Changes in Methane Flux along a Permafrost Thaw Sequence on the Tibetan Plateau. *Environ. Sci. Technol.* 52, 1244-1252 (2018).
10. Wang, G. et al. Enhanced response of soil respiration to experimental warming upon thermokarst formation. *Nat. Geosci.* 17, 532-538 (2024).
11. Yang, G. et al. Phosphorus rather than nitrogen regulates ecosystem carbon dynamics after permafrost thaw. *Glob. Chang Biol.* 27, 5818-5830 (2021).
12. Wang, G. et al. Divergent Trajectory of Soil Autotrophic and Heterotrophic Respiration upon Permafrost Thaw. *Environ. Sci. Technol.* 56, 10483-10493 (2022).
13. Mu, C. et al. Permafrost collapse shifts alpine tundra to a carbon source but reduces N_2O and CH_4 release on the northern Qinghai-Tibetan Plateau. *Geophys. Res. Lett.* 44, 8945-8952 (2017).
14. Mu, M. et al. Carbon loss and emissions within a permafrost collapse chronosequence. *Catena* 231, 107291 (2023).
15. Amatulli, G. et al. A suite of global, cross-scale topographic variables for environmental and biodiversity modeling. *Sci Data*, 5, 180040 (2018).
16. Zou, D. et al. Estimation of Permafrost Ground Ice to 10 m Depth on the Qinghai-Tibet Plateau. *Permafr and Periglac Process*, 35, 423-434 (2024).
17. Jin H. et al. The infrastructure cost of permafrost degradation for the northern hemisphere. Global Environ Chang. 84, 102791(2024).
18. Zou, D. et al. A new map of permafrost distribution on the Tibetan Plateau. *Cryosphere*, 11, 2527-2542 (2017).

19. Peng, S., Gang, C., Cao, Y. & Chen Y. Assessment of climate change trends over the Loess Plateau in China from 1901 to 2100. *Int J Climatol*, 38, 2250-2264 (2018).
20. Peng, S. et al. Spatiotemporal change and trend analysis of potential evapotranspiration over the Loess Plateau of China during 2011–2100. *Agric. For. Meteorol.* 233, 183-194 (2017).
21. Peng, S., Ding, Y., Liu, W. & Li, Z. 1 km monthly temperature and precipitation dataset for China from 1901 to 2017. *Earth Syst. Sci. Data* 11, 1931-1946 (2019).
22. Peng, S. 1 km multi-scenario and multi-model monthly precipitation data for China (2021-2100). National Tibetan Plateau / Third Pole Environment Data Center. https://doi.org/10.11866/db.loess.2021.002 (2025).
23. Peng, S. 1 km multi-scenario and multi-model monthly temperature data for China (2021-2100). National Tibetan Plateau / Third Pole Environment Data Center. https://doi.org/10.11866/db.loess.2021.003 (2025).
24. Wang, D. et al. A 1 km resolution soil organic carbon dataset for frozen ground in the Third Pole. *Earth Syst. Sci. Data* 13, 3453-3465 (2021).
