# Supplementary material for: Abrupt permafrost thaw drives exceptional carbon release across the Tibetan Plateau
Source: Nat Commun. 2026 Jun 27;17:8050. doi: 10.1038/s41467-026-74850-y (PMC13454460; doi:10.1038/s41467-026-74850-y)
Supplement: Supplementary file 2 [file 41467_2026_74850_MOESM2_ESM.pdf]

Description of Additional Supplementary Files

Supplementary Dataset 1. Soil organic carbon content (SOCC), CO_2 release rate, and CH_4 flux data from the retrogressive thaw slump (RTS) site.

This dataset contains four worksheets with measurements collected from the RTS site, including both original field data and literature-compiled data.

Worksheet 1: SOCC

This sheet includes soil organic carbon content (SOCC) data compiled from both this study and published literature. Each entry includes the following information:

- Longitude and latitude of the slump site
- Year and month of sampling
- Sampling depth
- SOCC
- Site type (control / drape / exposed)
- Vegetation type

Worksheet 2: SOCC_original

This sheet contains the original field measurements of soil organic carbon content (SOCC) collected by the authors. Each entry includes:

- Longitude and latitude of the slump site
- Year and month of sampling
- Sampling depth
- SOCC
- Site type (control / drape / exposed)
- Vegetation type

Worksheet 3: CO_2

This sheet includes ecosystem respiration data compiled from both this study and published literature. Each entry includes:

- Longitude and latitude of the slump site
- Year and month of sampling
- Ecosystem respiration rate (Reco)
- Site type (control / drape / exposed)

- Vegetation type

Worksheet 4: CH_4 flux

This sheet includes methane (CH_4) flux data compiled from both this study and published literature. Each entry includes:

- Longitude and latitude of the slump site
- Year and month of sampling
- CH_4
- Site type (control / drape / exposed)
- Vegetation type

File format: Microsoft Excel (.xlsx).
