# Supplementary material for: Abrupt permafrost thaw drives exceptional carbon release across the Tibetan Plateau
Source: Nat Commun. 2026 Jun 27;17:8050. doi: 10.1038/s41467-026-74850-y (PMC13454460; doi:10.1038/s41467-026-74850-y)
Supplement: Supplementary file 3 [file 41467_2026_74850_MOESM4_ESM.pdf]

nature portfolio

Corresponding author(s): Prof. Cuicui Mu

Last updated by author(s): May 9, 2026

Reporting Summary

Nature Portfolio wishes to improve the reproducibility of the work that we publish. This form provides structure for consistency and transparency in reporting. For further information on Nature Portfolio policies, see our Editorial Policies and the Editorial Policy Checklist.

Statistics

For all statistical analyses, confirm that the following items are present in the figure legend, table legend, main text, or Methods section.

n/a	Confirmed	
☐	☒	The exact sample size (*n*) for each experimental group/condition, given as a discrete number and unit of measurement
☐	☒	A statement on whether measurements were taken from distinct samples or whether the same sample was measured repeatedly
☒	☐	The statistical test(s) used AND whether they are one- or two-sided<br>*Only common tests should be described solely by name; describe more complex techniques in the Methods section.*
☒	☐	A description of all covariates tested
☐	☒	A description of any assumptions or corrections, such as tests of normality and adjustment for multiple comparisons
☐	☒	A full description of the statistical parameters including central tendency (e.g. means) or other basic estimates (e.g. regression coefficient) AND variation (e.g. standard deviation) or associated estimates of uncertainty (e.g. confidence intervals)
☐	☒	For null hypothesis testing, the test statistic (e.g. *F*, *t*, *r*) with confidence intervals, effect sizes, degrees of freedom and *P* value noted<br>*Give P values as exact values whenever suitable.*
☒	☐	For Bayesian analysis, information on the choice of priors and Markov chain Monte Carlo settings
☒	☐	For hierarchical and complex designs, identification of the appropriate level for tests and full reporting of outcomes
☒	☐	Estimates of effect sizes (e.g. Cohen's *d*, Pearson's *r*), indicating how they were calculated

Our web collection on statistics for biologists contains articles on many of the points above.

Software and code

Policy information about availability of computer code

Data collection	Data were obtained through field observations, published literature, direct extraction from text or tables, and by digitizing figures using WebPlotDigitizer 4.2.
Data analysis	Data analysis in this study were conducted at Microsoft Excel, Origin 2021, ArcGis 10.8, Python 3.11 and R 4.2.2.

For manuscripts utilizing custom algorithms or software that are central to the research but not yet described in published literature, software must be made available to editors and reviewers. We strongly encourage code deposition in a community repository (e.g. GitHub). See the Nature Portfolio guidelines for submitting code & software for further information.

Data

Policy information about availability of data

All manuscripts must include a data availability statement. This statement should provide the following information, where applicable:

- Accession codes, unique identifiers, or web links for publicly available datasets
- A description of any restrictions on data availability
- For clinical datasets or third party data, please ensure that the statement adheres to our policy

Source Data are provided with this paper. All data supporting the findings are available in the Figshare data repository (https://figshare.com/s/73a84b61124c0d10d0aa) and Supplementary Information.

Research involving human participants, their data, or biological material

Policy information about studies with human participants or human data. See also policy information about sex, gender (identity/presentation), and sexual orientation and race, ethnicity and racism.

Reporting on sex and gender	Not relevant to this study
Reporting on race, ethnicity, or other socially relevant groupings	Not relevant to this study
Population characteristics	Not relevant to this study
Recruitment	Not relevant to this study
Ethics oversight	Not relevant to this study

Note that full information on the approval of the study protocol must also be provided in the manuscript.

Field-specific reporting

Please select the one below that is the best fit for your research. If you are not sure, read the appropriate sections before making your selection.

☐ Life sciences ☐ Behavioural & social sciences ☒ Ecological, evolutionary & environmental sciences

For a reference copy of the document with all sections, see nature.com/documents/nr-reporting-summary-flat.pdf

Ecological, evolutionary & environmental sciences study design

All studies must disclose on these points even when the disclosure is negative.

Study description	In this study, by synthesizing 4,728 RTS incidents and 1,862 in-situ CO2 and CH4 measurements across the Tibetan Plateau, we offer a comprehensive assessment of carbon release from abrupt permafrost thaw. Our results reveal that the RTS susceptibility area will expand by 17–19% by 2100 relative to 2022, driven primarily by precipitation changes. Compared to control areas, ecosystem respiration in RTS-affected areas decreases by 14.4%, while CH4 release rate increases by 20.0%. The combined CO2 and CH4 release associated with RTS expansion increased 1.1-fold between 2016 and 2022. Under the intermediate Shared Socioeconomic Pathways scenario, carbon emissions from RTS-susceptible areas are projected to surge 2.7-fold by 2100. Our study highlights that abrupt thaw substantially strengthens permafrost carbon-climate feedbacks in high-altitude regions and underscores the urgent need for targeted permafrost protection strategies to achieve carbon neutrality goals.
Research sample	In this study, we collected annual RTS inventories across the Tibetan Plateau from published literature over the period 2016–2022, covering a total of 4,728 RTS incidents based on field observations and remote sensing data. We also conducted in-situ field monitoring at 43 RTS sites, and performed a literature review to compile carbon release data from RTSs. In total, this study compiled 291 SOCC measurements, 1,448 CO2 release measurements, and 414 CH4 release measurements from RTSs across the Tibetan Plateau. The dataset covers both growing and non-growing seasons over the period 2010–2023.
Sampling strategy	We measured ecosystem respiration (Reco), CH4 release rates, and soil organic carbon content (SOCC) at 43 RTS sites across three alpine vegetation types (alpine swamp meadow, alpine meadow, and alpine steppe). Each RTS was partitioned into three functional zones: control zone, drape zone, and exposed zone, with a minimum of three replicate sampling points per zone. Soil samples were collected from three depth intervals (0–10 cm, 10–20 cm, 20–30 cm) at each sampling point.
Data collection	Soil samples were collected from three depth intervals (0–10 cm, 10–20 cm, 20–30 cm) at each sampling point. Ecosystem respiration (Reco) was measured using a Li 8100 system at 17 RTSs. CH4 release rates were monitored at four RTSs in alpine swamp meadows using a static closed-chamber technique, with gas samples analyzed by gas chromatography.<br>For RTS susceptibility modeling, we collected 14 environmental variables from global geospatial datasets, including climate, permafrost, topographic, and soil variables, as well as the Human Influence Index. Spatial filtering was applied to retain one RTS occurrence per grid cell to reduce sampling bias. Stepwise regression selected 10 uncorrelated variables, and all data were resampled to 1 × 1 km resolution.
Timing and spatial scale	We conducted field measurements at 43 RTS sites during April–October 2021–2023 and synthesized previously published carbon release data from RTSs across the Tibetan Plateau (2010–2023). The sites cover three alpine vegetation types: alpine swamp meadow, alpine meadow, and alpine steppe.
Data exclusions	To mitigate spatial autocorrelation and sampling bias, only one RTS occurrence per grid cell was retained after spatial filtering. Four environmental variables with high intercorrelation were removed using stepwise regression.
Reproducibility	We provide all the data for analysis in Data availability . All attempts to repeat the results of this study were successful.
Randomization	In our study, sampling was not random, as we aimed to capture carbon release from different functional zones within each RTS. We

Randomization	strategically established sampling points in three distinct zones (control, drape, and exposed) to capture spatial variations in CO_2 and CH_4 emissions and soil carbon content. At each zone, a minimum of three replicate sampling points were established, and soil samples were collected from three depth intervals (0–10 cm, 10–20 cm, 20–30 cm) at each point. This approach allowed us to account for environmental factors such as vegetation cover, soil disturbance, and permafrost thaw intensity, which could influence carbon release.
Blinding	Blinding was not necessary for this study

Did the study involve field work? ☒ Yes ☐ No

Field work, collection and transport

Field conditions	The fieldwork was conducted on the Tibetan Plateau during April–October from 2021 to 2023. During this period, the average daily temperature ranged between 0°C and 10°C. The weather was typically characterized by clear skies and abundant sunshine, with occasional rain and snowfall.
Location	Eboling Mountains(Latitude: 38.0°N;Longitude: 100.9°E);Maduo(Latitude: 34.9°N;Longitude: 98.5°E);<br>Beilu River(Latitude: 34.7°N;Longitude: 92.9°E)
Access & import/export	To conduct our research responsibly, we ensured compliance with local and national regulations. Before fieldwork began, we obtained the necessary permits from relevant authorities, allowing for the collection of soil and gas samples from RTS sites across the Tibetan Plateau. All fieldwork procedures adhered to environmental protection guidelines, minimizing disturbance to the permafrost ecosystem and local vegetation.
Disturbance	Sampling was conducted with minimal impact on the permafrost environment. At each RTS site, samples were collected from three functional zones with limited physical disturbance to the soil surface and vegetation. Equipment was cleaned between sampling points to prevent cross-contamination.

Reporting for specific materials, systems and methods

We require information from authors about some types of materials, experimental systems and methods used in many studies. Here, indicate whether each material, system or method listed is relevant to your study. If you are not sure if a list item applies to your research, read the appropriate section before selecting a response.

Materials & experimental systems

n/a	Involved in the study	
☒	☐	Antibodies
☒	☐	Eukaryotic cell lines
☒	☐	Palaeontology and archaeology
☒	☐	Animals and other organisms
☒	☐	Clinical data
☒	☐	Dual use research of concern
☒	☐	Plants

Methods

n/a	Involved in the study	
☒	☐	ChIP-seq
☒	☐	Flow cytometry
☒	☐	MRI-based neuroimaging

Plants

Seed stocks	Not relevant to this study
Novel plant genotypes	Not relevant to this study
Authentication	Not relevant to this study
