# Supplementary material for: Abrupt permafrost thaw drives exceptional carbon release across the Tibetan Plateau
Source: Nat Commun. 2026 Jun 27;17:8050. doi: 10.1038/s41467-026-74850-y (PMC13454460; doi:10.1038/s41467-026-74850-y)
Supplement: Supplementary file 4 [file 41467_2026_74850_MOESM5_ESM.pdf]

nature portfolio

Peer Review File

Abrupt permafrost thaw drives exceptional carbon release across the Tibetan Plateau

Corresponding Author: Professor Cuicui Mu

This file contains all reviewer reports in order by version, followed by all author rebuttals in order by version.

Version 0:

Reviewer comments:

Reviewer #1

(Remarks to the Author)
This paper presents a thaw slump inventory on the Tibetan Plateau and analyzes the factors that dictate the susceptibility of the landscape to encounter thaw slumps. The authors then model projected evolution in the distribution of thaw slumps and the potential GHG emissions that could be expected by 2100. While I think the approach to mapping areas likely to be affected by thaw slumps in the future is very interesting and valuable, I also believe that the assumptions underlying the model of future GHG emissions are unreasonable and potentially substantially overestimate the emissions that can be expected: an exponential decline model does not reflect the actual carbon budget from thaw slumps in my opinion (see detailed comments below).

In summary, I believe this study could potentially bring significant gain to the scientific community, yet the methodology for upscaling GHG emissions needs to be substantially improved or backed up before considering possible publication. The methodology would also benefit from clarification, particularly with regard to the equations.

You will find below a series of questions/comments by section, with general and specific feedbacks for each one.

Abstract

L25: I believe there is a grammatical problem with the 'Whereas'? Is it the appropriate wording here?
L27: It might be necessary to be a bit more specific concerning the in situ monitoring data? is it from thaw slump headwalls, debris tongues, ect.?
L33: I would really avoid the terminology 'slow warming', because it is an expression that does not reflect reality, i.e., warming in permafrost regions is 2 to 4 times faster than the global average. In the literature, we use 'gradual thaw' (which is the dedicated term), which is often opposed to 'abrupt thaw' (which is sometimes encountered, particularly in wide audience papers, although it is sometimes criticized and the term 'thermokarst development' is preferred).

Introduction

General comments:

There is no precise definition of what a thaw slump is in the introduction, as LL44-46 do not constitute a clean proper definition, in my opinion. I think this is missing and could even be accompanied by a photo and/or conceptual diagram for publication in such a journal with a wide audience.
I also think there should be at least mention of the term 'hillslope thermokarst terrain' somewhere, as 'abrupt thaw' is sometimes criticized (i.e., a dramatic increase in active layer depth following a warm summer could also be described as 'abrupt' without necessarily being called 'thermokarst').

Specific comments:

L44: concerning acceleration of thaw slumping, there might be other references to cite, e.g.,
- Kokelj et al. (2015). Glob. Planet. Change 129, 56–68
- Segal et al. (2016). Environ. Res. Lett. 11, 034025.

L45: Often, the materials exposed by thaw slumps are not soils in the pedological sense, but sediments deposited syngenetically with permafrost aggradation. I therefore wouldn't use the expression 'several meters of permafrost-affected soils' in this context.
L48: While I think there is consensus on the permafrost-carbon-climate feedback on a global scale, claiming that thaw slumps create a 'crucial positive feedback' seems a bit hasty to me... To my knowledge, only one study has attempted – to date – to estimate this on an Arctic scale (Turetsky, et al. (2020). Nat. Geosci. 13, 138–143), but other local studies are not always in line with this... As stated a few lines further on, there remains considerable uncertainty regarding the carbon feedback of deep thaw features such as thaw slumps, and I think this statement is too strong. I would therefore use the conditional tense to describe the potential carbon feedback of thaw slumps.
LL87-88: Again, I would avoid the expression 'slow warming'.

Methods

General comments:

I believe that the methodological development would greatly increase in clarity if it were accompanied by a conceptual diagram and/or equations with appropriate units to support the approach. In particular, in LL523-540, there is a presentation of the methodology for selecting the parameters and variables that are most correlated with the occurrence of thaw slump features. Some of these are fixed (elevation, slope, ground ice, grain size, ect..), and others are dynamic (precipitation, air & ground temperature). From this, the authors created a kind of risk map for the occurrence of RTS (LL542-558), but some points are not clear to me:
- should we understand that this is an indicator of where the risk is high according to the model; are there units?
- is the output the decision score of the Random Forest?
- What is the unit of training and output data for the Random Forest? Is it the proportion of the landscape affected by observed RTS? Is it a 0–1 prediction with occurrence or no occurrence? It is very important that this is clear to the reader in order to understand the maps.
At the same time (LL560-571), the authors perform a sensitivity analysis to quantify the influence of input parameters or variables on the map. Unless I am mistaken, Random Forest enables to do this directly (feature importance).
Then, equation 1 shows that the carbon stock in the system can only decrease, through a decay function, presumably exponential. How can the authors be sure that this approach is adequate? Isn't it possible that the carbon stock could increase over time under certain conditions? In fact, the materials in scar zones & debris tongues of thaw slumps are replenished with fresh material as the collapsed front retreats each summer, and some is exported further downslope. It appears that the model does not take this into account, nor the fact that RTSs stabilize at a certain point, at which stage the materials in the scar zone form a new active layer with vegetation growth that can likely capture carbon. Furthermore, if I understand the approach correctly, the initial organic carbon stock in the equations comes from the SOCC, which itself only takes into account the top 30 centimeters of soil. However, the materials exported by thaw slumps come from much greater depths, up to several (tens of) meters. The carbon stock at these greater depths is highly variable, but likely much lower than in the surface layers (see e.g., Bernhard et al. (2022). The Cryosphere 16, 2819–2835; Thomas et al. (2023). Geoderma 433, 116443). A plausible initial organic carbon content in the scar zone will therefore likely be much lower than what is obtained from these calculations. I do not believe that the presented model is validated by a true balance assessment of the carbon stock in materials exposed by thaw slumps. I therefore feel that the approach, based on the assumptions made, potentially significantly overestimates the projected emissions from such deep thaw features.

Specific comments:

LL494-498: It is not clear to me whether this processing was carried out as part of this study or whether it was done in the studies from which the inventories were obtained, i.e., refs 8,23? Could the authors clarify?
LL504-505ff: I am a bit puzzled that the SOC content discussed in the study is limited to depths of maximum 30 cm; thaw slumps actually expose headwalls up to several (tens of) meters deep. Furthermore, it is not very clear whether these are samples from the headwall or debris tongues, or elsewhere? Also, could the authors clarify the exact methodology for the measurements of GHG fluxes and Reco?
LL519-520: I don't understand the end of the sentence. Where can these monthly mean values be found?
L525: I don't fully understand what Suppl. Tab. 3 shows.
L560: Can the authors define precisely what is meant by 'RTS susceptibility' in this paragraph and the previous one? Is it the area susceptible to being affected by an RTS? The portion of the area of one pixel?
L561: Can the authors define 'SHAP model'? the acronym?
Suppl. Fig. 10: In terms of morphology, what is presented here resembles a thermo-erosional gully more than a thaw slump. If this type of structure is frequently repeated at the sites available in this study, I believe that the terminology should be adapted to refer instead to hillslope thermokarst structures, clearly specifying that this encompasses many different geomorphological structures. This is particularly important for the CO_2/CH_4 ratio, which is likely to vary rather strongly between thermo-erosional gullies and thaw slumps. I believe that adjustments should be made where necessary.

Results and discussion

General comment:

With regard to my comments above on the assumptions of the model for carbon emissions, I am rather skeptical about the GHG emissions presented in this section.

Specific comments:

LL107ff: The observation regarding the increase in CH4 flux seems surprising to me. Redox conditions following thaw slumping are considered to be oxic and relatively stable (e.g., Abbott & Jones (2015). Glob. Change Biol. 21, 4570–4587). Do the authors have any idea why they are observing this increase? In the study by Yang et al. (2018) cited below, it would appear that these are somewhat specific conditions, and I am not sure that they can be generalized to the entire Tibetan Plateau.
Fig. 1e-f-g: Are the increases and decreases pointed out significant?
LL284-285: Are the 291 measurements of SOCC losses really observations? It's not very clear to me from the methodology.

Reviewer #2

(Remarks to the Author)
1)The authors clearly demonstrate that RTS expansion between 2016–2022 resulted in a 3.2-fold increase in CO_2 emissions and a 6.2-fold increase in CH_4 emissions. However, the manuscript would benefit from a more in-depth discussion of the processes driving these increases in carbon emissions.
2)Line112: The term meta-analyses may not be the most appropriate in this context; consider whether data synthesis would be more accurate.
3)Line 215: Please ensure consistent use of related terms such as slow warming, gradual thawing, and gradual permafrost thaw throughout the manuscript.
4)The manuscript mentions the use of Q_{10}. Please provide additional explanation in the Methods section or Supplementary Materials to justify its selection and application.
5)The manuscript mentions the used of Monte Carlo simulations to estimate RTS-induced carbon release and its uncertainty. Could the authors clarify how this approach was specifically integrated into the carbon release calculations?
6)Please specify the exact years and time span of the carbon monitoring datasets used. Including this information for all datasets will enhance the temporal representativeness of the simulation results.
7)The Discussion section could be strengthened by elaborating on seasonal variations in carbon release from thermokarst, exploring driving factors such as microbial activity, soil moisture, and temperature. Additionally, please discuss potential mechanisms by which vegetation types influence thermokarst carbon release, for example through differences in carbon components such as lignin content.
8)Line 112: When the term control area first appears in the manuscript, please provide a clear definition. Alternatively, consider replacing it with a more precise term, such as non-collapsed area, which corresponds more directly to collapsed area.
9)Line 124–126: The text states that vegetation destruction reduces autotrophic respiration, yet also claims that CO_2 release rates in the subsidence area increase due to higher temperature sensitivity. The logical connection between these two points requires clarification. Please consider adding supporting data or citing relevant references; otherwise, the current phrasing may lead to contradictory interpretations.
10)Line 282: Please clarify what type of “uncertainties” are being referred to in this context.
11)Line 338: Since RTS volume can influence carbon release, please add supporting references.
12)Line 111: When describing CH_4 flux in relation to RTS, it is preferable to consistently use terms such as induced or influenced throughout the manuscript for accuracy and consistency.
13)The integration of Shapley Additive Explanation (SHAP) analysis to interpret drivers of RTS susceptibility is innovative，The SHAP model may needs a few descriptive contents of description.
14)Methods: It is suggested to enrich the methods section, particularly, sensitivity analysis may need a few descriptive contents of description.
15)It is, to my knowledge, the first study to provide comprehensive simulations of future RTS emissions across the QTP using SSPs. The author should explain why these three climate scenarios were chosen
16)Line 522: This part of the content should describe the collection and processing of data, the title should be Climate and environmental variables.
17)RTS susceptibility mapping and Drivers and uncertainty of RTSs susceptibility should be further sorted out.
18)The availability of code and data provided in the Machine Learning Checklist should be clearly stated in the text.

Version 1:

Reviewer comments:

Reviewer #2

(Remarks to the Author)
The novelty and key improvements are as follows:
1.Clarity of Simulation Scope: The refined focus on “active RTS” appropriately mitigated the risk of overestimation inherent indelimitation extrapolating from active features to all RTSs. Equally important was the clear of CH4 simulations to waterlogged alpine swamp meadows, which appropriately bounded the analysis and enhanced interpretability.
2.Model Framework Advancement: A key improvement noted in the Methods section was the replacement of the single-pool model with a two-pool framework. This was a significant and appropriate upgrade, as the two-pool model is the most widely applied and suitable framework for simulating carbon release in permafrost regions, as it better represents distinct carbon decomposition dynamics. This revision was directly substantiated by the notably lower carbon release estimates in the updated results, which clearly demonstrated the new model’s effectiveness in constraining the previous overestimation.
3.Mechanistic Refinement: The integration of deep-soil decomposition processes represented a critical refinement.

Importantly, the model optimization utilized incubation data from various vegetation types across the RTS area, incorporating temperature and moisture response functions to mechanistically constrain carbon release.
Collectively, these revisions, along with a notably deepened discussion of the results in response to reviewer comments, markedly improved the manuscript's methodological rigor, scientific coherence, and overall impact. I have only one minor editorial note: please ensure the consistency of figure and panel labeling throughout the manuscript, as panel "b" appears to be missing in Supplementary Figure 12. Beyond this, I have no further questions or concerns regarding this revised version.

Reviewer #4

(Remarks to the Author)

This study estimates CO2 and CH4 emissions induced by retrogressive thaw slumps (RTSs) and develops models to project future changes in these gases under different climatic conditions. Overall, the manuscript is well written and presents interesting results. The findings highlight the importance of permafrost protection and relevant carbon management in permafrost-affected regions.

I have the following comments, which I hope will help further improve the manuscript:

I understand that the manuscript focuses on carbon release in terms of CO2 and CH4. However, it is important to recognize that the overall soil carbon balance is more relevant. This limitation should be clearly acknowledged. Since RTSs are in the early stages of vegetation succession, any claims regarding whether they function as carbon sources or sinks should be made with caution. Without quantifying carbon inputs, conclusions about source–sink dynamics are not sufficiently supported (e.g., line 261).

In permafrost-affected regions, permafrost depth is a critical variable influencing thaw dynamics. This parameter likely has significant effects on the spatiotemporal patterns of CO2 and CH4 emissions and should therefore be considered in the driver analysis.

It would also be valuable to include results or discussion on the drivers of RTS changes during the study period. In particular, it is unclear why precipitation, rather than temperature, is identified as the most important predictor of RTS distribution. This appears inconsistent with statements elsewhere in the manuscript (e.g., line 237), where higher temperatures are described as the main controlling factor. Please carefully review the analysis and clarify this point, or better contextualize the apparent discrepancy. A key question is whether increases in RTSs are driven primarily by rising temperature, increasing precipitation, or a combination of both.

Future projections constitute a substantial portion of the manuscript (as also noted by a previous reviewer). However, the two-pool carbon model used does not appear to include carbon inputs or transformations among pools. This structure may be overly simplistic for in situ predictions. Such models are typically more appropriate for incubation studies. When conducting projections, model structure is a critical consideration—often more important than parameter uncertainty, which has already been thoroughly quantified in this study. My suggestion is to drop this part and just use the RF model.

If I understand correctly, the authors fitted 50 random forest (RF) models to estimate uncertainty. However, all models are trained on essentially the same empirical dataset. As a result, this approach captures only algorithmic randomness and does not account for data uncertainty (e.g., sampling variability) or model structural uncertainty. Consequently, the reported uncertainty is likely underestimated. A more common approach is to use the distribution of predictions from individual trees within a single random forest to quantify predictive uncertainty.

Reviewer #5

(Remarks to the Author)
This study addresses an important and timely question in abrupt permafrost thaw. The work is well-motivated, and the authors succeed in demonstrating the RTS evolution and their induced carbon emissions on the Tibetan Plateau. This manuscript presents a valuable dataset of spatiotemporal distribution of RTS susceptibility using machine learning models. In addition, the carbon modeling builds on the Beer et al. (2023) framework, which first applied a two-pool model to thaw slump carbon release. This extends this approach in several meaningful ways: incorporating soil moisture parameters, conducting vegetation-specific modeling to account for ecological heterogeneity, and validating the model against field measurements. In addition, the dataset of 1,826 monitoring records, which includes non-growing season observations across three vegetation types, provides a solid empirical basis for this validation.

The revised manuscript has been improved through the detailed responses to all comments. I believe this manuscript will make a meaningful contribution to the field of permafrost change. I thus recommend acceptance after a minor revision.The following minor issues could be addressed to further improve clarity:

1. For the monitored 61 RTS sites, the discussion about the differences in carbon losses caused by RTS in alpine swamp meadows and steppes should be strengthened, in order to clarify the impacts on soil carbon contents.
2. In the results and discussion, the CH4 release rate induced by RTSs focuses on the alpine swamp meadow; other vegetation types should be shown or the relevant data should be introduced.
3. Non-growing season estimation: The use of fixed seasonal ratios (CO_2: 0.38; CH_4: 0.18) to estimate non growing season

release is a practical approach. A brief note on whether these ratios showed variability across vegetation types or monitoring years would provide helpful context.
4. In the figure 3b, it is necessary to clarify what the shaded areas represent at current and in the future.
5. The manuscript does not provide a comparison between the carbon release rate (46% concentrated between 100 and 150 g C m^{-2} yr^{-1}) mentioned and the carbon release from soil respiration during gradual permafrost warming. It would be beneficial to clarify the potential of carbon emissions caused by RTSs.
6. Supplementary Figure 16 shows the model data comparison for CO_2 release rates. Clarify whether the data points represent daily values, seasonal averages, or monthly means. Additionally, specifying which seasons or months are included would help readers better interpret the validation results.
7. Model validation: The authors report strong validation metrics (R^2=0.915, RMSE=0.915 g C m^{-2} d^{-1}). Clarify whether these metrics represent performance across all vegetation types combined or were calculated separately for each type.
8. There are some uncertainties in future projection of carbon emissions due to high spatial heterogeneity, and it would be valuable to add some discussion on the simulated changes in RTS on the Tibetan Plateau.

Version 2:

Reviewer comments:

Reviewer #2

(Remarks to the Author)
I have carefully reviewed your revised manuscript and the detailed point-by-point responses to the reviewers' comments. I appreciate the significant effort you have put into addressing the concerns raised.

Reviewer #4

(Remarks to the Author)
The authors did a good job for the revision. I do not have other comments.

Reviewer #5

(Remarks to the Author)
All my concerns have been addressed, I have no further comments.

Open Access This Peer Review File is licensed under a Creative Commons Attribution 4.0 International License, which permits use, sharing, adaptation, distribution and reproduction in any medium or format, as long as you give appropriate credit to the original author(s) and the source, provide a link to the Creative Commons license, and indicate if changes were made.
In cases where reviewers are anonymous, credit should be given to 'Anonymous Referee' and the source.

The images or other third party material in this Peer Review File are included in the article's Creative Commons license, unless indicated otherwise in a credit line to the material. If material is not included in the article's Creative Commons license and your intended use is not permitted by statutory regulation or exceeds the permitted use, you will need to obtain permission directly from the copyright holder.
To view a copy of this license, visit https://creativecommons.org/licenses/by/4.0/

REVIEWER COMMENTS

Responses to Reviewers of NCOMMS-25-60807

Reviewer #1 (Remarks to the Author):
This paper presents a thaw slump inventory on the Tibetan Plateau and analyzes the factors that dictate the susceptibility of the landscape to encounter thaw slumps. The authors then model projected evolution in the distribution of thaw slumps and the potential GHG emissions that could be expected by 2100. While I think the approach to mapping areas likely to be affected by thaw slumps in the future is very interesting and valuable, I also believe that the assumptions underlying the model of future GHG emissions are unreasonable and potentially substantially overestimate the emissions that can be expected: an exponential decline model does not reflect the actual carbon budget from thaw slumps in my opinion (see detailed comments below).

In summary, I believe this study could potentially bring significant gain to the scientific community, yet the methodology for upscaling GHG emissions needs to be substantially improved or backed up before considering possible publication. The methodology would also benefit from clarification, particularly with regard to the equations.

You will find below a series of questions/comments by section, with general and specific feedbacks for each one.

Response: Thank you for your valuable comments, particularly your recognition of our study focusing on the dynamics of retrogressive thaw slumps (RTSs) on the Tibetan Plateau. We fully agree with your concern regarding the limitations of the exponential decline model in estimating carbon emissions. According to your constructive suggestions, we have carefully revised and refined our methodology. Specifically, a two-pool model has been used to simulate carbon emissions from RTSs in the revised manuscript (*see updated Method*). This method has been widely adopted in previous permafrost carbon studies (Beer et al., 2023; Qin et al., 2019), and has been demonstrated to reliably capture carbon emissions from thaw slumps in northern permafrost regions.

*Beer, C., Runge, A., Grosse, G., Hugelius, G. & Knoblauch, C. Carbon dioxide release from retrogressive thaw slumps in Siberia. Environ. Res. Lett. **18**, 104053 (2023).*

*Qin, S. et al. Temperature sensitivity of SOM decomposition governed by aggregate protection and microbial communities. Sci. Adv. **5**, eaau1218 (2019)*

Additionally, we have enhanced our methodology by integrating both surface and deep soil carbon stocks and accounting for the effect of RTS in the active stage (*see updated Method*). Further, we have updated the Results and discussions, and added an analysis of uncertainties in the revised version. Below, we provide detailed responses to each of your comments. We hope our endeavor has fully addressed your valuable suggestions.

Abstract

L25: I believe there is a grammatical problem with the 'Whereas'? Is it the appropriate wording here?

Response: It has been changed into "*However*".

L27: It might be necessary to be a bit more specific concerning the in-situ monitoring data? is it from thaw slump headwalls, debris tongues, ect.?

Response: Thanks for your detailed comments. It has been revised in the abstract and methods as follows:

Lines 27-29: "*Here, by synthesizing 4,728 RTS incidents and 1,878 in-situ CO_2 and CH_4*

monitoring data from the collapsed areas of RTSs on the Tibetan Plateau, ..."

To enhance the clarity of the sampling site descriptions, we have added the relevant details in the Methods as follows:

Lines 521-527: "*To ensure standardized sampling design and data comparability, each RTS was partitioned into three functional distinct areas: (1) a control zone (an undisturbed permafrost area situated at least 5 meters away from the RTS boundary), (2) a drape zone (a vegetated area adjacent to the slump margin or distributed within the debris tongue), and (3) an exposed zone (vegetation-free bare soil areas). For each zone, a minimum of three replicate sampling points were systematically established (Supplementary Fig.12).*"

L33: I would really avoid the terminology 'slow warming', because it is an expression that does not reflect reality, i.e., warming in permafrost regions is 2 to 4 times faster than the global average. In the literature, we use 'gradual thaw' (which is the dedicated term), which is often opposed to 'abrupt thaw' (which is sometimes encountered, particularly in wide audience papers, although it is sometimes criticized and the term 'thermokarst development' is preferred).
Response: We agree with your detailed comment. Accordingly, it has been revised into "*gradual thaw*" to enhance the clarify of our findings for readers.

Introduction
General comments:
There is no precise definition of what a thaw slump is in the introduction, as LL44-46 do not constitute a clean proper definition, in my opinion. I think this is missing and could even be accompanied by a photo and/or conceptual diagram for publication in such a journal with a wide audience.

I also think there should be at least mention of the term 'hillslope thermokarst terrain' somewhere, as 'abrupt thaw' is sometimes criticized (i.e., a dramatic increase in active layer depth following a warm summer could also be described as 'abrupt' without necessarily being called 'thermokarst').
Response: Thanks for your valuable and constructive comment. We have added the definition of retrogressive thaw slumps (RTSs) and introduced the term "hillslope thermokarst" in the Introduction. The specific revisions are detailed as follows:

Lines 42-45: "*As the most severe instances of abrupt permafrost thaw, retrogressive thaw slumps (RTSs) are accelerating expansion due to the melting of ground ice*[6-10]*. RTSs represent one of the typical hillslope thermokarst landscapes, characterized by the melting of ground ice and the subsequent subsidence or collapse of thawed material*[11,12]."

Additionally, a field photo for monitoring a representative RTS has been supplemented in the updated Method (Supplementary Figure 12).

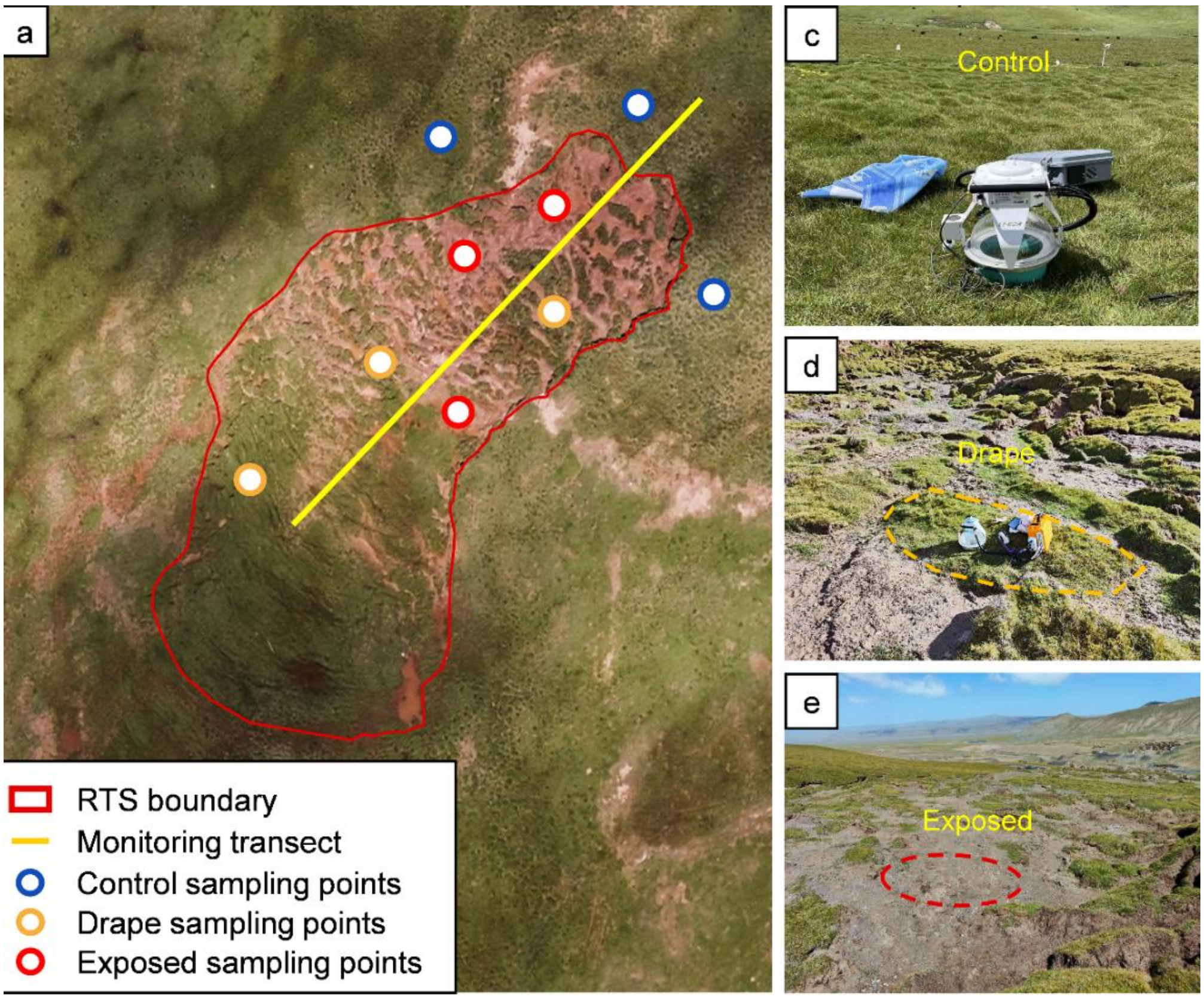

Supplementary Fig. 12 In-situ carbon monitoring sites within a representative RTS. *It schematically depicts the partitioning of the RTS into three distinct functional zones: control zone, drape zone, and exposed zone, which presents the typical layout of sampling points designed for gas flux and soil property measurements.*

Specific comments:
L44: concerning acceleration of thaw slumping, there might be other references to cite, e.g.,
- Kokelj et al. (2015). Glob. Planet. Change 129, 56–68
- Segal et al. (2016). Environ. Res. Lett. 11, 034025.

Response: Yes, the references have been updated.

Lines 42-43: "*As the most severe instances of abrupt permafrost thaw, retrogressive thaw slumps (RTSs) are accelerating expansion due to the melting of ground ice*[6-10]."

L45: Often, the materials exposed by thaw slumps are not soils in the pedological sense, but sediments deposited syngenetically with permafrost aggradation. I therefore wouldn't use the expression 'several meters of permafrost-affected soils' in this context.

Response: Thanks for your detailed comment. It has been revised as follows:

Lines 45-47: "*They can destabilize hillslope systems and expose several meters of sediments deposited during permafrost aggradation*[12]*, which exert substantial impacts on carbon release processes*[13-16]."

L48: While I think there is consensus on the permafrost-carbon-climate feedback on a global scale, claiming that thaw slumps create a 'crucial positive feedback' seems a bit hasty to me... To my knowledge, only one study has attempted – to date – to estimate this on an Arctic scale (Turetsky, et al. (2020). Nat. Geosci. 13, 138–143), but other local studies are not always in line with this... As stated a few lines further on, there remains considerable uncertainty

regarding the carbon feedback of deep thaw features such as thaw slumps, and I think this statement is too strong. I would therefore use the conditional tense to describe the potential carbon feedback of thaw slumps.

Response: According to your valuable comment, it has been revised as follows:

Lines 47-50: "*Particularly, RTSs can release large amounts of CO_2 and CH_4 into the atmosphere by significantly altering microtopographic conditions and soil hydrological processes*[16-18]*. This carbon release further accelerates global warming, potentially forming a positive climate feedback loop*[5,19,20]*.*"

LL87-88: Again, I would avoid the expression 'slow warming'.

Response: Thanks. It has been revised as "gradual thaw".

Methods

General comments:

I believe that the methodological development would greatly increase in clarity if it were accompanied by a conceptual diagram and/or equations with appropriate units to support the approach. In particular, in LL523-540, there is a presentation of the methodology for selecting the parameters and variables that are most correlated with the occurrence of thaw slump features. Some of these are fixed (elevation, slope, ground ice, grain size, ect.), and others are dynamic (precipitation, air & ground temperature). From this, the authors created a kind of risk map for the occurrence of RTS (LL542-558), but some points are not clear to me:

Response: Thank you for your valuable suggestions. We fully agree with that a clear flowchart can significantly enhance readers' understanding of our research methodology. Accordingly, we have added a detailed flowchart (Supplementary Fig. 5) to illustrate the overall process for estimating the changes of RTS susceptibility and simulating the related carbon emissions on the Tibetan Plateau.

This study primarily comprises five components: (1) **Data collection and processing**: They involve the compilation of RTS inventory and preprocessing of environmental factor data. (2) **Model construction and validation**: This describes the division of the dataset into training and testing subsets, followed by comprehensive model performance evaluation and systematic uncertainty analyses. (3) **Pattens and driving factors of RTSs**: This outlines the methodology for identifying dominant controlling factors across different regions, leveraging the SHAP (SHapley Additive exPlanations) model and sensitivity analysis techniques. (4) **RTSs susceptibility mapping and future projections**: This includes the simulation of current RTS susceptibility and the prediction of its future changes under three climate scenarios. (5) **Estimation of carbon release**: This involves the application of a carbon decomposition model to quantify historical and projected future carbon emissions associated with RTSs.

Further, we have added the related information in the method to enhance the readability and logic of this manuscript as follows:

Lines 488-497: "*This study first presents the actual distribution of 4,728 RTSs and their impacts on soil carbon loss and greenhouse gas emissions, based on in-situ observations on the Tibetan Plateau. Subsequently, we processed the RTS inventory dataset and data for 9 environmental variables. Further, a suite of single and ensemble machine learning models was employed to estimate the pattens and drivers of RTS susceptibility, and to generate projections of RTS susceptibility under current and future climate scenarios. Finally, carbon release associated with RTS development was estimated (Supplementary Fig. 5). Specifically, this method encompasses five components: data collection and processing, model construction and validation, pattens and driving factors of RTS, RTS susceptibility mapping and future projections, and estimation of carbon release.*"

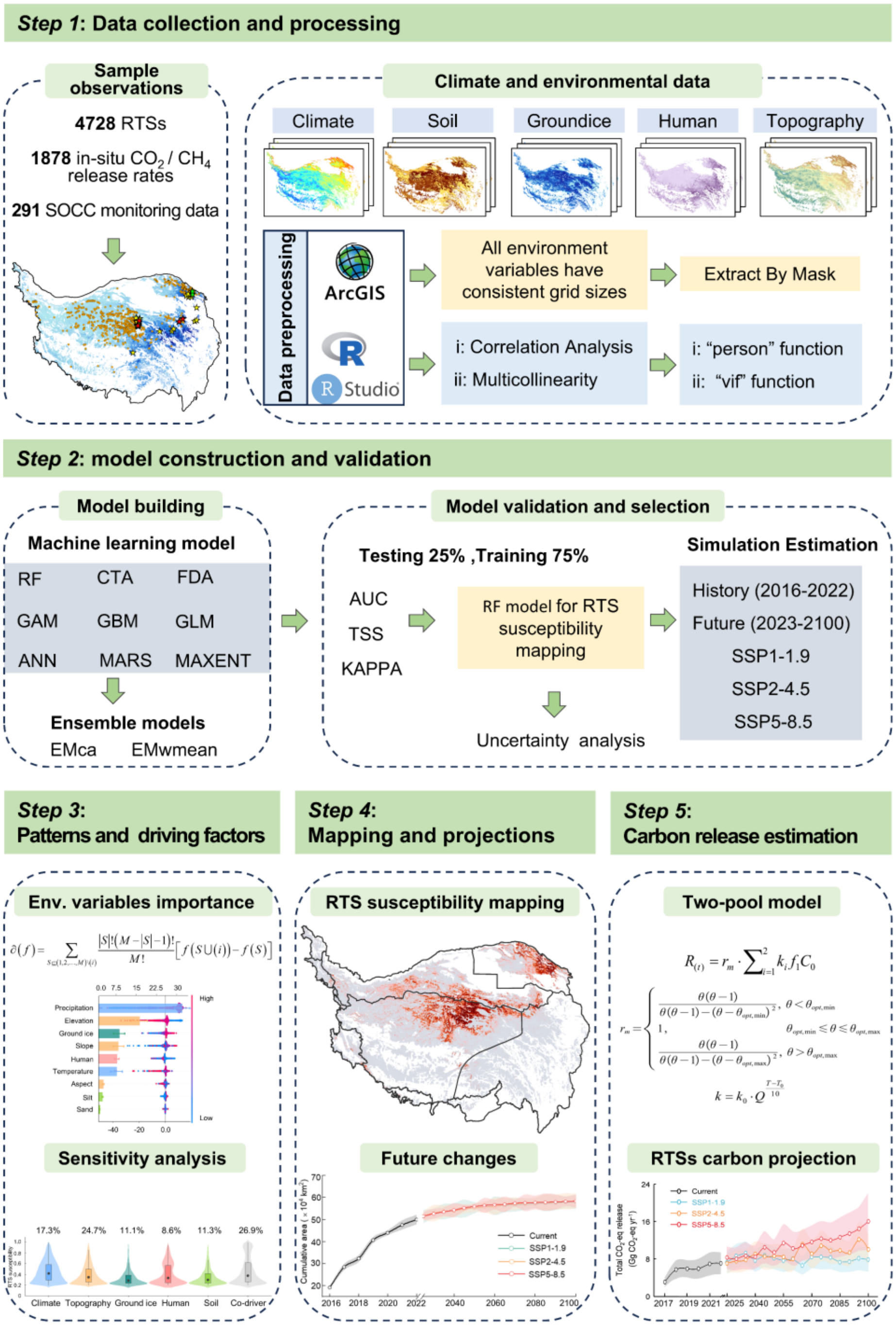

Supplementary Fig. 5 The study flowchart to illustrate the changes of RTS susceptibility and carbon release on the Tibetan Plateau.

Should we understand that this is an indicator of where the risk is high according to the model; are there units?

Response: The RTS susceptibility index ranging from 0 to 1 is unitless, showing the probability of RTS occurrence with low to very high levels. The related information has been added in the revised version.

Lines 595-601: "*The RTS susceptibility distribution is represented as a relative probability index from 0 to 1, reflecting the probability of RTS occurrence (from low to very high) under various environmental conditions. Referring to the classification standard*[65]*, RTS*

susceptibility was categorized into five levels: very low (0-0.2), low (0.2-0.4), moderate (0.4-0.6), high (0.6-0.8), and very high (0.8-1). Regions with a probability index below 0.2 were defined as control areas, whereas those with an index equal to or exceeding 0.2 were designated as RTSs-influenced areas."

Is the output the decision score of the Random Forest?

Response: Yes. The output value for each pixel is fundamentally derived from the ensemble-averaged decision score (or probability estimate) of the Random Forest model. This have been supplemented in the revised version.

Lines 587-595: "*The spatial distribution of RTS susceptibility was simulated using the BIOMOD_Projection function integrated within the biomod2 package. This function deploys the pre-trained ensemble model to environmental raster layers, enabling the prediction of RTS occurrence probability for each individual pixel across the study area. The resulting output is a georeferenced raster dataset, where each cell value corresponds to the ensemble-averaged weighted probability of RTS susceptibility, with values ranging from 0 (very low) to 1 (very high). For the Random Forest (RF), 50 independent runs were conducted; in each run, the dataset was randomly partitioned into 75% training data and 25% testing data. The final RTS susceptibility map was generated by averaging the results from all 50 outcomes.*"

What is the unit of training and output data for the Random Forest? Is it the proportion of the landscape affected by observed RTS? Is it a 0–1 prediction with occurrence or no occurrence? It is very important that this is clear to the reader in order to understand the maps.

Response: To clarify the method, we rewritten the related information in the revised version.

Lines 587-601: "*The spatial distribution of RTS susceptibility was simulated using the BIOMOD_Projection function integrated within the biomod2 package. This function deploys the pre-trained ensemble model to environmental raster layers, enabling the prediction of RTS occurrence probability for each individual pixel across the study area. The resulting output is a georeferenced raster dataset, where each cell value corresponds to the ensemble-averaged weighted probability of RTS susceptibility, with values ranging from 0 (very low) to 1 (very high). For the Random Forest (RF), 50 independent runs were conducted; in each run, the dataset was randomly partitioned into 75% training data and 25% testing data. The final RTS susceptibility map was generated by averaging the results from all 50 outcomes. The RTS susceptibility distribution is represented as a relative probability index from 0 to 1, reflecting the probability of RTS occurrence (from low to very high) under various environmental conditions. Referring to the classification standard[65], RTS susceptibility was categorized into five levels: very low (0-0.2), low (0.2-0.4), moderate (0.4-0.6), high (0.6-0.8), and very high (0.8-1). Regions with a probability index below 0.2 were defined as control areas, whereas those with an index equal to or exceeding 0.2 were designated as RTSs-influenced areas.*"

At the same time (LL560-571), the authors perform a sensitivity analysis to quantify the influence of input parameters or variables on the map. Unless I am mistaken, Random Forest enables to do this directly (feature importance).

Response: Thank you for the detailed comment. We conducted an additional one-variable at a time exclusion sensitivity analysis to derive more detailed and spatially interpretable results than standard feature importance. There are two key reasons: (1) Feature importance only yields a global ranking of variable significance, whereas our exclusion-based approach directly quantifies and visualizes the spatial distribution of each variable's influence, explicitly identifying regions where specific factors dominate. (2) The aim of this study is to map the spatial patterns of controlling factors of RTS. Sensitivity analysis outputs are inherently spatial, which aligns perfectly with this objective and offers far greater spatial granularity than a single

aggregated ranking.

Taken together, we regard feature importance as a useful preliminary screening tool, and sensitivity analysis as a more in-depth diagnostic method. The latter provides direct, detailed spatial evidence to support our research aim of mapping controlling factors. In this revision, we have carefully revised the relevant method as follows:

Lines 609-628: "*To quantify the effects of environmental variables on RTS susceptibility, we conducted a two-step analytical framework, specifically, combining the SHapley Additive exPlanations (SHAP) algorithm and sensitivity analysis to disentangle their respective contributions and spatial heterogeneity. The SHAP algorithm utilizes Shapley values to quantitatively assess the marginal contribution of each feature to model outputs[66], and was employed herein to holistically quantify the overall contribution of each environmental variable to RTS susceptibility. The sensitivity analysis was designed to identify the dominant driving factors of RTSs susceptibility at the pixel scale (1 × 1 km²), thereby capturing fine-grained spatial variations in the importance of influencing factors.*

Specifically, we applied the SHAP algorithm using the Python package "shap" to quantify the relative influence of various environmental variables on RTSs susceptibility across the entire study area. Further, based on our final random forest (RF) model, we performed a sensitivity analysis to identify primary drivers of RTSs susceptibility in the permafrost regions67. This sensitivity analysis proceeded: first, we predicted RTSs susceptibility for each 1 × 1 km² pixel using the full set of environmental variables; second, we quantified the relative sensitivity of RTSs susceptibility to each variable by comparing the performance of the full RF model with that of "reduced models", each excluding one environmental variable at a time. For each pixel, the dominant driver was defined as the variable with the highest sensitivity value (⩾0.2). If no single variable met this threshold, the pixel was classified as being influenced by "co-drivers"."

Then, equation 1 shows that the carbon stock in the system can only decrease, through a decay function, presumably exponential. How can the authors be sure that this approach is adequate? Isn't it possible that the carbon stock could increase over time under certain conditions? In fact, the materials in scar zones & debris tongues of thaw slumps are replenished with fresh material as the collapsed front retreats each summer, and some is exported further downslope. It appears that the model does not take this into account, nor the fact that RTSs stabilize at a certain point, at which stage the materials in the scar zone form a new active layer with vegetation growth that can likely capture carbon. Furthermore, if I understand the approach correctly, the initial organic carbon stock in the equations comes from the SOCC, which itself only takes into account the top 30 centimeters of soil. However, the materials exported by thaw slumps come from much greater depths, up to several (tens of) meters. The carbon stock at these greater depths is highly variable, but likely much lower than in the surface layers (see e.g., Bernhard et al. (2022). The Cryosphere 16, 2819–2835; Thomas et al. (2023). Geoderma 433, 116443). A plausible initial organic carbon content in the scar zone will therefore likely be much lower than what is obtained from these calculations. I do not believe that the presented model is validated by a true balance assessment of the carbon stock in materials exposed by thaw slumps. I therefore feel that the approach, based on the assumptions made, potentially significantly overestimates the projected emissions from such deep thaw features.

Response: We sincerely appreciate your valuable comments and suggestions. In this revision, we have updated the method by using a two-pool model, which integrate data from different soil depths and account for the active stage of RTSs. The specific revisions have been summarized as follows:

1. Simulation of carbon emissions for the active stage of RTSs:

The estimation of carbon emissions has been revised for the active stage of RTS

development, which was defined by annual area expansion and new formation from 2016 to 2022. Consequently, all field observations in this study including carbon release measurements and soil organic carbon sampling were conducted for the active RTSs. In addition, for the future projection, the distribution of active RTSs was represented by susceptibility-level changes: grids with increasing susceptibility or those sustaining a very high level were classified as the active RTSs.

Lines 84-86: "*Subsequently, we estimated the spatial variations in CO_2 and CH_4 release derived from actively developing RTSs from 2017 to 2022 by optimized a two-pool model*[8,26]."

Lines 677-678: "*Before upscaling carbon release across the Tibetan Plateau, we defined the active stage of RTSs as those exhibiting annual area expansion and new slump formation during the period of 2016-2022*[26]."

Lines 719-723: "*To project future carbon release from active RTSs, we applied the optimized two-pool model combined with a vegetation-stratified RTS susceptibility map. The future distribution of active RTSs was defined by susceptibility-level changes: grids that showed an increase in susceptibility level or maintained a very high level relative to the preceding year were designated as active RTSs.*"

2. Considering carbon inputs and deep carbon stocks:

Given that existing RTS datasets fail to capture internal microtopographic heterogeneity, the carbon release from collapsed areas was represented by site-averaged measurements. Additionally, carbon inputs exhibit a strong dependence on slump depth, which cannot be robustly constrained using current RTS datasets. Thus, a conservative assumption of a balance between carbon inputs and outputs was adopted to the entire active stage of RTSs, thereby precluding the application of an exponential decay function for surface soil.

Lines 678-683: "*Since the RTS distribution data are unable to delineate microtopographic units within collapsed areas (e.g., drape and exposed zones), the measured carbon release data were averaged across these units to represent the overall carbon release of the entire collapsed area. Furthermore, for the surface soils in the collapsed area, we assumed that carbon inputs are balanced by outputs, thereby omitting the exponential decay function in the spatial simulations.*"

In addition, to avoid the potential overestimation of initial soil organic carbon stocks, we compared SOCC in the 0–2 m depth between collapsed and control areas. The results showed no significant difference in SOCC below 30 cm depth (Supplementary Fig. 15). Therefore, SOC stocks in the 30–100 cm layer of control areas were adopted as the initial stocks for the corresponding depth in collapsed areas. Exponential decay was exclusively applied to the deep-layer carbon pool.

Lines 683-688: "*Furthermore, for surface soils in collapsed area, we assumed a balance between carbon inputs and outputs, thereby omitting the exponential decay function in spatial simulations. Surface soil organic carbon content (SOCC, 0–30 cm) in RTS-collapsed areas was estimated as the residual value following the application of vegetation-specific and depth-specific loss rates derived from data synthesis (Equations 6; Supplementary Fig. 2). In contrast, the deep SOCC (30-100 cm) was assumed to remain unaltered (Supplementary Figs. 15).*"

3. A two-pool model simulation and validation:

To enhance the robust of results, we modified our method by using a two-pool model (Qin et al., 2019) instead of a single-pool model to better represents the dynamics of soil carbon decomposition. Model parameters were determined and calibrated through the following steps:

(1) Initial parameters and ranges were estimated using soil incubation data from collapsed areas under three alpine vegetation types (swamp meadow (Mu et al.,2024), meadow (Zhao et al., 2025), and steppe (Li et al.,2019)). Data from control area at a 1.5 m depth were used to inform the decomposition parameters of deep soil (Qin et al.,2021).

$$R = r_m \cdot \sum_{i=1}^{2} k_i f_1 C_0$$

Lines 651-654: "*We employed an optimized two-pool carbon decomposition model to simulate carbon release driven by active RTSs on the Tibetan Plateau. By integrating the model with regional RTS distribution, we quantified the interannual variation of carbon release for the active RTSs from 2017 to 2022 (Equation 1).*"

Lines 702-704: "*where R is the carbon release rate (g C m^{-2} day^{-1}) in RTS-collapsed area, C_0 is the residual SOC stock (g m^{-2}) in collapsed areas, k_i and f_i are the decomposition coefficient and fractional proportion of the active and slow carbon pools in the two-pool model, respectively.*"

Lines 656-661: "*To calibrate the model, we integrated surface soil incubation data from collapsed area of the three alpine vegetation types (alpine swamp meadow, alpine meadow, and alpine steppe), with control area data adopted for the steppe type. Additionally, deep soil incubation data (30–100 cm) from published studies were incorporated*[38,72-74]*. Using Bayesian probability inversion and Markov chain Monte Carlo techniques*[75]*, we derived the initial parameter values and ranges for the active and slow carbon pools.*"

(2) A soil moisture constraint function (Tian et al.,2005), based on grassland ecosystem response curves (Peng et al.,2025), was integrated into the model to better reflect field conditions.

$$r_m = \begin{cases} \dfrac{\theta(\theta-1)}{\theta(\theta-1)-(\theta-\theta_{opt,\min})^2}, & \theta < \theta_{\text{opt,min}} \\ 1, & \theta_{\text{opt,min}} \leqslant \theta \leqslant \theta_{\text{opt,max}} \\ \dfrac{\theta(\theta-1)}{\theta(\theta-1)-(\theta-\theta_{opt,\max})^2}, & \theta > \theta_{\text{opt,max}} \end{cases}$$

Lines 672-675: "The effects of *soil moisture on carbon release were parameterized based on the grassland ecosystems response curve*[79] *and the soil moisture sensitivity framework from the Dynamic Land Ecosystem Model (DLEM) (Equation 5)*[80]."

Lines 709-712: "*r_m is the soil moisture regulation factor, θ is the volumetric soil water content (cm^3 cm^{-3}), $\theta_{opt,max}$ and $\theta_{opt,min}$ represent the upper and lower optimal soil moisture threshold for Reco. $SOCC_{con}$ and $SOCC_0$ were the SOC contents in control and collapsed areas, respectively.*"

(3) The model parameters were calibrated using monitored ecosystem respiration data from active RTSs. The proportion of heterotrophic respiration to ecosystem respiration was used to scale model outputs to observed ecosystem respiration. Subsequently, the calibrated model was validated against independent field monitoring data from 14 RTSs.

Lines 689-691: "*The two-pool model was then driven by field-measured SOC stocks, soil temperature and moisture data. Simulated outputs were validated against in-situ Reco to determine parameter scaling factors and estimate vegetation-specific ratios of heterotrophic*

respiration to Reco."

Lines 691-695: "*The calibrated two-pool model effectively reproduced CO_2 release rates in active RTSs (Supplementary Fig. 16). The model exhibited robust performance, with a coefficient of determination (R^2) of 0.915, a root mean square error (RMSE) of 0.915 g C m^{-2} d^{-1}, and a bias of -0.18 g C m^{-2} d^{-1} (Supplementary Fig. 16).*"

In summary, based on your valuable comments, we have systematically addressed the concerns regarding the methodology. These revisions ensure that our estimates of carbon release are both robust and process-realistic.

References:

Qin, S. et al. Temperature sensitivity of SOM decomposition governed by aggregate protection and microbial communities. Sci. Adv. 5, eaau1218 (2019).

Mu, M. et al. Decline of CO_2 release during the evolution of the thaw slump on the northern Qinghai‐Tibet Plateau. J GEOPHYS RES-BIOGEO. 129, e2024JG008162 (2024).

Zhao, Y. & Hu, X. Soil pore structure may affect microbial communities involved in carbon cycling during thaw slumps development. Appl. Soil Ecol. 206, 105821 (2025).

Li, Y. et al. Microbial community responses reduce soil carbon loss in Tibetan alpine grasslands under short‐term warming. Glob. Chang Biol. 25, 3438-3449 (2019).

Qin, S. et al. Temperature sensitivity of permafrost carbon release mediated by mineral and microbial properties. Sci. Adv. 7, eabe3596 (2021).

Tian, H. et al. DLEM-the dynamic land ecosystem model, user manual. The ESRA (Ecosystem Science and Regional Analysis) Laboratory, Auburn University, Auburn, AL (2005).

Peng, J. et al. Global Evidence of the Unimodal Response of Ecosystem Respiration to Soil Moisture. Adv. Sci. e09753 (2025).

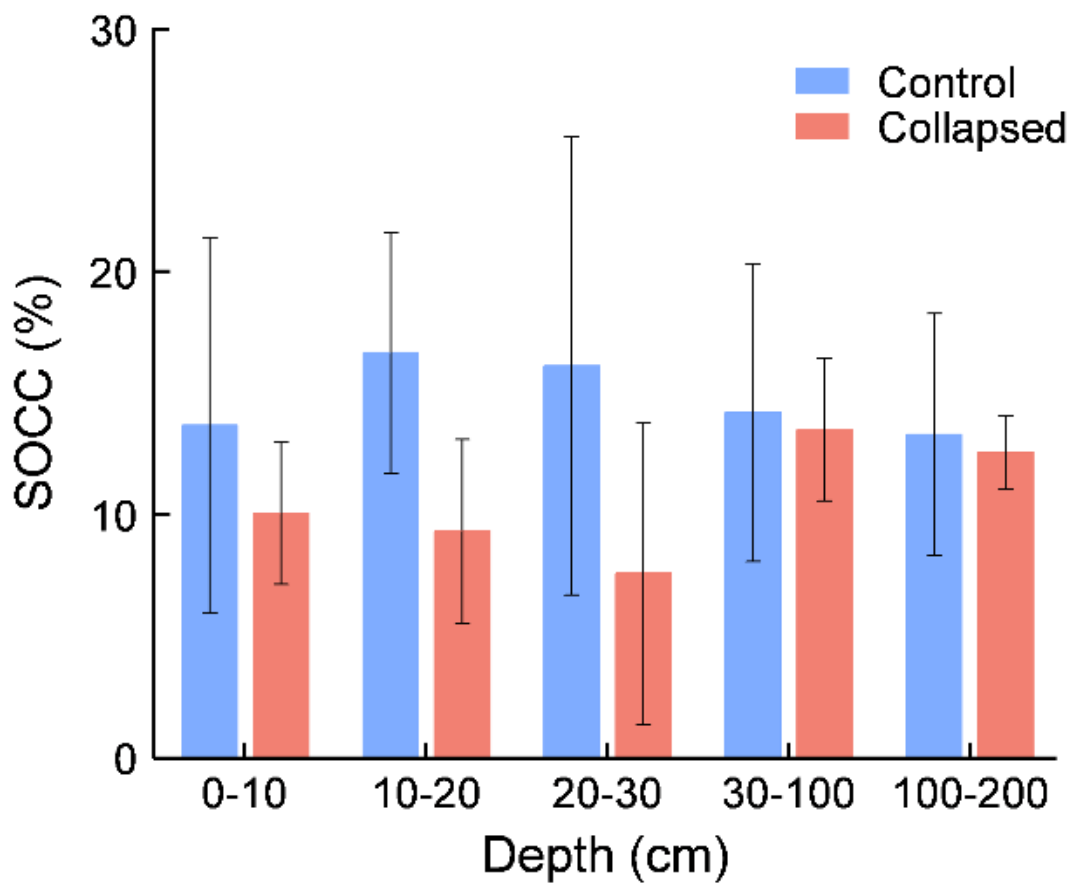

Supplementary Fig. 15 Soil organic carbon contents (SOCC) within the 0-200 cm depth in both control and collapsed areas of RTSs. *Error bars denote ±1 standard error (SE).*

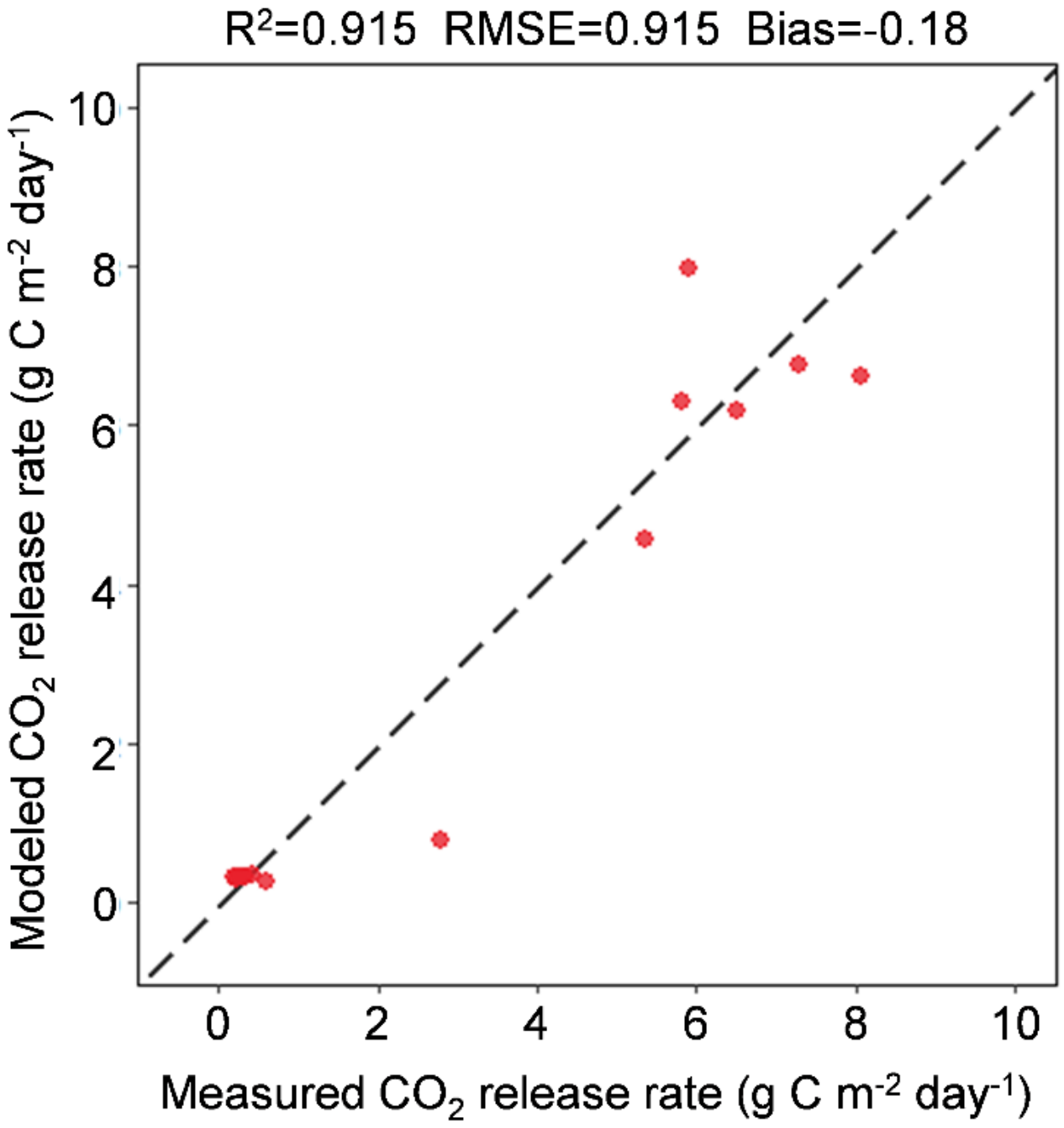

Supplementary Fig. 16 Correlations between modeled and measured CO_2 release rates. *The modeled CO_2 release rates were derived from the two-pool model. RMSE: Root Mean Square Error.*

Additionally, we have updated the Abstract and Results sections (Figures 4 and 5). All corresponding discussion in the manuscript has been enhanced as follows:

Lines 29-35: "*we estimate that CO_2 and CH_4 release from the expansion of RTSs increased 1.3-fold from 2017 to 2022. Projections indicate that the area of RTSs susceptibility will increase 15–18% by 2100. The corresponding CO_2 release rate is projected to reach 419 ± 316 g C m^{-2} yr^{-1}, roughly 1.5 times higher than the carbon uptake of alpine ecosystems on the plateau. Compared with gradual thaw, abrupt thaw will lead to a 22% increase in CO_2 release up to 2100. While the CO_2 and CH_4 release from increasing RTSs susceptibility areas will surge 1.2-fold relative to 2022.*"

Lines 213-223: "*To quantify the impact of RTS expansion on carbon release across the Tibetan Plateau permafrost region, we developed a two-pool decomposition model applicable to the RTSs through estimating the respective parameters of carbon decomposition under three vegetation types on the basis of 2,153 in-situ observations of CO_2 and CH_4 release rates as well as SOCC in the collapsed areas (Methods, Supplementary Table 1). The results indicate that the RTSs-induced mean CO_2 release rate was 137 ± 22 g C m^{-2} yr^{-1} during 2017-2022, and CH_4 release rate was 59 ± 4 g C-CO_2eq m^{-2} yr^{-1} (Supplementary Fig. 9), which account for approximately 39% of that caused by gradual permafrost thaw[33]. The estimated annual RTS-induced CO_2 release rate has a great range from 52 to 1160 g C m^{-2} yr^{-1} due to high variations in SOC stocks[43]. The frequency distribution exhibits 46% of the carbon release is concentrated between 100 and 150 g C m^{-2} yr^{-1} (Supplementary Fig. 10).*"

Lines 230-235: "*Driven by the expansion of RTSs across the Tibetan Plateau from 2017 to 2022[26], the corresponding CO_2 release increased from 3.1(95% confidence interval: 2.1-4.1) Gg C yr^{-1} to 7.1 (4.7-9.4) Gg C yr^{-1}. And CH_4 release surged from 0.02 (0.01-0.03) Gg C-CO_2eq yr^{-1} to 0.07 (0.06-0.08) Gg C-CO_2eq yr^{-1}. That is, expansion of RTSs during 2017–2022 leads to a 1.3-fold increase in CO_2 release and a 2.6-fold increase in CH_4 release (Fig. 4c,*

Supplementary Fig. 11c)."

Lines 255-259: "*The results indicate that annual CO_2 release rate from RTSs susceptibility will reach 257 ± 14 g C m^{-2} yr^{-1} under SSP2-4.5, and 362 ± 39 g C m^{-2} yr^{-1} under SSP5-8.5 by 2100 (Fig. 5a). Compared to the projections for gradual permafrost thaw on the basis of multiple models[32,33], abrupt thaw leads to a 22% increase in CO_2 release.*"

Lines 263-267: "*Nevertheless, by 2100, total carbon release from RTSs on the Tibetan Plateau are projected to reach 7.9 (5.3-10.5) Gg C-CO_2eq yr^{-1} under SSP1-1.9, 10.1 (6.8-13.4) Gg C-CO_2eq yr^{-1} under SSP2-4.5, and 16.1 (10.0-22.2) Gg C-CO_2eq yr^{-1} under SSP5-8.5 (Fig. 5c). Compared to 2022, RTSs will lead to a maximum increase of 1.3-fold on the plateau.*"

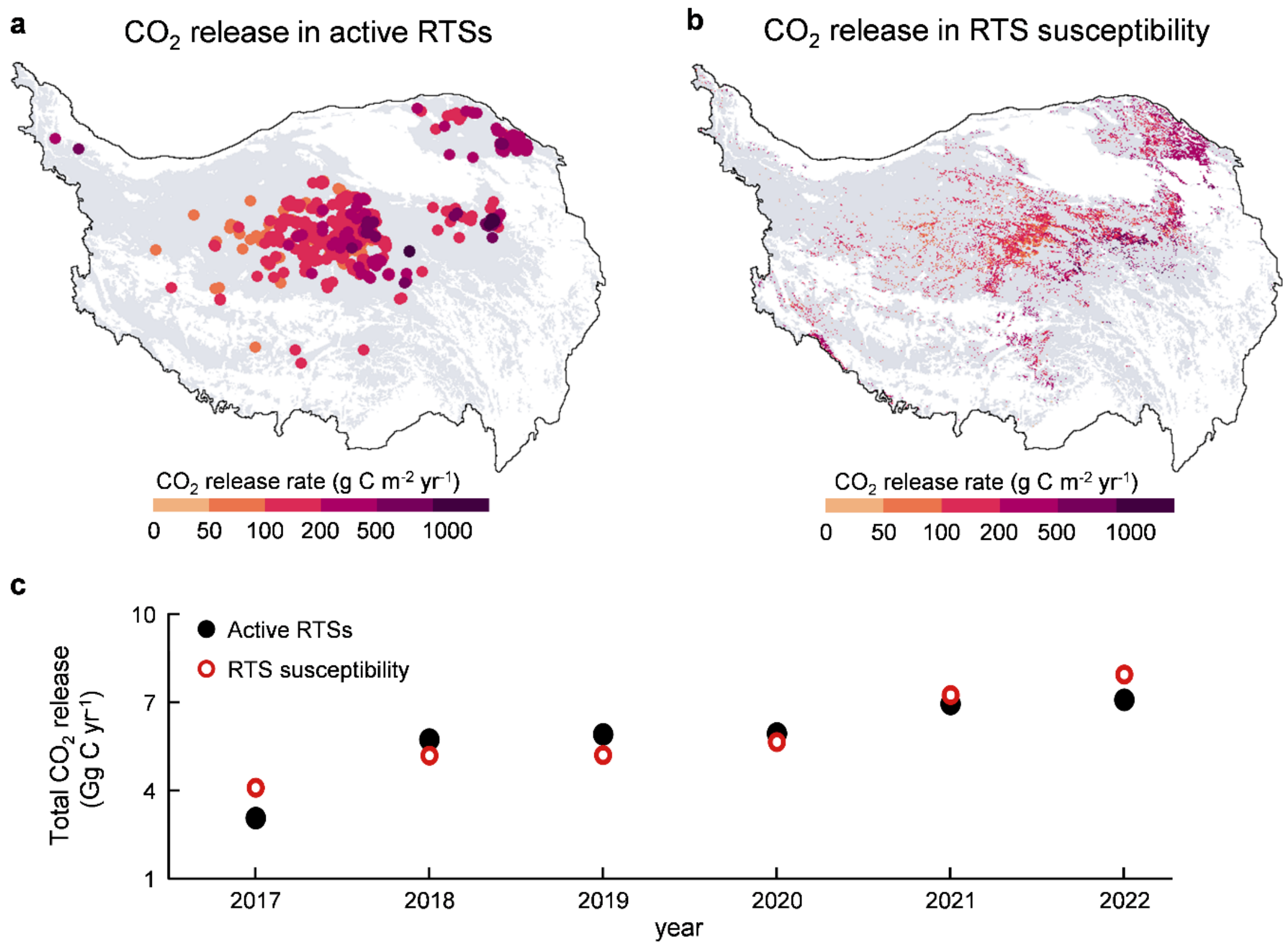

***Revised Fig. 4 | CO_2 release from active RTSs and susceptibility areas during 2017-2022. (a-b)** Spatial distribution of CO_2 release rate from active RTSs sites (a) and susceptibility areas (b) in 2022. (c) Annual changes in total CO_2 release from active RTS sites and susceptibility areas between 2017 and 2022.*

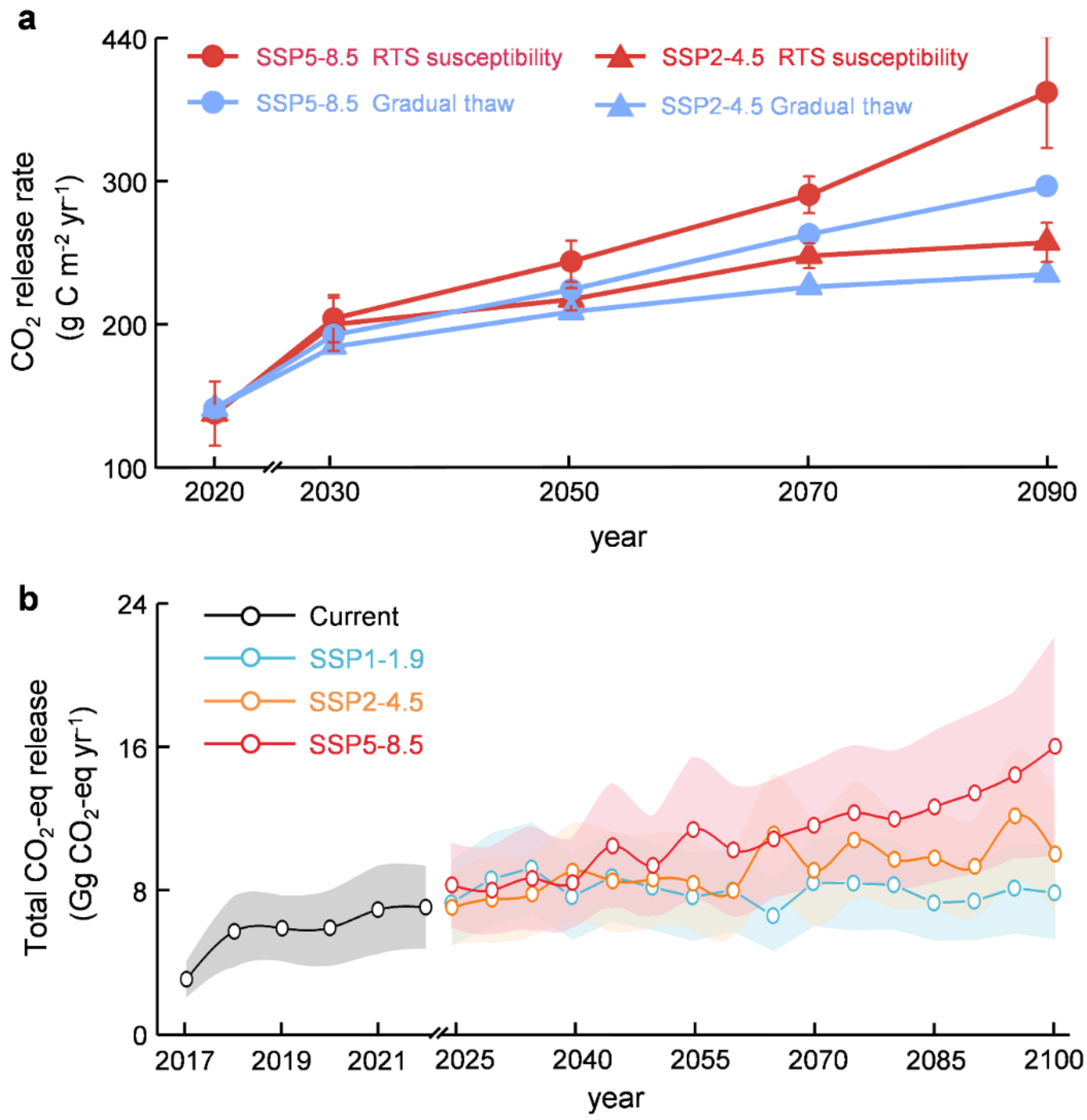

Revised Fig. 5 | Future projected carbon release from RTSs. (a) *Projected changes in annual CO_2 release rate in active RTSs and gradual permafrost thaw*[31] *on the Tibetan Plateau under SSP2-4.5 and SSP5-8.5 scenarios.* ***(b)*** *Projected total CO_2 and CH_4 release from the active RTSs under future climate scenarios. The shaded area presents the 95% confidence interval standard deviation derived from Monte Carlo simulations.*

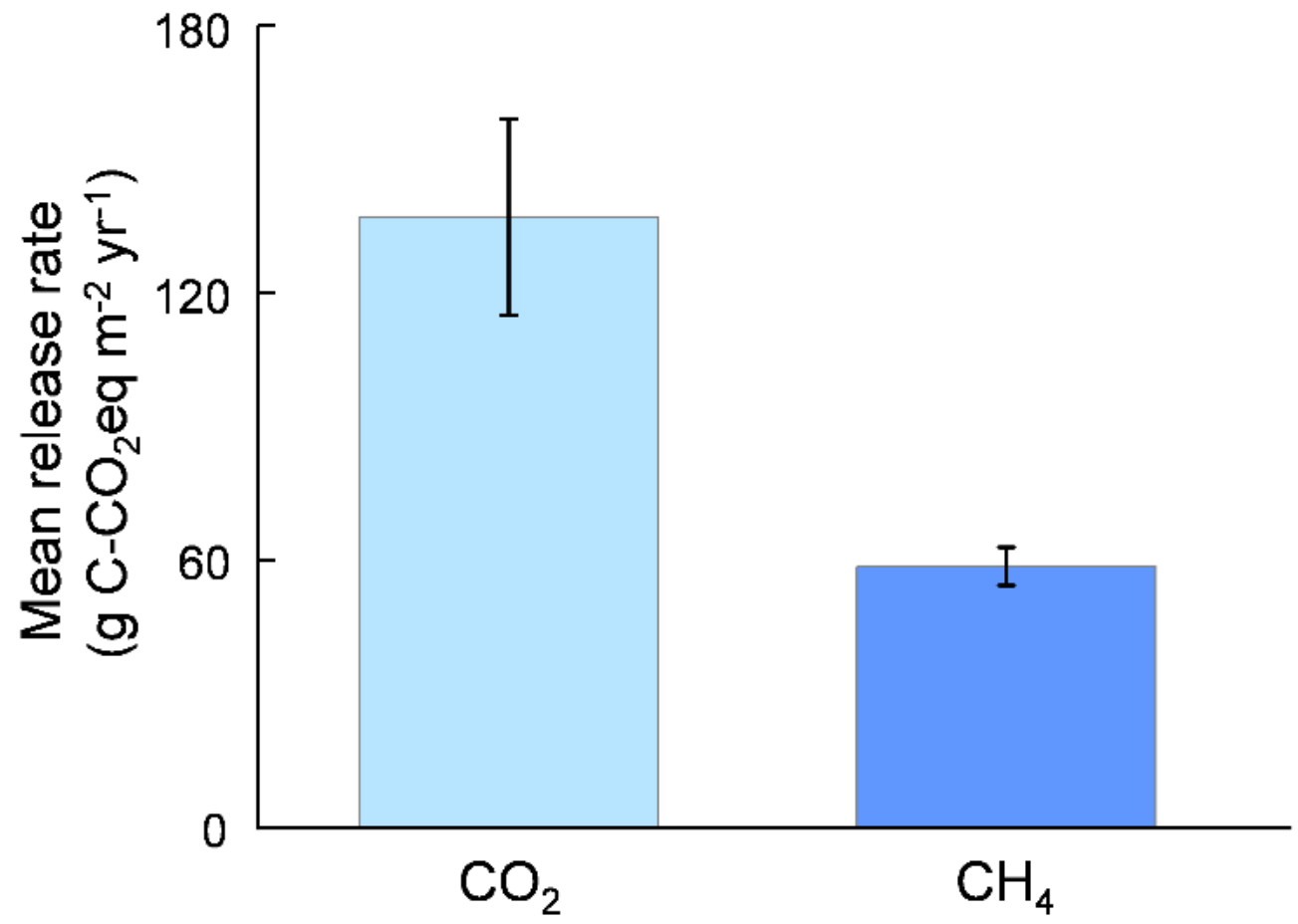

Supplementary Figure 9. Average CO_2 and CH_4 release rate from RTSs during 2017-2022.

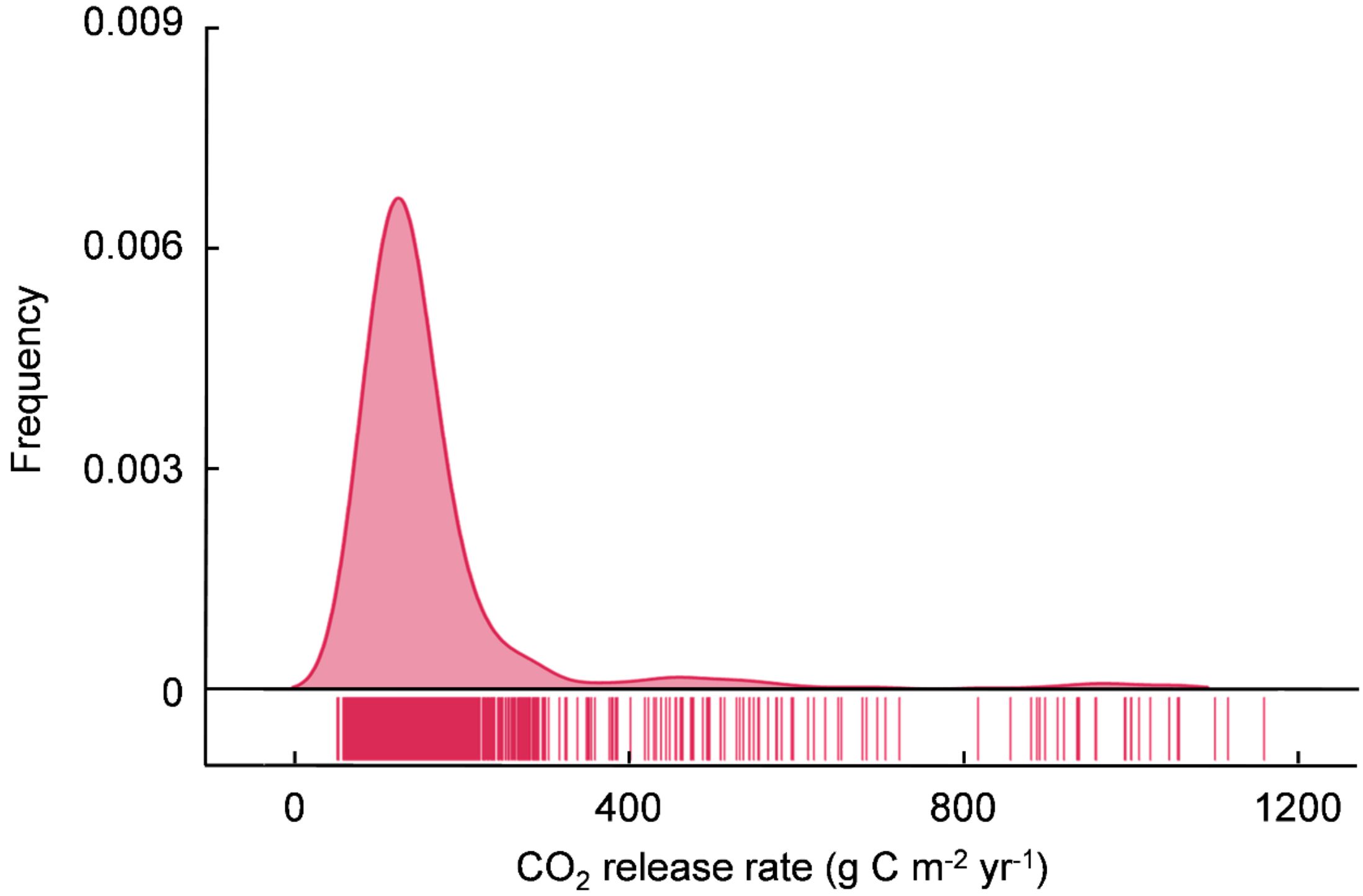

Supplementary Figure 10. Frequency distribution of CO_2 release rates from active RTSs on the Tibetan Plateau in 2022.

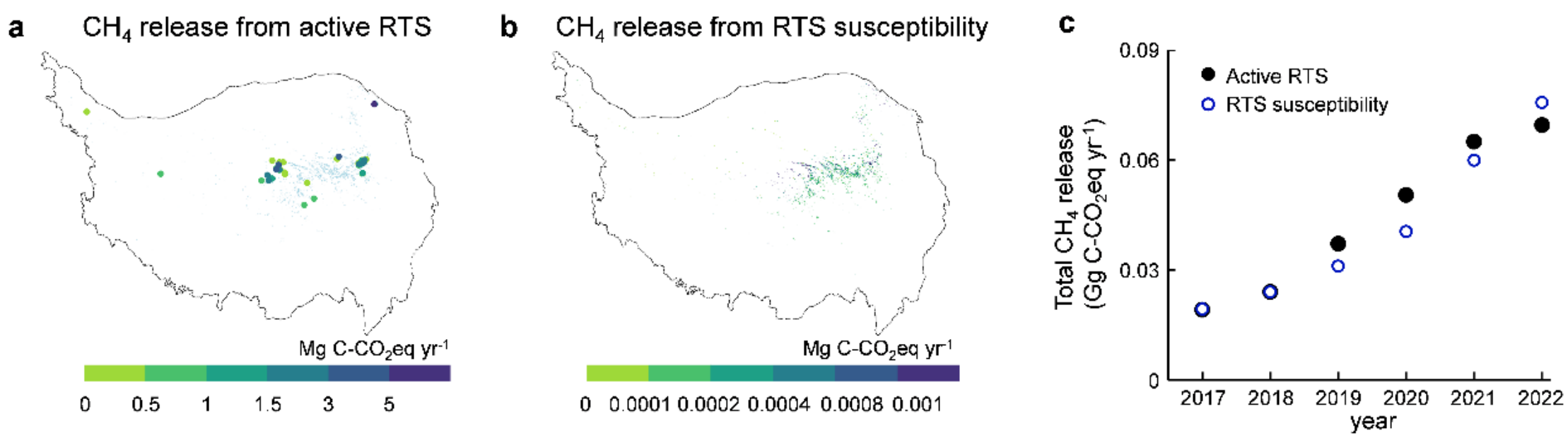

Supplementary Figure 11. CH_4 release from actual RTS sites and RTS susceptibility regions during 2017-2022. (a) *CH_4 release from active RTS sites.* ***(b)*** *CH_4 release from RTS susceptibility regions.* ***(c)*** *Total CH_4 release from both regions between 2017 and 2022.*

Specific comments:

LL494-498: It is not clear to me whether this processing was carried out as part of this study or whether it was done in the studies from which the inventories were obtained, i.e., refs 8,23? Could the authors clarify?

Response: Thanks for the detailed comment. The two datasets used herein are derived from our previous work. To streamline the methodology section, the relevant details of these datasets have been simplified by citing the corresponding publications.

*Peng, X. et al. The first hillslope thermokarst inventory for the permafrost region of the Qilian Mountains. Earth Syst. Sci. Data **16**, 2033-2045 (2024).*

*Xia, Z. et al. Widespread and rapid activities of retrogressive thaw slumps on the Qinghai-Tibet Plateau from 2016 to 2022. Geophys. Res. Lett. **51**, e2024GL109616 (2024).*

LL504-505ff: I am a bit puzzled that the SOC content discussed in the study is limited to depths of maximum 30 cm; thaw slumps actually expose headwalls up to several (tens of) meters deep. Furthermore, it is not very clear whether these are samples from the headwall or debris tongues, or elsewhere? Also, could the authors clarify the exact methodology for the measurements of GHG fluxes and Reco?

Response: We would like to express our sincere gratitude for your constructive comments, which have facilitated the improvement of our research by optimizing the methodologies employed.

1. Incorporating deep soil organic carbon (SOC)

Through analyzing the changes of SOC induced by RTS, it was found that the SOC content in the 30–200 cm layer of the collapsed area did not exhibit a significant difference compared with that of the control area (Supplementary Fig. 15). Thus, for the deep-layer carbon pool (30–100 cm), the SOC stocks from the corresponding depth in the control area were directly adopted as the initial value in this study. This revision was shown as follows:

Lines 683-687: "*Surface soil organic carbon content (SOCC, 0–30 cm) in collapsed areas of RTSs was estimated as the residual value after applying vegetation-specific and depth-specific loss rates derived from deep drilling data (Equations 6; Supplementary Fig. 2), whereas the deep SOCC (30-100 cm) was assumed to remain unchanged (Supplementary Figs. 15).*"

2. Adding sampling locations and greenhouse gas flux measurement methods

We have enhanced the Method by adding field sampling description: All samples and measurements within the collapsed areas were collected from vegetated drape zones and gently sloping exposed areas (including debris tongues). No sampling work was performed on unstable, active headwalls. In addition, greenhouse gas measurement methods were enhanced, specifically, Reco was measured using a Li-8100 soil carbon flux system with a dark hood. The CH_4 flux was determined using the static chamber sampling followed by gas chromatographic analysis.

Lines 521-527: "*To ensure standardized sampling design and data comparability, each RTS was partitioned into three functional distinct areas: (1) a control zone (an undisturbed permafrost area situated at least 5 meters away from the RTS boundary), (2) a drape zone (a vegetated area adjacent to the slump margin or distributed within the debris tongue), and (3) an exposed zone (vegetation-free bare soil areas). For each zone, a minimum of three replicate sampling points were systematically established (Supplementary Fig.12).*"

Lines 530-537: "*Ecosystem respiration (Reco) was measured during the growing seasons (April–October) from 2021 to 2023 on 17 RTSs using a Li-8100 automated soil CO_2 flux system (Li-Cor Inc., Lincoln, NE, USA) equipped with a shaded chamber. Each measurement lasted 3 minutes, providing direct estimates of CO_2 release rate along with concurrent soil temperature and moisture at 10 cm depth. Simultaneously, CH_4 release rates were monitored on four RTSs in alpine swamp meadows using a static closed-chamber technique. Gas samples (25 mL each) were collected from the chamber headspace at 0, 10, 20, and 30 minutes after closure and analyzed for CH_4 concentration by gas chromatography.*"

LL519-520: I don't understand the end of the sentence. Where can these monthly mean values

be found?

Response: We apologize for the confusion. The monthly mean data of carbon release have been provided in Supplementary Dataset S1. We have now explicitly cited this source at the end of the relevant sentence in the revised manuscript.

Lines 517-521: "*To investigate the impacts of active RTSs on carbon release, we conducted in-situ field monitoring at 43 active RTS sites across the Tibetan Plateau. These sites cover three dominant alpine vegetation types: alpine swamp meadow, alpine meadow, and alpine steppe. The monthly mean data of carbon release was shown in Supplementary Dataset S1. We monitored soil organic carbon content (SOCC), ecosystem respiration (Reco), and CH_4 release rate.*"

L525: I don't fully understand what Suppl. Tab. 3 shows.

Response: Thank you for your concern. The Suppl. Tab. 3 shows the annual counts of RTSs incidents used in model during 2016-2022 before and after spatial filtering. It has been revised in the updated version.

Lines 548-554: "*Prior to machine learning modeling, spatial filtering was applied to the initial RTSs inventory to mitigate spatial autocorrelation and sampling bias. ENMTools was employed to remove redundant records by retaining solely one RTS occurrence per grid cell. This process reduces overfitting and improves model generalizability by more precisely capturing the relationship between RTS occurrence and environmental conditions. The annual counts of RTSs incidents used in the model during 2016-2022, both before and after spatial filtering, are presented in Supplementary Table 3.*"

Supplementary Table 3. The annual counts of RTSs incidents used in machine learning model during 2016-2022 before and after spatial filtering.

Year	*Counts of RTS incidents before spatial filtering*	*Counts of RTS incidents after spatial filtering*
2016	*2,165*	*1,482*
2017	*3,424*	*2,129*
2018	*3,657*	*2,262*
2019	*4,495*	*2,721*
2020	*4,553*	*2,781*
2021	*4,706*	*2,864*
2022	*4,728*	*2,870*

L560: Can the authors define precisely what is meant by 'RTS susceptibility' in this paragraph and the previous one? Is it the area susceptible to being affected by an RTS? The portion of the area of one pixel?

Response: Thanks for your detailed suggestion. We have updated the relevant expressions in the Methods.

Lines 595-601: "*The RTS susceptibility distribution is represented as a relative probability index from 0 to 1, reflecting the probability of RTS occurrence (from low to very high) under various environmental conditions. Referring to the classification standard*[65], *RTS susceptibility was categorized into five levels: very low (0-0.2), low (0.2-0.4), moderate (0.4-0.6), high (0.6-0.8), and very high (0.8-1). Regions with a probability index below 0.2 were defined as control areas, whereas those with an index equal to or exceeding 0.2 were designated as RTSs-influenced areas.*"

L561: Can the authors define 'SHAP model'? the acronym?

Response: We have provided a description of SHAP model in the revised version.

Lines 609-628: "*To quantify the effects of environmental variables on RTS susceptibility, we conducted a two-step analytical framework, specifically, combining the SHapley Additive exPlanations (SHAP)algorithm and sensitivity analysis to disentangle their respective contributions and spatial heterogeneity. The SHAP algorithm utilizes Shapley values to quantitatively assess the marginal contribution of each feature to model outputs*[66]*, and was employed herein to holistically quantify the overall contribution of each environmental variable to RTS susceptibility. The sensitivity analysis was designed to identify the dominant driving factors of RTSs susceptibility at the pixel scale (1 × 1 km²), thereby capturing fine-grained spatial variations in the importance of influencing factors.*

Specifically, we applied the SHAP algorithm using the Python package "shap" to quantify the relative influence of various environmental variables on RTSs susceptibility across the entire study area. Further, based on our final random forest (RF) model, we performed a sensitivity analysis to identify primary drivers of RTSs susceptibility in the permafrost regions[67]*. This sensitivity analysis proceeded: first, we predicted RTSs susceptibility for each 1 × 1 km² pixel using the full set of environmental variables; second, we quantified the relative sensitivity of RTSs susceptibility to each variable by comparing the performance of the full RF model with that of "reduced models", each excluding one environmental variable at a time. For each pixel, the dominant driver was defined as the variable with the highest sensitivity value (⩾0.2). If no single variable met this threshold, the pixel was classified as being influenced by "co-drivers".*"

Suppl. Fig. 10: In terms of morphology, what is presented here resembles a thermo-erosional gully more than a thaw slump. If this type of structure is frequently repeated at the sites available in this study, I believe that the terminology should be adapted to refer instead to hillslope thermokarst structures, clearly specifying that this encompasses many different geomorphological structures. This is particularly important for the CO_2/CH_4 ratio, which is likely to vary rather strongly between thermo-erosional gullies and thaw slumps. I believe that adjustments should be made where necessary.

Response: Thank you for the valuable suggestion. For the entire Tibetan Plateau, the systematic RTS inventory was used in this study, our study focuses on the impacts of RTS

development. To avoid any confusion, we have deleted several thermo-erosional gullies located in the Qilian Mountains. The carbon flux monitoring for a representative RTS has been shown as follows:

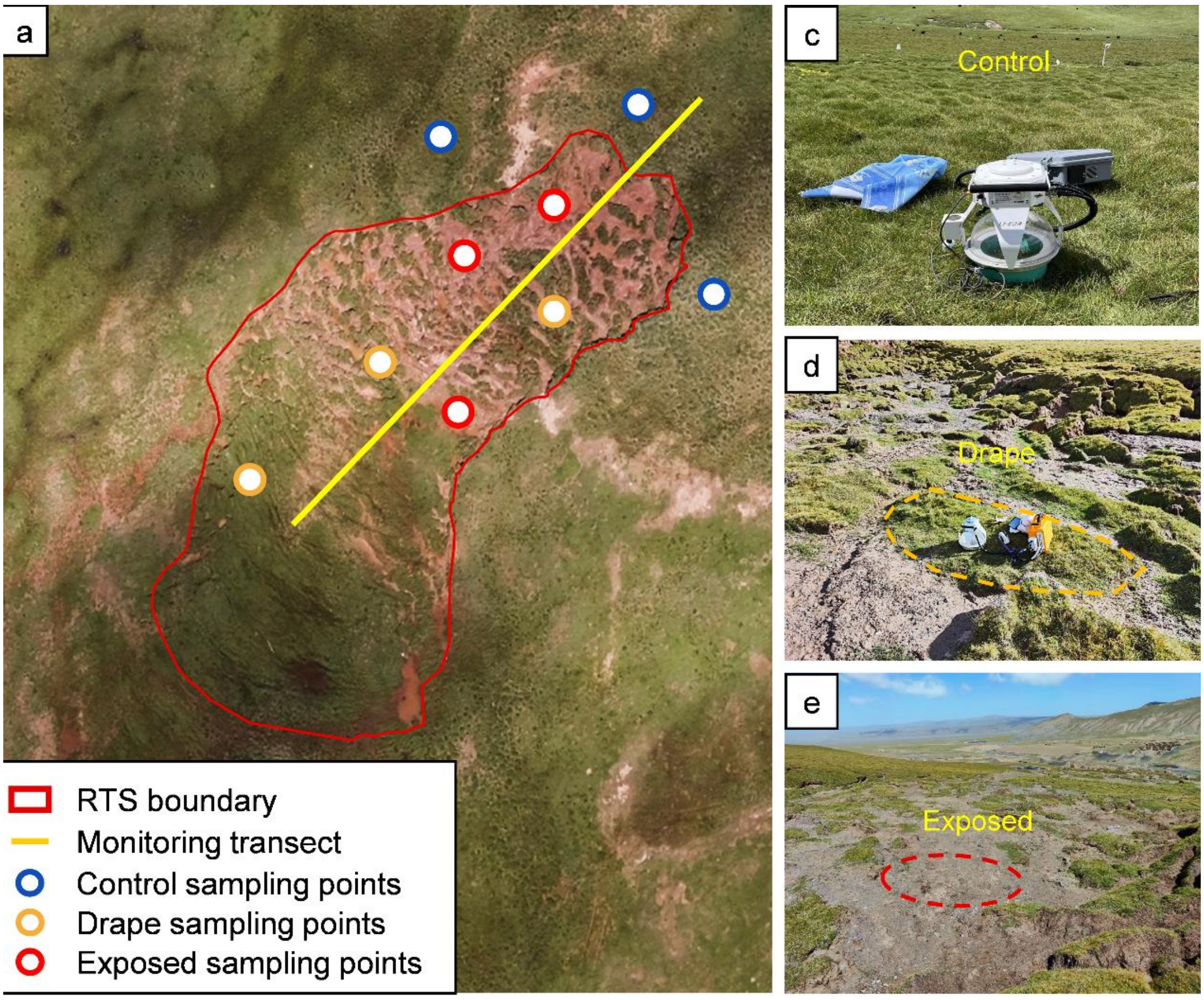

Supplementary Fig. 12 In-situ carbon monitoring sites within a representative RTS. *It schematically depicts the partitioning of the RTS into three distinct functional zones: control zone, drape zone, and exposed zone, which presents the typical layout of sampling points designed for gas flux and soil property measurements.*

Results and discussion

General comment:

With regard to my comments above on the assumptions of the model for carbon emissions, I am rather skeptical about the GHG emissions presented in this section.

Response: Thank you for your valuable and constructive comment. According to your above comments in our methods, we have revised the methods of "***Field observations and carbon release data***" (Lines 516-545) and "***Estimation of carbon release from active RTSs***" (Lines 650-716). Through recalculating the carbon release from active RTSs on the Tibetan Plateau, we enhanced the results and discussions in the revised manuscript. Below, we provide detailed responses to each of your specific comments.

Specific comments:

LL107ff: The observation regarding the increase in CH_4 flux seems surprising to me. Redox conditions following thaw slumping are considered to be oxic and relatively stable (e.g., Abbott & Jones (2015). Glob. Change Biol. 21, 4570–4587). Do the authors have any idea why they

are observing this increase? In the study by Yang et al. (2018) cited below, it would appear that these are somewhat specific conditions, and I am not sure that they can be generalized to the entire Tibetan Plateau.

Response: Thank you for your valuable comments. The observed increase in CH_4 release affected by RTSs was specific to alpine swamp meadows. This is attributable to the fact that high soil moisture in this area, along with continuous upslope water recharge, results in a proportionally smaller decline in water content within collapse areas (Supplementary Fig. 4b). The discussion in the main text has been revised as follow:

Lines 145-146: "*Notably, CH_4 flux data focus on the alpine swamp meadows.*"

Lines 112-114: "*In addition, CH_4 release rate induced by RTSs under alpine swamp meadow during growing seasons increases by 20.0%, rising from 0.78 (0.29–1.34) mg CH_4 m^{-2} d^{-1} in the control areas (non-collapsed) to 0.94 (0.29–1.70) mg CH_4 m^{-2} d^{-1} in the collapsed areas (Fig.1f).*"

Lines 122-135: "*The release of CO_2 and CH_4 from active RTSs is mainly driven by changes in soil hydrothermal conditions[16,34]. Soil temperatures in active RTS collapse areas are higher than those in control areas[35-37] (Supplementary Fig. 4a). This thermal anomaly, along with increased microbial diversity in collapsed areas, boosts the temperature sensitivity of CO_2 release[31,38,39], thereby intensifying heterotrophic respiration[38,40]. At the same time, reduced vegetation cover results in lower gross primary production (GPP)[34,41], turning RTS into stronger sources of carbon. Additionally, high soil moisture characteristic of alpine swamp meadows (Supplementary Fig. 4b), combined with continuous upslope water recharge in mountainous RTSs[39], causes only a slight decrease in soil moisture (~1.9%) in collapsed areas[41] (Supplementary Fig. 3). This maintains a favorable anaerobic environment, which promotes CH_4 release. Consequently, controlled by this integrated hydrothermal process, carbon release during the non-growing season exhibits no significant difference between the control and collapsed areas. Overall, this study offers solid observational evidence for effectively estimating carbon release from abrupt thaw in high-altitude permafrost regions.*"

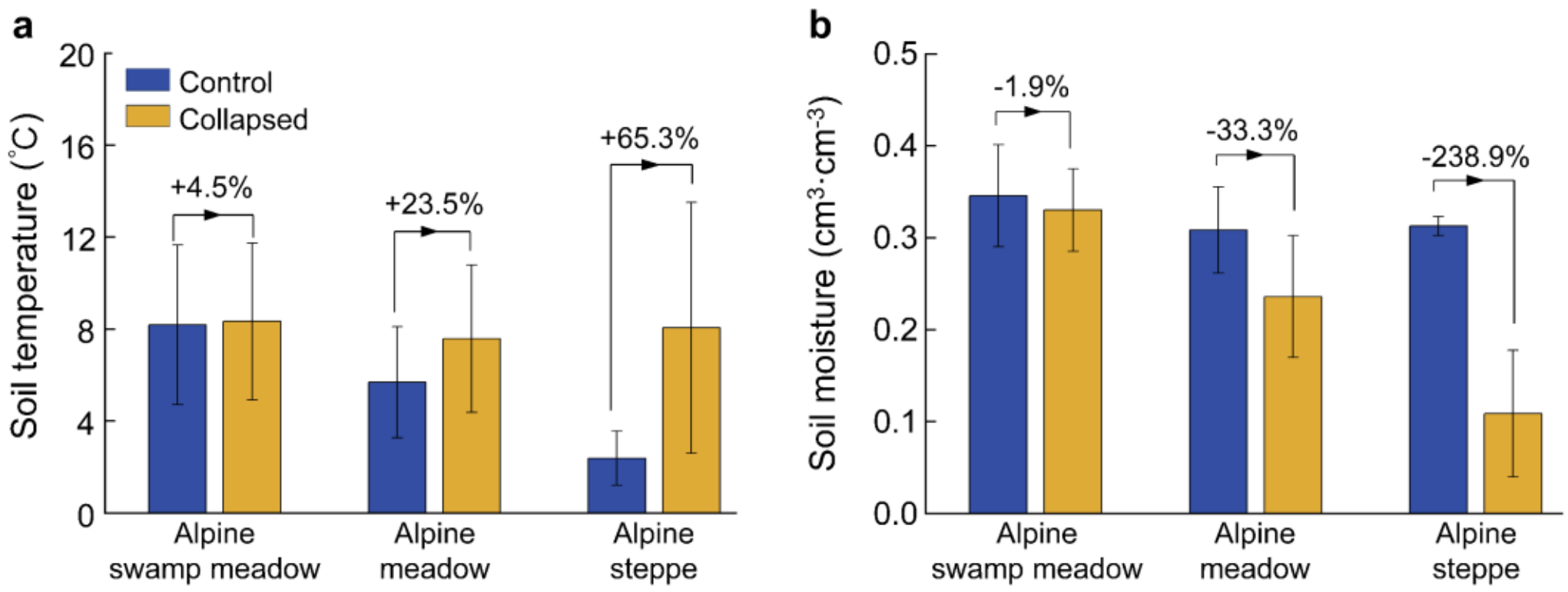

Supplementary Figure 4. Changes in soil temperature (a) and moisture (b) at 0–30 cm depth within control and collapsed areas of RTSs under different vegetation types during the growing seasons.

Fig. 1e-f-g: Are the increases and decreases pointed out significant?

Response: It has been added. "*Fig. 1 (e-g) Comparisons of the top 30 cm SOCC (e), CH_4 release rate (f) and ecosystem respiration (Reco) (g) between the control and collapsed areas*

of active RTSs during the growing season ($p > 0.05$)."

LL284-285: Are the 291 measurements of SOCC losses really observations? It's not very clear to me from the methodology.

Response: Yes, the measurements of SOCC losses are based on field observations. This dataset includes 145 measurements from our field campaign (processed into 67 depth-averaged values for the 0-30 cm layer) and 224 measurements obtained from the literature (*Supplementary Dataset S1*). These data represent SOCC from both control and collapsed areas of RTSs across three depth intervals (0–10, 10–20, 20–30 cm) on the Tibetan Plateau. We have clarified these methodological details in the revised Methods.

Lines 521-528: "*To ensure standardized sampling design and data comparability, each RTS was partitioned into three functional distinct areas: (1) a control zone (an undisturbed permafrost area situated at least 5 meters away from the RTS boundary), (2) a drape zone (a vegetated area adjacent to the slump margin or distributed within the debris tongue), and (3) an exposed zone (vegetation-free bare soil areas). For each zone, a minimum of three replicate sampling points were systematically established (Supplementary Fig.12). Total 145 soil samples were collected from three depth intervals (0-10 cm, 10-20 cm, 20-30 cm) at each site in this study.*"

Reviewer #2 (Remarks to the Author):

1)The authors clearly demonstrate that RTS expansion between 2016–2022 resulted in a 3.2-fold increase in CO_2 emissions and a 6.2-fold increase in CH_4 emissions. However, the manuscript would benefit from a more in-depth discussion of the processes driving these increases in carbon emissions.

Response: Thank you for this constructive suggestion. we have added the following discussion in the revised manuscript:

Lines 235-238: "*This shift was mainly driven by the expansion of active RTSs and increasing air temperatures[26,44]. Specifically, the rapid rise in new slumps was dominant during 2018–2019[26], while higher temperatures became the main controlling factor in 2021–2022[44].*"

2)Line112: The term meta-analyses may not be the most appropriate in this context; consider whether data synthesis would be more accurate.

Response: We have replaced "meta-analyses" with "data synthesis" in the revised manuscript.

Lines 76-78: "*To address the knowledge gap, we synthesized 4,728 RTS incidents, 1,878 in-situ CO_2 and CH_4 release rates measurements and 291 records of soil organic carbon content loss from 61 RTSs sites across the Tibetan Plateau (Fig. 1, Supplementary Table 1).*"

Lines 117-120: "*By synthesizing extensive observations, our study provides a systematic quantification for the changes in soil organic carbon content and carbon release affected by RTSs on the Tibetan Plateau.*"

Lines 683-688: "Surface soil organic carbon content (SOCC, 0 – 30 cm) in collapsed areas of RTSs was estimated as the residual value after applying vegetation-specific and depth-specific loss rates derived from data synthesis (Equations 6; Supplementary Fig. 2), whereas the deep SOCC (30-100 cm) was assumed to remain unchanged (Supplementary Figs. 15)".

3)Line 215: Please ensure consistent use of related terms such as slow warming, gradual thawing, and gradual permafrost thaw throughout the manuscript.

Response: The term has been standardized to "gradual thaw" in the revised manuscript.

4)The manuscript mentions the use of Q_{10}. Please provide additional explanation in the Methods section or Supplementary Materials to justify its selection and application.

Response: Thank you for the suggestion. We have added the following explanation in the Methods section to clarify the use of Q_{10}:

Lines 664-666: "*Soil temperature was treated as the primary driver of carbon release, which follows an exponential response to warming in the Tibetan Plateau permafrost regions. This relationship was expressed through a temperature sensitivity relationship (Q_{10})*[77] *(Equations 2 and 3).*"

5)The manuscript mentions the used of Monte Carlo simulations to estimate RTS-induced carbon release and its uncertainty. Could the authors clarify how this approach was specifically integrated into the carbon release calculations?

Response: Thank you for this suggestion. We have added the following clarification in the Methods:

Lines 697-701: "*We quantified uncertainty in annual carbon release estimates using 1,000 Monte Carlo simulations. Model parameters were randomly sampled from their calibrated ranges to compute total RTS-induced carbon release across the Tibetan Plateau in each run. The 95% confidence interval derived from the resulting 1,000 estimates represents the uncertainty range of our results.*"

6)Please specify the exact years and time span of the carbon monitoring datasets used. Including this information for all datasets will enhance the temporal representativeness of the simulation results.

Response: To clarify the temporal coverage of our carbon flux data, we have added the following sentence in the Methods:

Line 545: "*All field monitoring data cover the period from 2010 to 2023 (Supplementary Table 1).*"

Supplementary Table 1. Carbon release measurements in RTS on the Tibetan Plateau through data synthesis. Data marked with an asterisk (*) were used for validating the two-pool model.

References	*Number of RTS sites*	*Carbon measurement type*	*Monitored year*	*Control measurement count*	*Collapsed measurement count*
Xu et al., 2018[1]	*1*	*SOC*	*2015*	*3*	*3*
Liu et al., 2022[2]	*6*	*SOC*	*2014, 2020*	*40*	*55*
Mu et al., 2020[3]	*6*	*SOC*	*2016*	*6*	*12*

Wang et al., 2023[4]	*1*	*SOC*	*-*	*3*	*6*
Hu et al., 2023[5]	*1*	*SOC*	*2019*	*3*	*6*
Zhou et al., 2023[6]	*1*	*SOC*	*2021*	*18*	*30*
Ding et al., 2023[7]	*5*	*SOC*	*-*	*5*	*15*
Yang et al., 2018[8]	*1*	CH_4	*2016*	*2*	*6*
Wang et al., 2024[9]	*1*	CO_2	*2019, 2020*	*27*	*27*
Yang et al., 2021[10]	*1*	CO_2	*2017-2019*	*22*	*66*
Wang et al., 2022[11]	*1*	CO_2	*2018, 2019*	*29*	*87*
Mu et al., 2017[12]	*1*	CO_2	*2014-2016*	*12*	*24*
Mu et al., 2017[12]	*1*	CH_4	*2016*	*3*	*6*
*Mu et al., 2023**[13]	*7*	*SOC*	*2018*	*7*	*12*
*Mu et al., 2023**[13]	*7*	CO_2	*2018, 2020, 2021*	*20*	*39*
*Mu et al., 2023**[13]	*7*	CH_4	*2018, 2019, 2021*	*5*	*10*
*This study**	*35*	*SOC*	*2021, 2023*	*31*	*36*
*This study**	*13*	CO_2	*2021-2023*	*425*	*670*
*This study**	*4*	CH_4	*2021-2023*	*133*	*249*
SUM	*61*			*794*	*1359*

7)The Discussion section could be strengthened by elaborating on seasonal variations in carbon release from thermokarst, exploring driving factors such as microbial activity, soil moisture, and temperature. Additionally, please discuss potential mechanisms by which vegetation types influence thermokarst carbon release, for example through differences in carbon components such as lignin content.

Response: Thank you for the constructive suggestions. We have strengthened the Discussion section by adding the seasonal variations in carbon release. In addition, for the changes in CH_4 release caused by RTS, we have focused on the vegetation of alpine swamp meadow based on current observations.

Lines 122-133: "*The release of CO_2 and CH_4 from active RTSs is mainly driven by changes in soil hydrothermal conditions*[16,34]*. Soil temperatures in active RTS collapse areas*

are higher than those in control areas[35-37] (Supplementary Fig. 4a). This thermal anomaly, along with increased microbial diversity in collapsed areas, boosts the temperature sensitivity of CO_2 release[31,38,39], thereby intensifying heterotrophic respiration[38,40]. At the same time, reduced vegetation cover results in lower gross primary production (GPP)[34,41], turning RTS into stronger sources of carbon. Additionally, high soil moisture characteristic of alpine swamp meadows (Supplementary Fig. 4b), combined with continuous upslope water recharge in mountainous RTSs[39], causes only a slight decrease in soil moisture (~1.9%) in collapsed areas[41] (Supplementary Fig. 3). This maintains a favorable anaerobic environment, which promotes CH_4 release. Consequently, controlled by this integrated hydrothermal process, carbon release during the non-growing season exhibits no significant difference between the control and collapsed areas."

8)Line 112: When the term control area first appears in the manuscript, please provide a clear definition. Alternatively, consider replacing it with a more precise term, such as non-collapsed area, which corresponds more directly to collapsed area.

Response: We have clarified the term at its first occurrence in the revised manuscript as follows:

Lines 112-114: "*In addition, CH_4 release rate induced by RTSs during growing seasons increases by 20.0%, rising from 0.78 (0.29–1.34) mg CH_4 m^{-2} d^{-1} in the control areas (non-collapsed) to 0.94 (0.29–1.70) mg CH_4 m^{-2} d^{-1} in the collapsed areas (Fig. 1f).*"

9)Line 124–126: The text states that vegetation destruction reduces autotrophic respiration, yet also claims that CO_2 release rates in the subsidence area increase due to higher temperature sensitivity. The logical connection between these two points requires clarification. Please consider adding supporting data or citing relevant references; otherwise, the current phrasing may lead to contradictory interpretations.

Response: Thank you for the valuable comment. The corresponding discussion has been revised as follows

Lines 122-127: "*The release of CO_2 and CH_4 from active RTSs is mainly driven by changes in soil hydrothermal conditions[16,34]. Soil temperatures in active RTS collapse areas are higher than those in control areas[35-37] (Supplementary Fig. 4a). This thermal anomaly, along with increased microbial diversity in collapsed areas, boosts the temperature sensitivity of CO_2 release[31,38,39], thereby intensifying heterotrophic respiration[38,40]. At the same time, reduced vegetation cover results in lower gross primary production (GPP)[34,41], turning RTS into stronger sources of carbon.*"

10)Line 282: Please clarify what type of "uncertainties" are being referred to in this context.

Response: It has been revised as follows:

Lines 231-232: "*the corresponding CO_2 release increased from 3.1 (95% confidence interval: 2.1-4.1) Gg C yr^{-1} to 7.1 (4.7-9.4) Gg C yr^{-1}.*"

11)Line 338: Since RTS volume can influence carbon release, please add supporting references.

Response: Yes, the reference (Bernhard et al., 2022) has been added in the revised manuscript.

Lines 318-320: "*For instance, the volume of collapsed body changes dynamically during RTSs developing, affecting the real-time carbon release[54].*"

12)Line 111: When describing CH_4 flux in relation to RTS, it is preferable to consistently use terms such as induced or influenced throughout the manuscript for accuracy and consistency.

Response: The term has been standardized to "induced" throughout the manuscript.

Lines 112: "*In addition, CH_4 release rate induced by RTSs under alpine swamp meadow during growing seasons increases by 20.0%, rising from 0.78 (0.29–1.34) mg CH_4 m^{-2} d^{-1} in the control areas (non-collapsed) to 0.94 (0.29–1.70) mg CH_4 m^{-2} d^{-1} in the collapsed areas (Fig. 1f).*"

13)The integration of Shapley Additive Explanation (SHAP) analysis to interpret drivers of RTS susceptibility is innovative, The SHAP model may needs a few descriptive contents of description.

Response: Thank you for your great suggestion. We have provided a description of the SHAP model in the revised version.

Lines 609-622: "*To quantify the effects of environmental variables on RTS susceptibility, we conducted a two-step analytical framework, specifically, combining the SHapley Additive exPlanations (SHAP) algorithm and sensitivity analysis to disentangle their respective contributions and spatial heterogeneity. The SHAP algorithm utilizes Shapley values to quantitatively assess the marginal contribution of each feature to model outputs[66], and was employed herein to holistically quantify the overall contribution of each environmental variable to RTS susceptibility. The sensitivity analysis was designed to identify the dominant driving factors of RTSs susceptibility at the pixel scale (1 × 1 km²), thereby capturing fine-grained spatial variations in the importance of influencing factors.*

Specifically, we applied the SHAP algorithm using the Python package "shap" to quantify the relative influence of various environmental variables on RTSs susceptibility across the entire study area. Further, based on our final random forest (RF) model, we performed a sensitivity analysis to identify primary drivers of RTSs susceptibility in the permafrost regions[67]."

14)Methods: It is suggested to enrich the methods section, particularly, sensitivity analysis may need a few descriptive contents of description.

Response: Thank you for your great suggestion. We have provided a description of the sensitivity analysis in the revised version.

Lines 609-628: "*To quantify the effects of environmental variables on RTS susceptibility, we conducted a two-step analytical framework, specifically, combining the SHapley Additive exPlanations (SHAP) algorithm and sensitivity analysis to disentangle their respective contributions and spatial heterogeneity. The SHAP algorithm utilizes Shapley values to quantitatively assess the marginal contribution of each feature to model outputs[66], and was employed herein to holistically quantify the overall contribution of each environmental variable to RTS susceptibility. The sensitivity analysis was designed to identify the dominant driving factors of RTSs susceptibility at the pixel scale (1 × 1 km²), thereby capturing fine-grained spatial variations in the importance of influencing factors.*

Specifically, we applied the SHAP algorithm using the Python package "shap" to quantify the relative influence of various environmental variables on RTSs susceptibility across the entire study area. Further, based on our final random forest (RF) model, we performed a sensitivity analysis to identify primary drivers of RTSs susceptibility in the permafrost regions[67]. This sensitivity analysis proceeded: first, we predicted RTSs susceptibility for each 1 × 1 km² pixel

using the full set of environmental variables; second, we quantified the relative sensitivity of RTSs susceptibility to each variable by comparing the performance of the full RF model with that of "reduced models", each excluding one environmental variable at a time. For each pixel, the dominant driver was defined as the variable with the highest sensitivity value (⩾0.2). If no single variable met this threshold, the pixel was classified as being influenced by "co-drivers"."

15)It is, to my knowledge, the first study to provide comprehensive simulations of future RTS emissions across the QTP using SSPs. The author should explain why these three climate scenarios were chosen.

Response: We thank the reviewer for their positive feedback on our work. We have added the information in the revised manuscript.

Lines 642-648: "*Three scenarios (SSP1-1.9, SSP2-4.5, SSP5-8.5) were used to explore future changes of RTSs susceptibility by 2100. SSP1-1.9 is a combination of low societal vulnerability and a low forcing level, with a substantial change in land use. SSP2-4.5 is a combination of intermediate societal vulnerability and an intermediate forcing level. SSP5-8.5 combines high societal vulnerability and a high forcing level. By employing these three representative scenarios—spanning low, medium, and high levels of forcing and vulnerability—this study captures a broad spectrum of potential climate policy outcomes.*"

16)Line 522: This part of the content should describe the collection and processing of data, the title should be Climate and environmental variables.

Response: Thank you for your careful suggestions. The title has been changed to "Climate and environmental variables" in the revised manuscript.

17)RTS susceptibility mapping and Drivers and uncertainty of RTSs susceptibility should be further sorted out.

Response: We have rewritten this part in the revised manuscript.

Machine learning models and uncertainty analysis

Lines 572-585: *"Machine learning algorithms has rapidly advanced in dynamic modeling of permafrost landforms[59-61]. They have been employed to assess the susceptibility of various hazards[59,60], including RTSs dynamics at local and regional level[62-65]. In this study, a total of 9 individual models and one ensemble model were used for RTSs susceptibility mapping: Random Forest (RF), Generalized Linear Model (GLM), Generalized Additive Model (GAM), Generalized Boosted Regression Model (GBM), Classification and Regression Tree Analysis (CTA), Artificial Neural Network (ANN), Flexible Discriminant Analysis (FDA), Multivariate Adaptive Regression Splines (MARS), and Maximum Entropy Model (MaxEnt). We used the Receiver Operating Characteristic (ROC), Cohen's kappa index (Kappa), and the True Skill Statistics (TSS) to evaluate the models' performance. Based on the evaluation results, we finally selected four best-performing models (RF, GLM, CTA, and GBM) to construct an ensemble model. Consequently, we re-evaluated the performance of all ten models, showing that the RF model outperformed (AUC=0.963; KAPPA=0.822; TSS=0.823) the others (Supplementary Table 5).*"

Lines 587-606: "*The spatial distribution of RTS susceptibility was simulated using the BIOMOD_Projection function integrated within the biomod2 package. This function deploys*

the pre-trained ensemble model to environmental raster layers, enabling the prediction of RTS occurrence probability for each individual pixel across the study area. The resulting output is a georeferenced raster dataset, where each cell value corresponds to the ensemble-averaged weighted probability of RTS susceptibility, with values ranging from 0 (very low) to 1 (very high). For the Random Forest (RF), 50 independent runs were conducted; in each run, the dataset was randomly partitioned into 75% training data and 25% testing data. The final RTS susceptibility map was generated by averaging the results from all 50 outcomes. The RTS susceptibility distribution is represented as a relative probability index from 0 to 1, reflecting the probability of RTS occurrence (from low to very high) under various environmental conditions. Referring to the classification standard[65], RTS susceptibility was categorized into five levels: very low (0-0.2), low (0.2-0.4), moderate (0.4-0.6), high (0.6-0.8), and very high (0.8-1). Regions with a probability index below 0.2 were defined as control areas, whereas those with an index equal to or exceeding 0.2 were designated as RTSs-influenced areas.

To evaluate model uncertainty, we calculated the standard deviation (SD) and coefficient of variation (CV) across the 50 susceptibility simulations for each cell. The CV was used to quantify simulation uncertainty, with higher values indicating greater uncertainty in RTS susceptibility mapping (Supplementary Fig. 7)."

Drivers of RTSs susceptibility

Lines 609-617: *"To quantify the effects of environmental variables on RTS susceptibility, we conducted a two-step analytical framework, specifically, combining the SHapley Additive exPlanations (SHAP) algorithm and sensitivity analysis to disentangle their respective contributions and spatial heterogeneity. The SHAP algorithm utilizes Shapley values to quantitatively assess the marginal contribution of each feature to model outputs[66], and was employed herein to holistically quantify the overall contribution of each environmental variable to RTS susceptibility. The sensitivity analysis was designed to identify the dominant driving factors of RTSs susceptibility at the pixel scale (1 × 1 km²), thereby capturing fine-grained spatial variations in the importance of influencing factors."*

Lines 619-628: "*Specifically, we applied the SHAP algorithm using the Python package "shap" to quantify the relative influence of various environmental variables on RTSs susceptibility across the entire study area. Further, based on our final random forest (RF) model, we performed a sensitivity analysis to identify primary drivers of RTSs susceptibility in the permafrost regions[67]. This sensitivity analysis proceeded: first, we predicted RTSs susceptibility for each 1 × 1 km² pixel using the full set of environmental variables; second, we quantified the relative sensitivity of RTSs susceptibility to each variable by comparing the performance of the full RF model with that of "reduced models", each excluding one environmental variable at a time. For each pixel, the dominant driver was defined as the variable with the highest sensitivity value (⩾0.2). If no single variable met this threshold, the pixel was classified as being influenced by "co-drivers".*"

18)The availability of code and data provided in the Machine Learning Checklist should be clearly stated in the text.

Response: We have supplemented Data availability and Code availability in the revised manuscript.

Software used for the analysis

Lines 338-342: *For data analysis and organization, we used R version 4.2.2 (available at https:// www.R-project.org/), resampling and cropping, etc. were done using python version 3.11 (available at https://www.python.org/), model construction, importance ranking and visualization were done using R statistical software. GIS processing and presentation was done using ArcMap 10.8. (available at https://www.esri.com/en-us/arcgis/ products/arcgis-desktop/overview)*

Data availability

Lines 345-347: *Source Data are provided with this paper. All data supporting the f indings are available in the Figshare data repository (https://figshare.com/s/73a84b61124c0d10d0aa) and Supplementary Information. Source data are provided with this paper.*

Code availability

Line 350: *The supporting code is provided at Figshare (https://figshare.com/s/a09b8262eeed2b34c237)*"

We sincerely appreciate the reviewers' insightful feedback, which has significantly improved our manuscript. We hope our revisions adequately address all concerns.

REVIEWER COMMENTS

Response to Reviewers of NCOMMS-25-60807A

Reviewer #2 (Remarks to the Author):

The novelty and key improvements are as follows:

1.Clarity of Simulation Scope: The refined focus on "active RTS" appropriately mitigated the risk of overestimation inherent indelimitation extrapolating from active features to all RTSs. Equally important was the clear of CH_4 simulations to waterlogged alpine swamp meadows, which appropriately bounded the analysis and enhanced interpretability.

2.Model Framework Advancement: A key improvement noted in the Methods section was the replacement of the single-pool model with a two-pool framework. This was a significant and appropriate upgrade, as the two-pool model is the most widely applied and suitable framework for simulating carbon release in permafrost regions, as it better represents distinct carbon decomposition dynamics. This revision was directly substantiated by the notably lower carbon release estimates in the updated results, which clearly demonstrated the new model's effectiveness in constraining the previous overestimation.

3.Mechanistic Refinement: The integration of deep-soil decomposition processes represented a critical refinement. Importantly, the model optimization utilized incubation data from various vegetation types across the RTS area, incorporating temperature and moisture response functions to mechanistically constrain carbon release.

Collectively, these revisions, along with a notably deepened discussion of the results in response to reviewer comments, markedly improved the manuscript's methodological rigor, scientific coherence, and overall impact. I have only one minor editorial note: please ensure the consistency of figure and panel labeling throughout the manuscript, as panel "b" appears to be missing in Supplementary Figure 12. Beyond this, I have no further questions or concerns regarding this revised version.

Response: We sincerely appreciate your positive assessment of our study and your recognition of the novelty of our work.

Following your minor comment, we have carefully checked all figure and panel labels throughout the manuscript. The missing panel label "b" in Supplementary Figure 12 has been corrected in the revised version.

We thank you again for your thorough and careful review.

Reviewer #4 (Remarks to the Author):

This study estimates CO_2 and CH_4 emissions induced by retrogressive thaw slumps (RTSs) and develops models to project future changes in these gases under different climatic conditions. Overall, the manuscript is well written and presents interesting results. The findings highlight the importance of permafrost protection and relevant carbon management in permafrost-affected regions.

I have the following comments, which I hope will help further improve the manuscript:

Response: We appreciate your recognition of our work on permafrost carbon emissions, especially in regions affected by permafrost degradation. Your detailed and constructive comments provide valuable guidance for further improving the manuscript. We have thoroughly revised the paper to address all your specific concerns, particularly we have updated the method used for estimating carbon release. Below please find our point-to-point response.

I understand that the manuscript focuses on carbon release in terms of CO_2 and CH_4. However, it is important to recognize that the overall soil carbon balance is more relevant. This limitation should be clearly acknowledged. Since RTSs are in the early stages of

vegetation succession, any claims regarding whether they function as carbon sources or sinks should be made with caution. Without quantifying carbon inputs, conclusions about source–sink dynamics are not sufficiently supported (e.g., line 261).

Response: Thank you for your valuable comment. Following your suggestion, we have removed the relevant statements concerning carbon source and sink, as our estimates only focus on CO_2 and CH_4 emissions from RTS-affected areas. In addition, we have acknowledged this limitation in the Implications section of the revised manuscript.

Lines 320-335: "*Second, the developmental stage of RTSs was not incorporated into our estimation of carbon loss. As RTSs evolve, surface soil organic carbon loss tends to increase during early developmental stages and subsequently decreases*[18]*. In addition, RTSs development can cause substantial displacement and accumulation of deep soils*[13]*, influencing soil porosity and microbial communities*[49] *and thereby modulating carbon release. Consequently, there is an increasing need for future work to develop integrated carbon process models that account for the multi-factorial dynamics of abrupt thaw. Given the spatial heterogeneity in ground ice distribution*[50]*, the development of some RTSs may gradually stabilize, potentially enabling slow vegetation regrowth in affected areas*[34]*, which could further influence regional carbon source-sink dynamics*[51,52]*. Furthermore, the three-dimensional complexity of abrupt thaw necessitates a holistic framework to quantify carbon release across the entire process of RTS development. For instance, the volume of the collapsed body changes dynamically throughout RTS evolution, directly influencing real-time carbon release fluxes*[53]*. Improving process-based simulations of RTS-induced carbon release on the Tibetan Plateau can provide valuable insights for other mountain permafrost regions to systematically estimate permafrost carbon-climate feedbacks.*"

In permafrost-affected regions, permafrost depth is a critical variable influencing thaw dynamics. This parameter likely has significant effects on the spatiotemporal patterns of CO_2 and CH_4 emissions and should therefore be considered in the driver analysis.

Response: Thank you for this valuable comment. We fully agree that permafrost conditions are a critical factor influencing RTS development, thereby exerting an important impact on CO_2 and CH_4 emissions.

Following your suggestion, we have incorporated active layer thickness (ALT) as an additional permafrost-related variable in the analysis of RTS susceptibility. ALT is widely used as a reliable proxy for near-surface permafrost thermal state and degradation status. Corresponding revisions have been made in the Methods and Results sections of the revised manuscript.

Lines 158-187: "***Drivers of RTS susceptibility***"

"*Furthermore, to quantify the impact of RTSs on carbon release, we evaluated the environmental controls governing the spatial distribution of RTS susceptibility, including climate, topography, permafrost conditions, human activity and soil properties (Methods; Supplementary Fig. 6). Our results indicate that climate and topography exert dominant controls, with mean annual precipitation being the most important predictor, explaining 25.2% of the total variance in RTS susceptibility (Fig. 2a). Rising air temperatures accelerate permafrost degradation and ground-ice thaw, thereby weakening slope stability and preconditioning ice-rich slopes for failure*[40,41]*. In addition, increased precipitation saturates soils under favorable topographic settings, elevates pore-water pressure and reduces shear strength, ultimately triggering RTS initiation under gravitational forcing*[9]*. Particularly, extreme rainfall is a major driver of permafrost change and can accelerate active-layer thaw*[42]*. Analogous precipitation-mediated controls on RTS development have also been documented in Arctic permafrost regions*[9,12]*.*

Nonlinear relationships were identified between RTS susceptibility and each individual

environmental variable (Supplementary Fig. 7). Specifically, RTS susceptibility reached a maximum in regions with a mean annual precipitation of 300–400 mm, consistent with the observed spatial distribution of RTSs across the plateau[43]. Notably, the dominant drivers of RTS susceptibility vary substantially across different mountainous regions of the Tibetan Plateau (Fig. 2b). For instance, climatic conditions exert the primary control over RTS susceptibility in relatively arid zones such as the Karakoram and Himalaya. In contrast, topographic factors dominate in the Qilian Mountains, Kunlun Mountains and Pamir Plateau, presumably because they modulate terrain conditions conducive to the formation and preservation of ground ice[8]. In Inner Tibet, the main region of RTS occurrence, susceptibility is jointly modulated by multiple environmental factors. Collectively, RTS susceptibility across the Tibetan Plateau is strongly co-regulated, with interactive effects of multiple drivers accounting for 33.4% of the total area (Fig. 2c). Unlike previous studies, our analysis reveals the spatially contributions of various drivers across the Tibetan Plateau. Characterizing the spatial heterogeneity of these controlling factors is essential for robust assessment of RTS susceptibility in high-altitude permafrost environments."

Lines 574-578: "*Finally, we selected 10 environmental variables, including topography (elevation, slope, aspect), climate (mean annual precipitation, mean annual air temperature), permafrost conditions (active layer thickness, ground ice content), soil properties (silt, sand content), and human activity (human influence index).*"

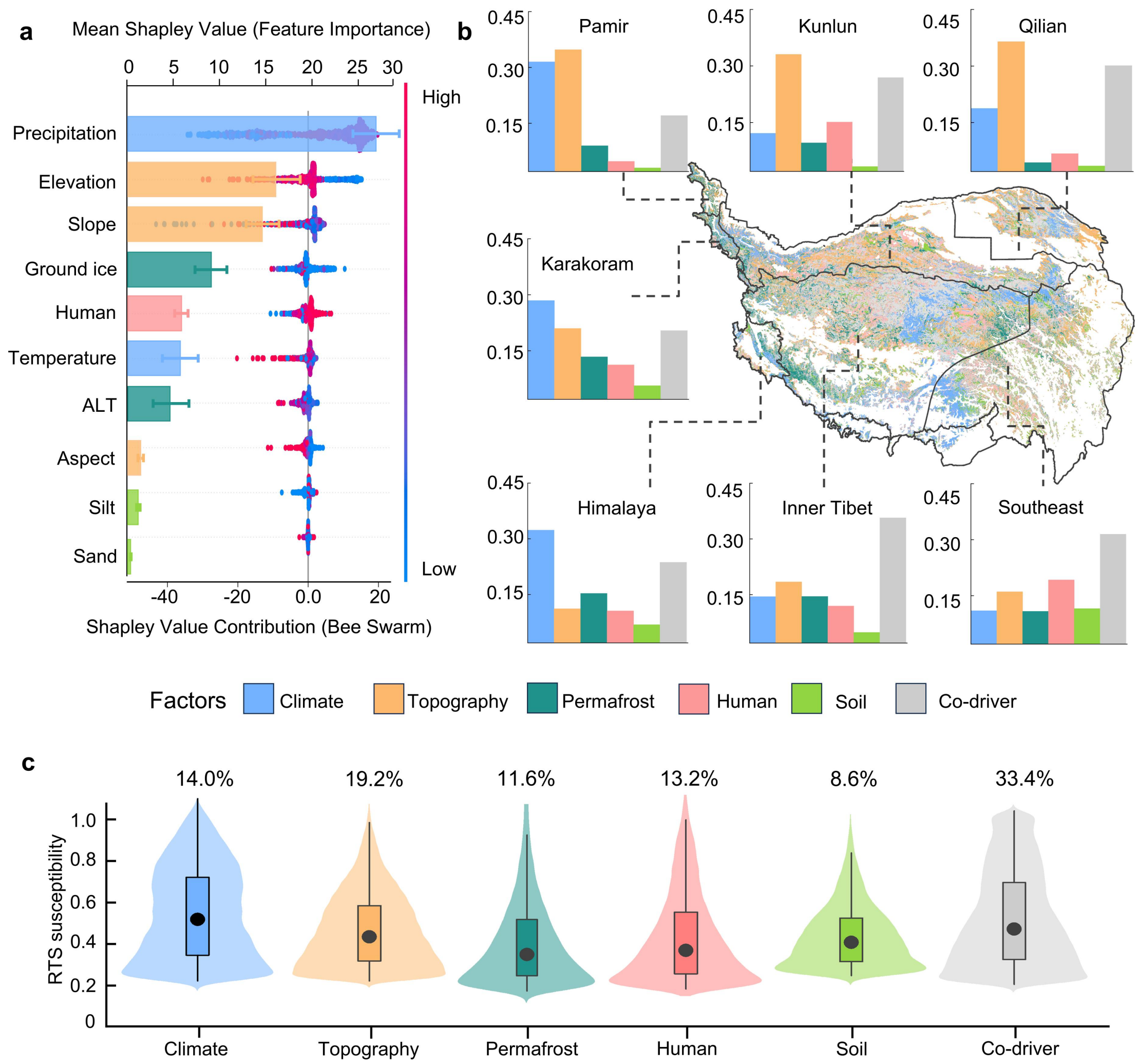

***Fig. 2 | Dominant drivers of RTS susceptibility. (a)** Relative importance of environmental variables*

*(climate, topography, permafrost conditions, human activity and soil properties) controlling RTS susceptibility derived from the SHAP analysis. Horizontal bars represent the relative contribution of each environmental variable. ALT denotes the active layer thickness of permafrost. (**b**) Spatial distribution of dominant driver categories. The co-driver category denotes regions where RTSs are jointly modulated by all five drivers. Inset bar charts shows the area proportion governed by each dominant driver across different mountainous regions. (**c**) Violin plots depicting the probability density of RTS susceptibility per km^2 across distinct dominant driver categories. Internal boxes indicate the median and interquartile ranges, with percentages representing the areal fraction corresponding to each driver.*

It would also be valuable to include results or discussion on the drivers of RTS changes during the study period. In particular, it is unclear why precipitation, rather than temperature, is identified as the most important predictor of RTS distribution. This appears inconsistent with statements elsewhere in the manuscript (e.g., line 237), where higher temperatures are described as the main controlling factor. Please carefully review the analysis and clarify this point, or better contextualize the apparent discrepancy. A key question is whether increases in RTSs are driven primarily by rising temperature, increasing precipitation, or a combination of both.

Response: Thank you for this insightful comment. We have rewritten the section of "***Drivers of RTS susceptibility***" (Lines 158-187), and clarified the corresponding discussion about carbon release to avoid any confusion.

Future projections constitute a substantial portion of the manuscript (as also noted by a previous reviewer). However, the two-pool carbon model used does not appear to include carbon inputs or transformations among pools. This structure may be overly simplistic for in situ predictions. Such models are typically more appropriate for incubation studies. When conducting projections, model structure is a critical consideration—often more important than parameter uncertainty, which has already been thoroughly quantified in this study. My suggestion is to drop this part and just use the RF model

Response: We agree your constructive comment. We have updated methodological framework by multiplying the observed CO_2 and CH_4 release rates across different vegetation types by the RTS susceptibility areas predicted by the RF model. This approach avoids the structural limitations of process-based carbon models and relies directly on field-observed carbon release fluxes. Accordingly, we have revised the corresponding content in the Methods and Results sections.

Lines 670-681: "*Estimation of carbon release from RTSs*"

"We quantified the total regional carbon release from three vegetation types (derived from monitoring data) by multiplying their mean carbon release rates by the area of RTS susceptibility[72], where high and very high susceptibility classes were defined as the RTS-affected region. An area density ratio was used to represent the actual RTS-affected area within each vegetation type, and uncertainties were propagated from the standard errors of both carbon release rate and area density estimates. Potential overestimation was corrected using the mean RTS area density for each vegetation type over the 2016-2022 period (Equation 1; Supplementary Table 6). The reliability of this susceptibility-based approach was validated by comparing its estimated annual carbon emissions with those derived from actual RTSs during the same period (Supplementary Fig. 10). The validated framework was subsequently applied to project future RTS-driven carbon emissions under the SSP2-4.5 scenario."

Lines 243-291: "*Estimated carbon release from RTSs*"

"*To quantify the current and future impacts of RTS expansion on carbon release across*

the permafrost region of the Tibetan Plateau, we estimated the annual total carbon emissions induced by RTSs by synthesizing CO_2 and CH_4 release rates under three vegetation types, combined with the spatial distribution of RTS susceptibility at high and very high levels (Methods). Our results show that the mean carbon emission rate from RTSs in 2016 was 92.1 ± 0.5 kg C yr^{-1}, corresponding to a total carbon emission of 9.2 ± 2.3 Gg C yr^{-1} (Fig. 4a, c). Among the three vegetation types, alpine meadow contributes the highest proportion (approximately 90.7%), followed by alpine steppe (5.1%) and alpine swamp meadow (4.2%) (Supplementary Table 2). Owing to the expansion of areas with RTS susceptibility, total carbon emissions reach 19.0 ± 4.6 Gg C yr^{-1} by 2022, representing a 1.1-fold increase compared with 2016. Notably, the temporal variation of carbon emissions derived from RTS susceptibility shows high consistency with those estimated from actual RTSs during 2016–2022 (Supplementary Fig. 10), which validates the reliability of the susceptibility-based approach for estimating RTS-induced carbon release.

By 2050, total carbon emissions under the SSP2-4.5 and SSP5-8.5 scenarios will reach 20.1 ± 4.8 Gg C yr^{-1} and 21.3 ± 5.1 Gg C yr^{-1}, respectively, representing a 1.2-fold and 1.3-fold increase compared to the 2016 baseline. By 2100, RTS-induced carbon release under SSP2-4.5 will reach 24.8 ± 5.8 Gg C yr^{-1}, which is 2.7-fold higher than the 2016 (Fig. 4, Supplementary Fig. 11). This study represents the first assessment of potential carbon release associated with the future expansion of RTS across the Tibetan Plateau. Although annual carbon release from RTS-susceptible areas accounts for a small fraction of the net ecosystem carbon sink due to the limited spatial extent of RTS[44], the cumulative impacts on vegetation, carbon dynamics, hydrological processes, and microbial communities warrant heightened attention, given the severe environmental perturbations accompanying RTS development. Furthermore, the harsh alpine environment of the Tibetan Plateau results in extremely slow vegetation recovery following disturbance. Consequently, the expansion of RTSs under climate warming is likely to threaten the ecosystem carbon balance in this high-altitude region. Since 2015, frequent and abrupt cryospheric disturbances have been observed across the Tibetan Plateau[45], indicating that the cryosphere acts as a tipping element under the 1.5 °C global warming threshold[46]. Given that permafrost degradation is expected to accelerate substantially under future climate scenarios[47], there is an urgent need to systematically quantify and predict carbon release driven by abrupt permafrost thaw.

Future changes in the RTS susceptibility and associated carbon release remain highly uncertain, due to pronounced spatial heterogeneity across the mountain permafrost zone of the Tibetan Plateau. This heterogeneity stems from regional variations in precipitation regimes, topographic setting and ground-ice conditions. Such spatial complexity in future RTS dynamics further introduces substantial uncertainties into projections of regional carbon emissions. Despite these uncertainties, this study offers distinct advantages. We compiled a comprehensive dataset comprising 1,862 in-situ measurements of CO_2 and CH_4 emission rates and 291 observations of SOCC loss from a variety of RTSs sites, enabling more robust regional carbon budget estimates across the plateau. Importantly, the dataset incorporates filed observations during the non-growing season, which are critical for constraining estimates of annual carbon release. Furthermore, validation against that from actual observed RTS distributions confirms the reliability of our simulations for carbon release driven by RTS susceptibility. This study provides a solid foundation for future comprehensive assessments of carbon release caused by permafrost degradation under climate change."

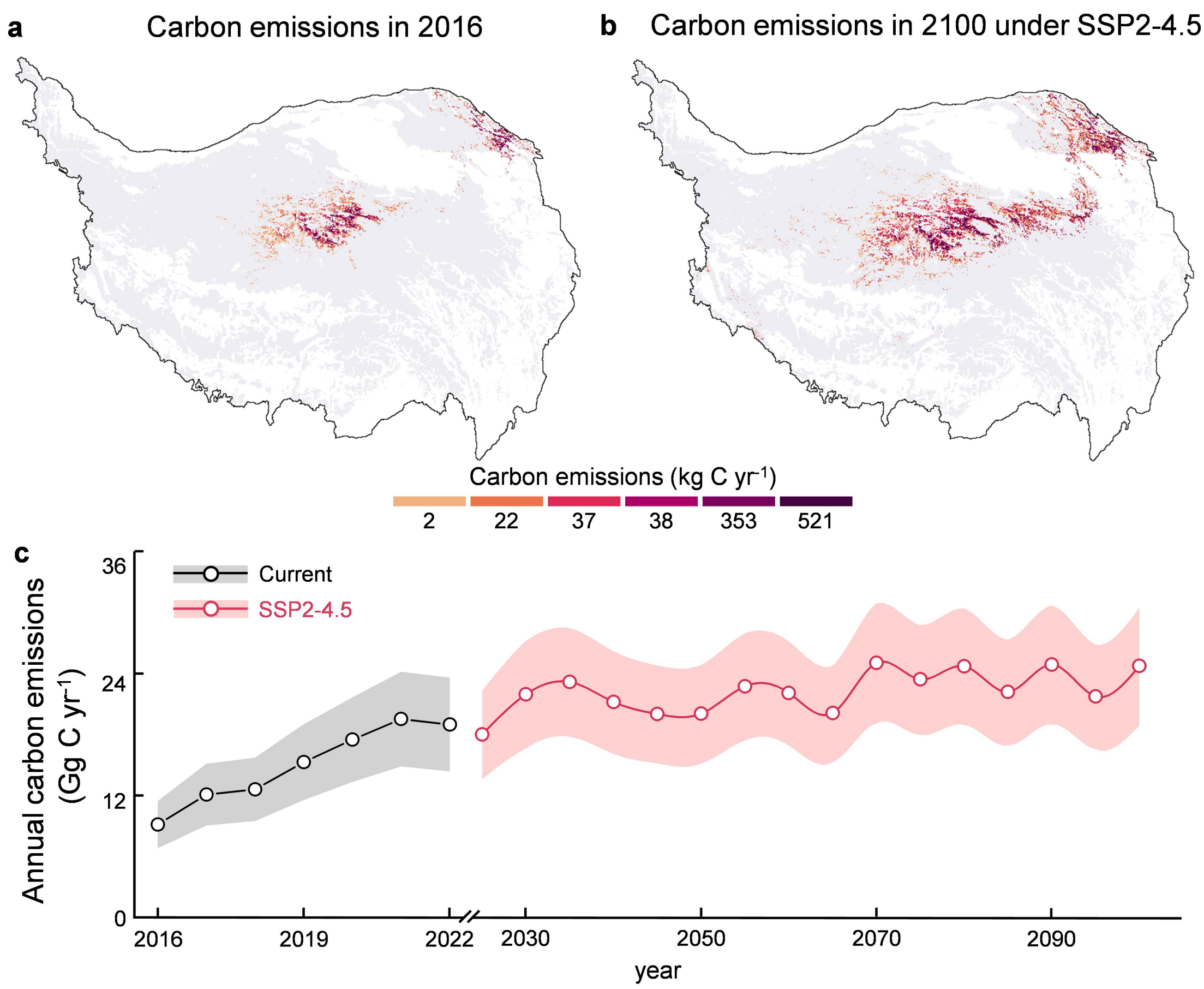

*Fig. 4 | **Carbon emissions from RTS susceptibility areas during 2016-2022. (a-b)** Spatial distribution of carbon emissions from RTS susceptibility in 2016 (a) and 2100 (b) under the SSP2-4.5 scenario. **(c)** Projected annual total carbon emissions from RTS susceptibility under the SSP2-4.5 scenario. The shaded areas denote the standard error.*

If I understand correctly, the authors fitted 50 random forest (RF) models to estimate uncertainty. However, all models are trained on essentially the same empirical dataset. As a result, this approach captures only algorithmic randomness and does not account for data uncertainty (e.g., sampling variability) or model structural uncertainty. Consequently, the reported uncertainty is likely underestimated. A more common approach is to use the distribution of predictions from individual trees within a single random forest to quantify predictive uncertainty.

Response: Thank you for your constructive comment. We agree that uncertainty estimated solely from repeated random forest realizations trained on the same empirical dataset may underestimate the full uncertainty. Following your valuable suggestion, we have revised the uncertainty analysis to quantify predictive variability based on the distribution of individual-tree predictions.

In the revised manuscript, grid-cell level predictive uncertainty is quantified using the standard deviation (SD) of tree-level predictions, while the coefficient of variation (CV) is employed to characterize the relative stability of model predictions. The corresponding revisions have been incorporated into the Methods and Results sections.

Lines 599-625: "*Each forest ensemble comprised 50 decision trees, implemented with the default mtry parameter and a terminal node size of 5. Model calibration was conducted over five evaluation runs, with 75% of samples assigned to model training and the remaining*

25% reserved for validation. The final RTS susceptibility map was generated by averaging predictions across all RF realizations.

RTS susceptibility was spatially projected using the BIOMOD_Projection function in the biomod2 package, which applies the trained RF model to the environmental raster layers to predict the probability of RTS occurrence for each grid cell across the plateau. The resulting raster depicted RTS susceptibility as a relative probability index ranging from 0 to 1. Following an established classification scheme[64], RTS susceptibility was divided into five levels: very low (0–0.2), low (0.2–0.4), moderate (0.4–0.6), high (0.6–0.8), and very high (0.8–1.0). Grid cells with a probability index < 0.2 were defined as control areas, whereas those with an index ≥ 0.2 were classified as RTS-influenced areas.

Predictive uncertainty was quantified based on the distribution of predictions from individual decision trees. Specifically, predictions from all trees within each fitted RF model were extracted to produce tree-level susceptibility rasters across the plateau. As the workflow integrated repeated pseudo-absence generation (10 replicates), repeated data partitioning (5 runs), and 50 trees per RF model, each grid cell received up to 2,500 individual tree-level predictions. For each grid cell, uncertainty was quantified as the standard deviation (SD) of all tree-level predictions; higher SD values indicated greater divergence among predictions, corresponding to higher local uncertainty in RTS susceptibility mapping. We further calculated the coefficient of variation (CV; SD normalized by the mean prediction) to characterize relative predictive variability. The SD represents the absolute predictive uncertainty for each grid cell, while the CV reflects the relative stability of model predictions, with higher CV values indicating greater relative uncertainty in RTS susceptibility mapping (Supplementary Fig. 9)."

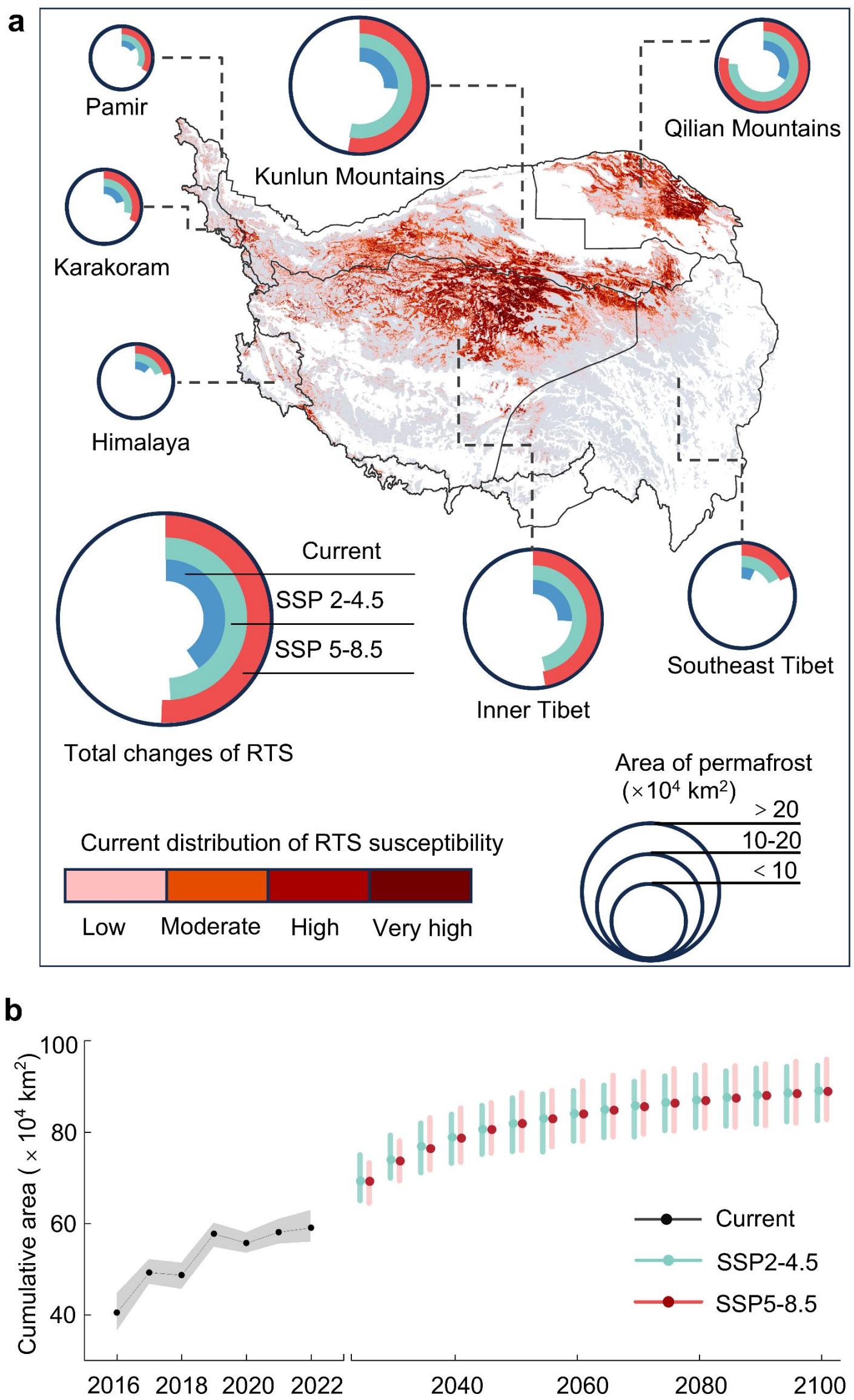

Fig. 3 | Variations in RTS susceptibility. (a) Current spatial distribution of RTS susceptibility, classified into four levels: low, moderate, high, and very high. The donut chart illustrates the areal proportion of RTS-affected regions across different mountain regions; circle size corresponds to the permafrost area within each mountain region, and blue shading denotes the current extent of RTS susceptibility. Green and red arcs represent projected RTS susceptibility areas by 2100 under SSP2-4.5 and SSP5-8.5, respectively. (b) Temporal changes in simulated RTS susceptibility area from 2016 to 2100. The left panel shows area changes derived from observed RTSs during 2016–2022. The right panel shows cumulative area changes within RTS-affected zones under future climate scenarios, with future susceptibility displayed at 5-year intervals for SSP2-4.5 and SSP5-8.5. Points indicate mean values, and thick vertical bars represent uncertainty ranges quantified by the standard deviation. (c-e) Current and projected RTS susceptibility areas by 2100 for low to very high (c), low to moderate (d), and high to very high (e) levels. Error bars represent prediction uncertainty expressed as the standard deviation. Percentages above the bars indicate mean changes in RTS susceptibility area under different climate scenarios.

We highly appreciate the reviewer's constructive comments, which have greatly helped us improve the clarity of the manuscript.

Reviewer #5 (Remarks to the Author):

This study addresses an important and timely question in abrupt permafrost thaw. The work is well-motivated, and the authors succeed in demonstrating the RTS evolution and their induced carbon emissions on the Tibetan Plateau. This manuscript presents a valuable dataset of spatiotemporal distribution of RTS susceptibility using machine learning models. In addition, the carbon modeling builds on the Beer et al. (2023) framework, which first applied a two-pool model to thaw slump carbon release. This extends this approach in several meaningful ways: incorporating soil moisture parameters, conducting vegetation-specific modeling to account for ecological heterogeneity, and validating the model against field measurements. In addition, the dataset of 1,826 monitoring records, which includes non-growing season observations across three vegetation types, provides a solid empirical basis for this validation.

The revised manuscript has been improved through the detailed responses to all comments. I believe this manuscript will make a meaningful contribution to the field of permafrost change. I thus recommend acceptance after a minor revision. The following minor issues could be addressed to further improve clarity:

Response: We greatly appreciate the reviewer's highly positive assessment of this work. We are also thankful for the favorable comments on our observational dataset, particularly the inclusion of non-growing season measurements across three vegetation types, which establishes an important empirical foundation. We address each of the reviewer's comments point by point below.

1. For the monitored 61 RTS sites, the discussion about the differences in carbon losses caused by RTS in alpine swamp meadows and steppes should be strengthened, in order to clarify the impacts on soil carbon contents.

Response: We have strengthened the discussion on vegetation-dependent differences in carbon loss, particularly between alpine swamp meadows and alpine steppes.

Lines 105-110: "*Alpine steppes exhibit relatively low carbon loss, despite experiencing dramatic hydrothermal changes during RTS development (a 65.3% increase in soil temperature and a 238.9% decrease in moisture; Supplementary Fig.3). By contrast, alpine swamp meadows, contain much higher soil carbon stocks and thus exhibit greater carbon loss despite, even with milder hydrothermal alterations. These contrasting patterns highlight that vegetation type strongly mediates soil carbon loss induced by RTSs.*"

2. In the results and discussion, the CH_4 release rate induced by RTSs focuses on the alpine swamp meadow; other vegetation types should be shown or the relevant data should be introduced.

Response: Thank you for this constructive suggestion. Due to the sparse availability of CH_4 flux measurements during RTS development across the Tibetan Plateau, our analysis focuses solely on CH_4 release rates in alpine swamp meadows. To avoid any potential confusion, we have clarified this point in the caption of Figure 1 by adding the statement: "*Notably, CH_4 flux data are focused on alpine swamp meadows.*"

3. Non-growing season estimation: The use of fixed seasonal ratios (CO_2: 0.38; CH_4: 0.18) to estimate non growing season release is a practical approach. A brief note on whether these ratios showed variability across vegetation types or monitoring years would provide helpful context.

Response: Thank you for the detailed comments. Following the suggestion from Reviewer 4, we have updated our methodological framework by scaling observed carbon release rates

across different vegetation types with RTS-susceptible areas predicted by the random forest (RF) model. This approach circumvents the structural limitations inherent in process-based carbon models. Non-growing season carbon release is now directly estimated from field monitoring data, thereby eliminating the need for any assumed seasonal scaling ratios.

Lines 670-681: "*Estimation of carbon release from RTSs*"

"*We quantified the total regional carbon release from three vegetation types (derived from monitoring data) by multiplying their mean carbon release rates by the area of RTS susceptibility*[72]*, where high and very high susceptibility classes were defined as the RTS-affected region. An area density ratio was used to represent the actual RTS-affected area within each vegetation type, and uncertainties were propagated from the standard errors of both carbon release rate and area density estimates. Potential overestimation was corrected using the mean RTS area density for each vegetation type over the 2016-2022 period (Equation 1; Supplementary Table 6). The reliability of this susceptibility-based approach was validated by comparing its estimated annual carbon emissions with those derived from actual RTSs during the same period (Supplementary Fig. 10). The validated framework was subsequently applied to project future RTS-driven carbon emissions under the SSP2-4.5 scenario.*

"

4. In the figure 3b, it is necessary to clarify what the shaded areas represent at current and in the future.

Response: We have clarified this in the figure caption by specifying that the points represent mean values, while the thick vertical bars indicate uncertainty ranges quantified by the standard deviation.

5. The manuscript does not provide a comparison between the carbon release rate (46% concentrated between 100 and 150 g C m^{-2} yr^{-1}) mentioned and the carbon release from soil respiration during gradual permafrost warming. It would be beneficial to clarify the potential of carbon emissions caused by RTSs.

Response: Thank you for this suggestion. We agree that comparing RTS-induced carbon release with carbon release from gradual permafrost warming would be valuable for understanding the relative importance of abrupt thaw processes. However, following the removal of the two-pool carbon model (see response to the previous comment), the comparison between RTS-induced carbon release and gradual permafrost warming is no longer included in the revised manuscript.

6. Supplementary Figure 16 shows the model data comparison for CO_2 release rates. Clarify whether the data points represent daily values, seasonal averages, or monthly means. Additionally, specifying which seasons or months are included would help readers better interpret the validation results.

Response: Thank you for this comment. Since the two-pool carbon model has been removed from the revised manuscript (see response above), Supplementary Figure 16 is no longer applicable and has been deleted accordingly.

7. Model validation: The authors report strong validation metrics (R^2=0.915, RMSE=0.915 g C m^{-2} d^{-1}). Clarify whether these metrics represent performance across all vegetation types combined or were calculated separately for each type.

Response: Thank you for this comment. Following the removal of the two-pool carbon model (as described above), the model validation metrics (R^2 and RMSE) are no longer applicable and have been removed from the revised manuscript.

8. There are some uncertainties in future projection of carbon emissions due to high spatial heterogeneity, and it would be valuable to add some discussion on the simulated changes in RTS on the Tibetan Plateau.

Response: Thank you for your valuable comment. We have added a discussion addressing the uncertainties surrounding future changes in RTS susceptibility and carbon release across the Tibetan Plateau, particularly with respect to regional variations in precipitation regimes, topographic settings, and ground-ice conditions.

Lines 278-283: "*Future changes in the RTS susceptibility and associated carbon release remain highly uncertain, due to pronounced spatial heterogeneity across the mountain permafrost zone of the Tibetan Plateau. This heterogeneity stems from regional variations in precipitation regimes, topographic setting and ground-ice conditions. Such spatial complexity in future RTS dynamics further introduces substantial uncertainties into projections of regional carbon emissions.*"

Lines 316-335: "*Despite synthesizing 4,728 RTS incidents and 1,862 in-situ observations of carbon release rates, this study remains subject to multiple sources of uncertainty. First, the predicted spatial susceptibility of RTS is potentially influenced by uncertainties in the RTS inventory, environmental predictors, model parameterization, and assumptions under future climate scenario. Second, the developmental stage of RTSs was not incorporated into our estimation of carbon loss. As RTSs evolve, surface soil organic carbon loss tends to increase during early developmental stages and subsequently decreases[18]. In addition, RTSs development can cause substantial displacement and accumulation of deep soils[13], influencing soil porosity and microbial communities[49] and thereby modulating carbon release. Consequently, there is an increasing need for future work to develop integrated carbon process models that account for the multi-factorial dynamics of abrupt thaw. Given the spatial heterogeneity in ground ice distribution[50], the development of some RTSs may gradually stabilize, potentially enabling slow vegetation regrowth in affected areas[34], which could further influence regional carbon source-sink dynamics[51,52]. Furthermore, the three-dimensional complexity of abrupt thaw necessitates a holistic framework to quantify carbon release across the entire process of RTS development. For instance, the volume of the collapsed body changes dynamically throughout RTS evolution, directly influencing real-time carbon release fluxes[53]. Improving process-based simulations of RTS-induced carbon release on the Tibetan Plateau can provide valuable insights for other mountain permafrost regions to systematically estimate permafrost carbon-climate feedbacks.*
"

We sincerely appreciate the reviewers' positive, valuable and detailed suggestions. Hope our careful revisions adequately improved the manuscript. Thank you very much.
